# Influence of taping on joint proprioception: a systematic review with between and within group meta-analysis

**DOI:** 10.1186/s12891-024-07571-2

**Published:** 2024-06-18

**Authors:** Shashank Ghai, Ishan Ghai, Susanne Narciss

**Affiliations:** 1 Department of Political, Historical, Religious and Cultural Studies, Karlstads Universitet, Karlstad, Sweden; 2 Centre for Societal Risk Research, Karlstads Universitet, Karlstad, Sweden; 3 Psychology of Learning and Instruction, Department of Psychology, School of Science, Technische Universität Dresden, Dresden, Germany; 4 Centre for Tactile Internet With Human-in-the-Loop (CeTI), Technische Universität Dresden, Dresden, Germany; 5 School of Life Sciences, Jacobs University Bremen, Bremen, Germany

**Keywords:** Tape, Position sense, Proprioception, Rehabilitation, Injury

## Abstract

Taping is increasingly used to manage proprioceptive deficits, but existing reviews on its impact have shortcomings. To accurately assess the effects of taping, a separate meta-analyses for different population groups and tape types is needed. Therefore, both between- and within-group meta-analyses are needed to evaluate the influence of taping on proprioception. According to PRISMA guidelines, a literature search was conducted across seven databases (Web of Science, PEDro, Pubmed, EBSCO, Scopus, ERIC, SportDiscus, Psychinfo) and one register (CENTRAL) using the keywords “tape” and “proprioception”. Out of 1372 records, 91 studies, involving 2718 individuals, met the inclusion criteria outlined in the systematic review. The meta-analyses revealed a significant between and within-group reduction in repositioning errors with taping compared to no tape (Hedge’s g: -0.39, *p* < 0.001) and placebo taping (Hedge’s g: -1.20, *p* < 0.001). Subgroup and sensitivity analyses further confirmed the reliability of the overall between and within-group analyses. The between-group results further demonstrated that both elastic tape and rigid tape had similar efficacy to improve repositioning errors in both healthy and fatigued populations. Additional analyses on the threshold to detection of passive motion and active movement extent discrimination apparatus revealed no significant influence of taping. In conclusion, the findings highlight the potential of taping to enhance joint repositioning accuracy compared to no tape or placebo taping. Further research needs to uncover underlying mechanisms and refine the application of taping for diverse populations with proprioceptive deficits.

**Supplementary Information:**

The online version contains supplementary material available at 10.1186/s12891-024-07571-2.

## Introduction

Over the past decade, taping has become a focal point in rehabilitation and performance science [1]. The increased application of this intervention is partly fueled by its enhanced viability [2], ease of application [3], availability [4], cost-effectiveness [5, 6], and sometimes just a fashion statement [7, 8]. Owing to these factors, the application of taping in the existing literature extends endlessly across different medical conditions [9–16], and sports [17–19].

The literature has proposed several mechanisms to explain the effects of taping [14, 20–24]. However, enhancing joint proprioception is one of the significant mechanisms of taping that is considered to facilitate recovery and performance [25, 26]. Proprioception refers to an individual's ability to integrate sensory input from mechanoreceptors within musculoskeletal structures to determine the position of a body segment in space [27]. Deficiencies in proprioception are known to negatively affect joint biomechanics and neuromuscular control, increasing the risks of injury [28–30]. Therefore, stimulating proprioception during training is crucial for rehabilitation, as its enhancement could not only promote coordinated movements, joint stability, and control, but also reduce the likelihood of injuries [31–33]. In the context of taping, research has indicated that the tactile stimulation from taping can activate mechanoreceptors that eventually augment the afferent input via the central pathways to augment proprioception [34–36]. Eventually, this increased afferent input is thought to further facilitate the efferent neuromuscular response, which increases both the speed and the quality of the muscle reaction [37–39]. Y Konishi [34] suggested that injury-induced damage to musculoskeletal structures could impair proprioception by deteriorating mechanoreceptors' ability to provide regular afferent feedback, crucial for modulating motor units. Under these conditions, tactile stimulation via taping might rescue alpha motor activity [34], while a "skin stretch" effect from taping could enhance proprioception by altering musculoskeletal kinetics [40–43]. Taping has demonstrated benefits in improving proprioception and preventing injuries by enhancing joint position sense and resisting harmful movements [44–46]. Additionally, taping may boost motor performance by enhancing neural activity, as shown in neuroimaging studies indicating increased activation in brain areas related to coordination and sensation [14, 47, 48].

Despite this mounting evidence suggesting the beneficial influence of taping on joint proprioception and its gaining popularity, a lack of consensus exists in the literature regarding its efficacy. For instance, while some individual trials have suggested the beneficial influence of taping on proprioception [49–56], others have suggested no effect [57–63], or even a detrimental effect [64–68]. Similarly, reviews [23, 63, 69, 70], and meta-analyses [71, 72], have reported inconclusive evidence regarding the overall efficacy of taping on joint proprioception. Within the meta-analyses, while one has reported no effect of taping on proprioception in people with a recurrent ankle sprain [71], the other reported a beneficial influence of taping on ankle repositioning in the same population group [72]. Likewise, the four systematic reviews also stated an inconclusive impact of tape on proprioceptive performance [23, 63, 69, 70].

Besides the mixed findings, several limitations of the existent meta-analyses warrant an improved systematic review and meta-analysis [73]. The existing reviews are limited from both analytical and methodological points of view on several accounts. First, these reviews do not include several existing high-quality trials [48–52, 57, 64, 74–83]. This lack of sufficient data could diminish the power of these meta-analyses and increase the probability that the observed results occurred due to a type II error. Second, none of these reviews conducted both between- and within-group meta-analyses. These findings could be significant because the between-group analyses can explain the differential outcome of taping compared to no-tape/placebo tape. In contrast, the within-group analyses could explain the magnitude of change in proprioceptive parameters before and after the taping. Findings on both, between- and within-group effects are needed to deduce appropriate training dosages or perform comparative evaluations with existing interventions in their training regimens. Third, it was observed that none of the existing meta-analyses analyzed the results differently among randomized controlled and controlled clinical trials. Such a differential analysis would allow for the classification of studies in a meta-analysis according to their inherent level of bias. Fourth, no review has differentiated the outcomes of taping according to an individual's health status. The two meta-analyses published to date have only evaluated the influence of taping on individuals with ankle instability [71, 72]. Even though trials have reported the impact of taping among healthy individuals and individuals with stroke [77, 84], osteoarthritis [85, 86], anterior cruciate ligament injury/reconstruction [78, 87, 88], no review has attempted to differentially synthesize the efficacy of taping according to the health status of an individual. Evaluating this outcome is important to quantify the effectiveness of taping in different health conditions and could be helpful for both clinicians and patient population groups. Finally, no review has yet examined how the elasticity of tapes, including elastic and rigid tape, influences joint proprioceptive performance [89, 90]. Elastic tapes, such as Kinesio and dynamic tape, are known to enhance proprioception due to their high stretch capabilities, allowing them to move and stretch with the body's natural movements and provide constant feedback to sensory receptors in the skin and underlying tissues [91]. In contrast, rigid tapes like athletic and Leuko tape prioritize support and stability over range of motion (47). A comparative assessment of elastic and rigid tapes could offer useful insights for clinicians, patients, and tape manufacturers on how tensile strength affects proprioceptive performance.

### Research aims and questions

In this systematic review and meta-analysis, a between-group analysis (i.e., taping vs placebo/no tape) and a within-group (i.e., pre-vs post-test) was conducted to determine the influence taping has on proprioception in healthy and patient population groups. The goal of the study is to allow clinicians to understand tape's overall impact while simultaneously allowing them to compare its efficacy with existing interventions. The main aims of this study are:

1. To evaluate the effect of taping on repositioning accuracy from between- and within-group analyses.
2. To evaluate the effect of taping on the threshold to detect passive movement from both between- and within-group analyses.
3. To evaluate the effect of taping on active movement extent discrimination accuracy from between- and within-group analyses.
4. To perform subgroup meta-analyses between individual studies according to the elasticity of tape (i.e., elastic, rigid tape), health status (i.e., healthy, patient population groups), and study design (i.e., randomized and non-randomized trials).

## Material and methods

The PRISMA-SR 2020 guidelines were followed to conduct this systematic review and meta-analysis. The checklist is presented in Table S1. This systematic review was pre-registered at the PROSPERO (CRD42022344452).

### Sources of data and search strategy

The systematic literature search was performed across seven databases (Web of Science, PEDro, Pubmed, EBSCO, Scopus, ERIC, SportDiscus, Psychinfo) and one register (Cochrane Central Register of Controlled Trials) for the publication period from January 1970 until August 2023. These databases were chosen on the basis of access provided by the academic organization. The authors also conducted an extra search of the reference section of the included studies.

The review's criteria for study inclusion were established following the PICOS approach (Population, Intervention, Comparator, Outcome of Interest, Study Design). Two researchers (S.G, I.G) determined the inclusion criteria, which were as follows:

1. Healthy population groups.
2. Population groups with musculoskeletal disorders (e.g., sprains, strains, tendinitis, repetitive stress injuries, degenerative joint diseases, traumatic injuries).
3. Population groups with neurological disorders (e.g., stroke, Parkinson's disease, cerebral palsy, multiple sclerosis, traumatic brain injury, degenerative neurological disorders).
4. Studies assessing the impact of taping on joint proprioception.
5. Proprioception acuity evaluated through joint repositioning tests, threshold to detect passive motion (TTDPM), active movement extent discrimination apparatus (AMEDA) (for detailed test explanations, refer to [27]).
6. Studies comparing taping intervention outcomes with no taping or placebo tape.
7. All types of quantitative clinical studies, including randomized controlled trials, controlled clinical trials, crossover trials, cross-sectional studies, cohort studies, and feasibility studies.
8. Studies published in peer-reviewed academic journals, theses, and conference proceedings.
9. Studies published in English, French, German, or Hindi.

Two authors independently screened the titles, abstracts, and full texts of the articles. In instances of disagreement regarding the selection of pertinent studies, the two authors engaged in discussions. The subsequent data were extracted from the articles: author names, country of research, participant details (age, sample size, gender distribution, health status), evaluated joint, taping method, taping technique, taping applicator, assessment durations, taping frequency, and outcomes.

### Assessment of the methodological quality

The quality of the studies included in the review was assessed using the PEDro quality appraisal scale [92]. The interpretation of PEDro scale scores is as follows: studies scoring between 9 to 11 are considered "excellent quality," 6 to 8 are deemed "good quality," 4 to 5 are classified as "fair quality," and scores less than or equal to 3 are labeled "poor quality" [93]. Two researchers (SG, IG) independently conducted the appraisal of the studies.

### Data analysis

In the present review, a between-group (taping vs. no taping comparator and taping vs. placebo comparator) and a within-group (pre- vs. post-taping) random effect meta-analysis was conducted with Comprehensive meta-analysis (V 4.0) [94]. The data for the meta-analysis was separately distributed and analyzed for each proprioceptive outcome (i.e., re-positioning accuracy, the threshold to detect motion passively, active movement extent discrimination accuracy). Furthermore, subgroup analyses were conducted based on study design (i.e., randomized controlled trial, controlled clinical trial), type of taping (i.e., elastic, rigid tape), and health status (i.e., healthy, musculoskeletal injury, neurological injury), and health status receiving different types of tape (elastic/rigid). The reported outcomes of the meta-analysis included weighted and adjusted effect size (i.e., Hedge’s g), 95% C.I., and significance level. The threshold for the interpretation of effect size is as follows: > 0.16 to < 0.38 is considered a small effect, ≥ 0.38 to < 0.76 as a medium effect, and ≥ 0.76 as a large effect [95]. Forest plots were generated to illustrate the overall results.

Besides, the presence of heterogeneity was assessed using I^2^ statistics. The threshold for interpreting the heterogeneity with I^2^ statistics is as follows: between 0 to 25% considered negligible heterogeneity, 25% to 75% as moderate heterogeneity, and > 75% as substantial heterogeneity [96]. Additionally, “leave-one-out” sensitivity analyses were conducted to test the robustness of the findings. The method systematically removes each study from the meta-analysis and re-analyzes the data to assess the influence of individual studies on the overall results. This helps to identify studies that may be driving the results and assess the robustness of the findings [97]. Additionally, an assessment of publication bias was carried out according to the trim and fill procedure by Duval and Tweedie [98]. The alpha level for the study was set at 5%.

## Results

### Characteristics of included studies

**Fig. 1:**
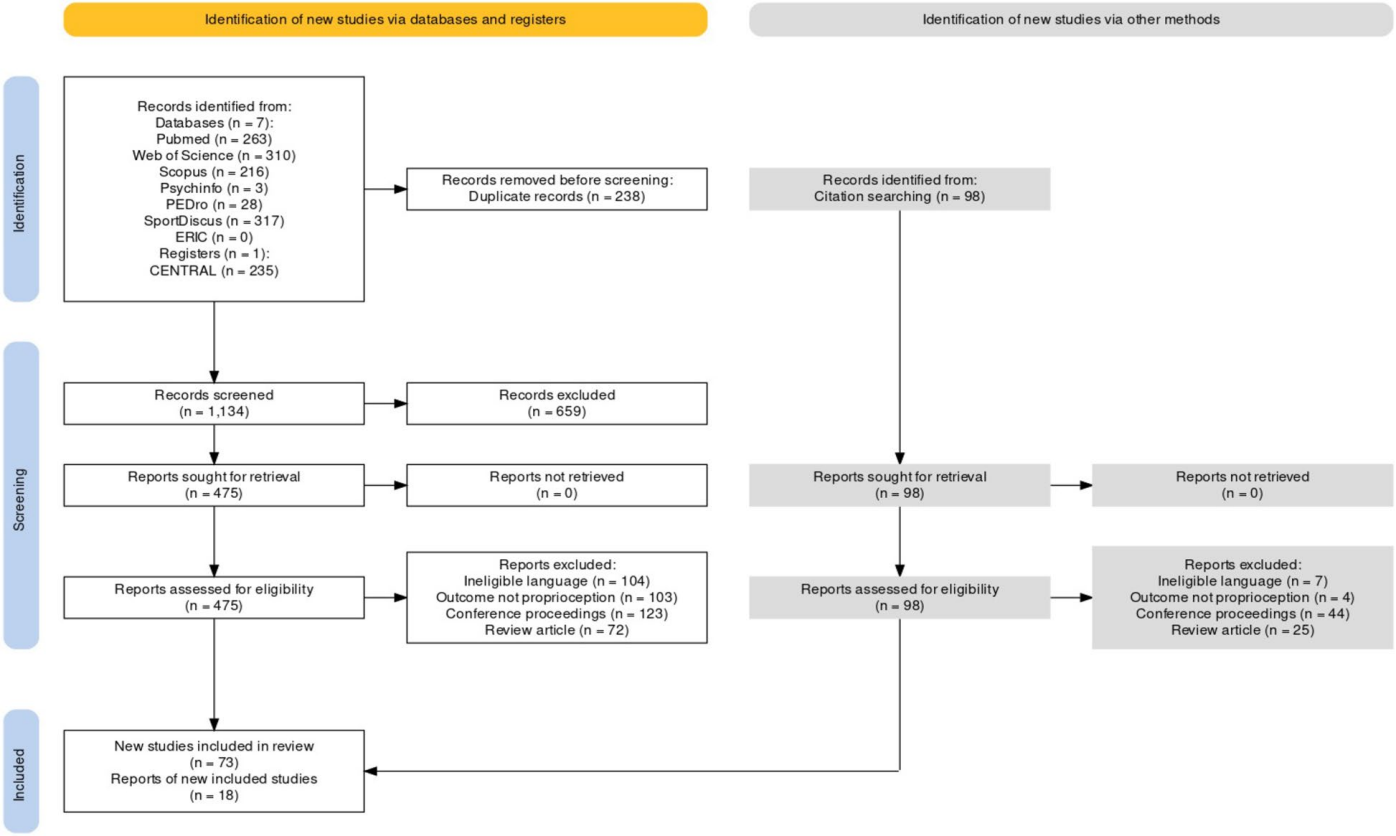
PRISMA flowchart (developed from an R package and Shiny application from [99])

The initial search across the seven databases and one registry yielded a total of 1372 articles, which after implementing the PICOS inclusion criteria, were reduced to 73 articles. Furthermore, during the examination of the citations within these included articles, 98 relevant articles were encountered. These additional articles underwent another round of screening, ultimately resulting in the inclusion of another 18, in total 91 articles. A PRISMA flow chart illustrates the entire selection process in Fig. 1 [99]. Thereafter, qualitative data were extracted from all included studies.

### Study design

Of the 91 included studies (Table S 2), 35 were randomized controlled trials [54, 55, 57, 60, 62, 67, 74–78, 80, 82, 83, 85, 100–119], 29 were randomized cross over design [48, 51, 52, 56, 59, 61, 66, 81, 84, 90, 120–138], 12 were pre-test post-test quasi experimental design [53, 64, 139–148], 10 were crossover trials [36, 39, 50, 58, 68, 88, 149–152], two were non-randomized controlled design [65, 153], one was a cross-sectional design [154], and one was a retrospective cohort study [87]. Additionally, one study presented data from two different studies in which one was a pre-test post-test quasi experimental design and a randomized controlled design [86].

### Risk of *bias*

**Fig. 2:**
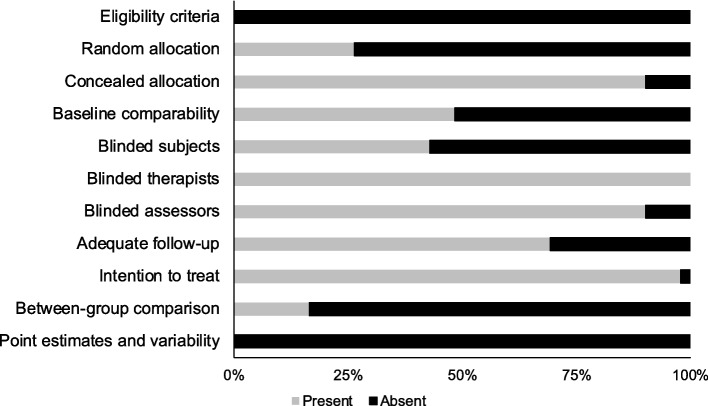
Illustrating the presence/absence of risk of bias according to the PEDro scale

The individual PEDro scoring for each included study has been tabulated in Table 1. The average PEDro quality score of the 91 included studies was (5.2 ± 1.6), suggesting the overall quality of the included studies to be “fair”. Individually, 3% of studies scored 9, 5% scored 8, 10% scored 7, 26% scored 6, 22% scored 5, 18% scored 4, 11% scored 3, and 4% scored 2. As mentioned before, one study presented data from two different study in which one was a pre-test post-test quasi experimental design and a randomized controlled design [86]. The risk for bias for this study was appraised as 3 for the pre-test post-test quasi experimental design, and 8 for the randomized controlled design. The risk of bias scoring across the studies has also been illustrated in Fig. 2.

**Table 1:** PEDro scoring for the included studies (**+ **: Risk of bias present, **-**: Risk of bias absent)

No		Total PEDro score	Point estimates and variability	Between-group comparison	Intention to treat	Adequate follow-up	Blinded assessors	Blinded therapists	Blinded subjects	Baseline comparability	Concealed allocation	Random allocation	Eligibility criteria
1	F Fazli, A Farsi, IE Takamjani, S Mansour, N Yousefi and F Azadinia [103]	8	**-**	**-**	** + **	**-**	**-**	** + **	** + **	**-**	**-**	**-**	**-**
2	EP Kisa and BK Kaya [149]	4	**-**	**-**	** + **	** + **	** + **	** + **	** + **	**-**	** + **	** + **	**-**
3	İ Poyraz and Ö Vergili [140]	4	**-**	** + **	** + **	**-**	** + **	** + **	**-**	** + **	** + **	** + **	**-**
4	M Maqsood and M Váczi [126]	4	**-**	**-**	** + **	** + **	** + **	** + **	** + **	** + **	** + **	**-**	**-**
5	F Saki, A Shayesteh, F Ramezani and S Shahheidari [108]	5	**-**	**-**	** + **	** + **	** + **	** + **	**-**	**-**	** + **	**-**	**-**
6	F Shams, M Hadadnezhad, A Letafatkar and J Hogg [110]	5	**-**	**-**	** + **	** + **	** + **	** + **	** + **	**-**	** + **	**-**	**-**
7	P Mehta, AJ Prabhakar and C Eapen [70]	7	**-**	**-**	** + **	**-**	** + **	** + **	**-**	**-**	** + **	**-**	**-**
8	D Kielė and R Solianik [72]	8	**-**	**-**	** + **	**-**	**-**	** + **	** + **	**-**	**-**	**-**	**-**
9	JH Kim, KH Kim and DH Kim [71]	7	**-**	**-**	** + **	**-**	** + **	** + **	**-**	**-**	** + **	**-**	**-**
10	P Li, Z Wei, Z Zeng and L Wang [124]	6	**-**	**-**	** + **	** + **	** + **	** + **	**-**	**-**	** + **	**-**	**-**
11	H–S Chen, Y–Z Chang, C-M Fang, C-Y Lin and W–C Yang [44]	3	**-**	**-**	** + **	** + **	** + **	** + **	** + **	** + **	** + **	** + **	**-**
12	M Saran, S Pawaria and S Kalra [68]	6	**-**	**-**	** + **	**-**	** + **	** + **	** + **	**-**	** + **	**-**	**-**
13	F Saki, H Romiani, M Ziya and N Gheidi [69]	6	**-**	**-**	** + **	** + **	** + **	** + **	**-**	**-**	** + **	**-**	**-**
14	CG Bayu, M Andriana and A Pawana [58]	3	**-**	** + **	** + **	**-**	** + **	** + **	** + **	** + **	** + **	** + **	**-**
15	HE Göktaş, S Çitaker and ED Yurtsever [51]	6	**-**	**-**	** + **	**-**	** + **	** + **	**-**	** + **	** + **	**-**	**-**
16	K Grütters, S Narciss, SM Beaudette and L Oppici [104]	5	**-**	**-**	** + **	** + **	** + **	** + **	** + **	**-**	** + **	**-**	**-**
17	C Boonkerd, K Thinchuangchan, N Chalarak, S Thonpakorb, R Wanasoonthontham, T Kitsuksan and T Laddawong [116]	6	**-**	**-**	** + **	** + **	** + **	** + **	**-**	**-**	** + **	**-**	**-**
18	Y-S Chen, W–C Tseng, C-H Chen and Y-X Lu [75]	6	**-**	**-**	** + **	**-**	** + **	** + **	**-**	** + **	** + **	**-**	**-**
19	R Adams, C Ganderton, J Han, G Waddington, J Witchalls and Z Yang [46]	4	**-**	**-**	** + **	** + **	** + **	** + **	** + **	** + **	** + **	**-**	**-**
20	R Yu, Z Yang, J Witchalls, R Adams, G Waddington and J Han [45]	7	**-**	**-**	** + **	** + **	** + **	**-**	** + **	**-**	**-**	**-**	**-**
21	E Smyth, G Waddington, J Witchalls, P Newman, J Weissensteiner, S Hughes, T Niyonsenga and M Drew [74]	7	**-**	**-**	** + **	** + **	**-**	** + **	** + **	**-**	**-**	**-**	**-**
22	M Alawna, B Unver and E Yuksel [77]	8	**-**	**-**	**-**	**-**	** + **	** + **	**-**	**-**	** + **	**-**	**-**
23	F Binaei, R Hedayati, M Mirmohammadkhani, C Taghizadeh Delkhoush and R Bagheri [76]	5	**-**	**-**	** + **	**-**	** + **	** + **	**-**	** + **	** + **	**-**	**-**
24	Z-M Lin, J-F Yang, Y-L Lin, Y-C Cheng, C-T Hung, C-S Chen and L-W Chou [42]	4	**-**	**-**	** + **	** + **	** + **	** + **	** + **	** + **	** + **	**-**	**-**
25	ME Ucuzoglu, B Unver, DC Sarac and G Cilga [114]	6	**-**	**-**	** + **	**-**	** + **	** + **	** + **	**-**	** + **	**-**	**-**
26	J-T Han [119]	4	**-**	**-**	** + **	** + **	** + **	** + **	** + **	** + **	** + **	**-**	**-**
27	A Trost [113]	6	**-**	**-**	** + **	**-**	** + **	** + **	** + **	**-**	** + **	**-**	**-**
28	KA Alahmari, RS Reddy, JS Tedla, PS Samuel, VN Kakaraparthi, K Rengaramanujam and I Ahmed [97]	9	**-**	**-**	** + **	**-**	**-**	** + **	**-**	**-**	**-**	**-**	**-**
29	S Abbasi, M-R Hadian Rasanani, N Ghotbi, GR Olyaei, A Bozorgmehr and O Rasouli [95]	9	**-**	**-**	** + **	**-**	**-**	** + **	**-**	**-**	**-**	**-**	**-**
30	F Babakhani, M Heydarian and M Hatefi [101]	5	**-**	**-**	** + **	** + **	** + **	** + **	** + **	**-**	** + **	**-**	**-**
31	S-Y Park, M-J Kim, S-E Seol, C Hwang, J-S Hong, H Kim and W-S Shin [127]	5	**-**	**-**	** + **	** + **	** + **	** + **	**-**	** + **	** + **	**-**	**-**
32	M Alawna and AA Mohamed [98]	9	**-**	**-**	**-**	**-**	** + **	** + **	**-**	**-**	**-**	**-**	**-**
33	H Shahrokhi, H Miri and S Yekedehghan [109]	5	**-**	**-**	** + **	** + **	** + **	** + **	** + **	**-**	** + **	**-**	**-**
34	Z Wei, X-X Wang and L Wang [132]	6	**-**	**-**	** + **	**-**	** + **	** + **	**-**	** + **	** + **	**-**	**-**
35	B Rajabzadeh, A Amiri, B Vasaghi-Gharamaleki and SH Saneii [141]	4	**-**	** + **	** + **	**-**	** + **	** + **	**-**	** + **	** + **	** + **	**-**
36	M Dhahi and MS Abdelsalam [48]	6	**-**	**-**	** + **	** + **	** + **	** + **	**-**	**-**	** + **	**-**	**-**
37	I Narasinta, RH Masduchi and PM Kurniawati [47]	3	**-**	** + **	** + **	** + **	** + **	** + **	**-**	** + **	** + **	** + **	**-**
38	K Liu, J Qian, Q Gao and B Ruan [81]	3	**-**	** + **	** + **	**-**	** + **	** + **	** + **	** + **	** + **	** + **	**-**
39	CM Brogden, K Marrin, RM Page and M Greig [50]	5	**-**	**-**	** + **	** + **	** + **	** + **	**-**	** + **	** + **	**-**	**-**
40	NHN Allah, GA Mohamed, SM Elhafez and IM Emran [99]	6	**-**	**-**	** + **	**-**	** + **	** + **	** + **	**-**	** + **	**-**	**-**
41	L Bischoff, C Babisch, J Babisch, F Layher, K Sander, G Matziolis, S Pietsch and E Röhner [82]	4	**-**	**-**	** + **	** + **	** + **	** + **	** + **	**-**	** + **	** + **	**-**
42	YF Shih, YF Lee and WY Chen [111]	7	**-**	**-**	** + **	**-**	** + **	** + **	**-**	**-**	** + **	**-**	**-**
43	H Momeni-lari, M Ghasemi, K Khademi-kalantari and A Akbarzadeh-baghban [138]	3	**-**	** + **	** + **	** + **	** + **	** + **	**-**	** + **	** + **	** + **	**-**
44	A Jahjah, D Seidenspinner, K Schüttler, A Klasan, TJ Heyse, D Malcherczyk and BF El-Zayat [49]	6	**-**	**-**	** + **	** + **	** + **	** + **	**-**	**-**	** + **	**-**	**-**
45	SH Cho and HJ Moon [136]	2	**-**	** + **	** + **	** + **	** + **	** + **	** + **	** + **	** + **	** + **	**-**
46	KA Keenan, JS Akins, M Varnell, J Abt, M Lovalekar, S Lephart and TC Sell [106]	6	**-**	**-**	** + **	** + **	** + **	** + **	**-**	**-**	** + **	**-**	**-**
47	N Weerakkody and T Allen [131]	6	**-**	**-**	** + **	** + **	** + **	** + **	**-**	**-**	** + **	**-**	**-**
48	LM Wilson and M Greig [52]	5	**-**	**-**	** + **	** + **	** + **	** + **	**-**	**-**	** + **	** + **	**-**
49	D Bailey and P Firth [115]	4	**-**	** + **	** + **	** + **	**-**	** + **	** + **	** + **	** + **	**-**	**-**
50	Y-S Bae [135]	2	**-**	** + **	** + **	** + **	** + **	** + **	** + **	** + **	** + **	** + **	**-**
51	GLd Santos, MB Souza, K Desloovere and TL Russo [78]	5	**-**	**-**	** + **	** + **	** + **	** + **	**-**	** + **	** + **	**-**	**-**
52	Z Long, R Wang, J Han, G Waddington, R Adams and J Anson [84]	5	**-**	**-**	** + **	** + **	** + **	** + **	** + **	**-**	** + **	**-**	**-**
53	SA Ruggiero, LR Frost, LA Vallis and SHM Brown [59]	5	**-**	**-**	** + **	**-**	** + **	** + **	** + **	**-**	** + **	** + **	**-**
54	R Torres, R Trindade and RS Gonçalves [112]	6	**-**	**-**	** + **	**-**	** + **	** + **	** + **	**-**	** + **	**-**	**-**
55	EE Kurt, Ö Büyükturan, HR Erdem, F Tuncay and H Sezgin [107]	7	**-**	**-**	** + **	**-**	** + **	** + **	**-**	**-**	** + **	**-**	**-**
56	H–D Seo, M-Y Kim, J-E Choi, G-H Lim, S-I Jung, S–H Park, S–H Cheon and H-Y Lee [142]	3	**-**	** + **	** + **	** + **	** + **	** + **	**-**	** + **	** + **	** + **	**-**
57	M Akbari, G Pahnabi and H Karimi [134]	2	**-**	** + **	** + **	** + **	** + **	** + **	** + **	** + **	** + **	** + **	**-**
58	IK Ahn, YL Kim, Y-H Bae and SM Lee [96]	6	**-**	**-**	** + **	** + **	** + **	** + **	**-**	**-**	** + **	**-**	**-**
59	Z Barzegar Ganji, F Dehghan-Manshadi, K Khademi-Kalantari, M Ghasemi and SM Tabatabaee [148]	3	**-**	**-**	** + **	** + **	** + **	** + **	** + **	** + **	** + **	** + **	**-**
60	LM Aarseth, DN Suprak, GR Chalmers, L Lyon and DT Dahlquist [60]	4	**-**	**-**	** + **	** + **	** + **	** + **	** + **	** + **	** + **	**-**	**-**
61	H-y Cho, E–H Kim, J Kim and YW Yoon [79]	6	**-**	**-**	** + **	** + **	** + **	** + **	**-**	**-**	** + **	**-**	**-**
62	SM Burfeind and N Chimera [102]	6	**-**	**-**	** + **	** + **	** + **	** + **	**-**	**-**	** + **	**-**	**-**
63	S Hosp, G Bottoni, D Heinrich, P Kofler, M Hasler and W Nachbauer [121]	5	**-**	**-**	** + **	** + **	** + **	** + **	**-**	** + **	** + **	**-**	**-**
64	GG Zanca, SM Mattiello and AR Karduna [133]	6	**-**	**-**	** + **	** + **	** + **	** + **	**-**	**-**	** + **	**-**	**-**
65	DM Hopper, TL Grisbrook, M Finucane and K Nosaka [120]	6	**-**	**-**	** + **	** + **	** + **	** + **	**-**	**-**	** + **	**-**	**-**
66	I Miralles, S Monterde, O del Rio, S Valero, S Montull and I Salvat [61]	6	**-**	**-**	** + **	**-**	** + **	** + **	** + **	**-**	** + **	**-**	**-**
67	M Barbanera, FdA Mazuchi, JPB Batista, JdM Ultremare, JdS Iwashita and UF Ervilha [144]	5	**-**	**-**	** + **	**-**	** + **	** + **	**-**	** + **	** + **	** + **	**-**
68	G Fratocchi, F Di Mattia, R Rossi, M Mangone, V Santilli and M Paoloni [118]	5	**-**	**-**	** + **	** + **	** + **	** + **	**-**	** + **	** + **	**-**	**-**
69	H Niknam, A Sarmadi, M Salavati and F Madadi [139]	2	**-**	** + **	** + **	** + **	** + **	** + **	** + **	** + **	** + **	** + **	**-**
70	Jj Lin, CJ Hung and PL Yang [125]	5	**-**	**-**	** + **	** + **	** + **	** + **	**-**	** + **	** + **	**-**	**-**
71	A Aytar, N Ozunlu, O Surenkok, G Baltacı, P Oztop and M Karatas [100]	8	**-**	**-**	** + **	** + **	**-**	** + **	**-**	**-**	**-**	**-**	**-**
72	W–H Lee, O-Y Kwon, C-H Yi, H–S Jeon and S-M Ha [145]	3	**-**	**-**	** + **	** + **	** + **	** + **	** + **	** + **	** + **	** + **	**-**
73	M Iris, S Monterde, M Salvador, I Salvat, J Fernández-Ballart and B Judith [105]	7	**-**	**-**	** + **	**-**	** + **	** + **	**-**	**-**	** + **	**-**	**-**
74	T Bradley, C Baldwick, D Fischer and GAC Murrell [117]	7	**-**	**-**	** + **	** + **	**-**	** + **	**-**	** + **	**-**	**-**	**-**
75	KM Refshauge, J Raymond, SL Kilbreath, L Pengel and I Heijnen [128]	4	**-**	**-**	** + **	** + **	** + **	** + **	** + **	** + **	** + **	**-**	**-**
76	MJ Callaghan, J Selfe, A McHenry and JA Oldham [53]	4	**-**	**-**	** + **	** + **	** + **	** + **	**-**	** + **	** + **	**-**	**-**
77	S Spanos, M Brunswic and E Billis [143]	5	**-**	** + **	** + **	** + **	** + **	** + **	**-**	**-**	** + **	**-**	**-**
78	H Mokhtarinia, TI Ebrahimi, M Salavati, S Goharpai and A Khosravi [146]	4	**-**	**-**	** + **	** + **	** + **	** + **	**-**	** + **	** + **	** + **	**-**
79	RS Hinman, KM Crossley, J McConnell and KL Bennell [80] A	3	**-**	** + **	** + **	** + **	** + **	** + **	**-**	** + **	** + **	** + **	**-**
	RS Hinman, KM Crossley, J McConnell and KL Bennell [80] B	8	**-**	**-**	**-**	**-**	**-**	** + **	** + **	**-**	** + **	**-**	**-**
80	T Halseth, WM John and D Mark [137]	4	**-**	** + **	** + **	** + **	** + **	** + **	**-**	** + **	** + **	**-**	**-**
81	K Mumford [62]	6	-	**-**	** + **	**-**	** + **	** + **	**-**	**-**	** + **	** + **	**-**
82	AL Cecchinato [54]	6	**-**	**-**	** + **	** + **	** + **	** + **	**-**	**-**	** + **	**-**	**-**
83	MJ Callaghan, J Selfe, PJ Bagley and JA Oldham [55]	5	**-**	**-**	** + **	** + **	** + **	** + **	**-**	** + **	** + **	**-**	**-**
84	TJ Hubbard and TW Kaminski [122]	7	**-**	**-**	** + **	** + **	**-**	** + **	**-**	** + **	**-**	**-**	**-**
85	TW Kaminski and TM Gerlach [123]	5	**-**	**-**	** + **	** + **	** + **	** + **	**-**	** + **	** + **	**-**	**-**
86	KM Refshauge, SL Kilbreath and J Raymond [56]	4	**-**	**-**	** + **	** + **	** + **	** + **	** + **	** + **	** + **	**-**	**-**
87	GG Simoneau, RM Degner, CA Kramper and KH Kittleson [130]	4	**-**	**-**	** + **	** + **	** + **	** + **	** + **	** + **	** + **	**-**	**-**
88	EJ Heit, SM Lephart and SL Rozzi [32]	3	**-**	**-**	** + **	** + **	** + **	** + **	** + **	** + **	** + **	** + **	**-**
89	J Jerosch, I Hoffstetter, H Bork and M Bischof [29]	5	**-**	**-**	** + **	** + **	** + **	** + **	**-**	** + **	** + **	**-**	**-**
90	S Robbins, E Waked and R Rappel [129]	6	**-**	**-**	** + **	** + **	** + **	** + **	**-**	**-**	** + **	**-**	**-**
91	M Schenker [147]	4	**-**	**-**	** + **	** + **	** + **	** + **	** + **	**-**	** + **	** + **	**-**

### Publication bias

**Fig. 3:**
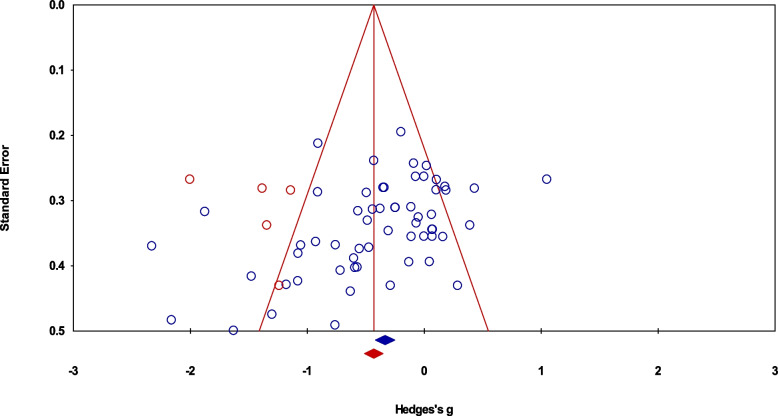
A trim and fill funnel plot illustrating the publication bias. Each study is represented by an individual blue circle, whereas a unique red circle represents the imputed studies. The funnel plot area covers 95% of the pseudo-confidence intervals. The vertical midline represents the estimated overall effect size (i.e., empirical studies + imputed studies)

The incidence of publication bias according to Duval and Tweedie’s trim and fill procedure for the primary outcome of absolute error during joint position sense has been demonstrated in Fig. 3. The method identified five missing studies on the left side of the mean effect, whereas no study was missing on the right side. In the analysis, under the random effect model, the point estimate and the 95% C.I. for the combined studies was -0.39 (-0.54 to -0.24). Using the trim and fill procedure, the imputed point estimates were -0.48 (-0.64 to -0.32).

### Systematic review report

#### Participants

From the 91 included studies, data from a total of 2718 (1043F, 1123M) people was reported. Fourteen of the included studies did not report the sex distribution of their sample [36, 50, 55, 62, 66, 76, 82, 102, 110, 118, 119, 134, 147, 154]. Likewise, seven studies did not report the age description [50, 52, 60, 65, 114, 142, 154]. In the rest of the 91 studies, the average of the sample was 29.7 ± 12.8 years.

In the entire study sample of 2718 individuals, 2166 (812F, 938M) individuals had received the taping. The discrepancy in the sex distribution is because 14 studies, as mentioned before, had failed to report sex distribution in their respective studies. The average age of the sample receiving taping was 29.4 ± 13 years. Additionally, 13 studies compared the taping intervention's efficacy with a placebo taping [57, 75–77, 85, 100–103, 105, 111–113, 116, 118]. Here, data were reported for a total of 279 (115F, 100M) individuals. The discrepancy in the sex distribution was again because three studies did not mention the sex distribution of their participants [76, 102, 118]. The average age in this sample was 35.7 ± 16.6 years. Likewise, 17 studies performed a comparative evaluation by comparing the effectiveness of taping in a group that was subjected to no intervention [54, 55, 60, 67, 74, 78, 82, 101, 104–107, 110, 114, 115, 117, 134]. Here, data were reported from a total of 273 (116F, 85M) individuals. Five studies had not reported their sex distribution [55, 60, 82, 110, 134], and two studies had not reported the age descriptive of the group that did not receive taping [60, 114]. The average age in this sample was 26.1 ± 3.7 years.

#### Health status

Table 2 shows a detailed description of the health status of the participants included in this review.

**Table 2:** Details of participants with different health statuses included in this review

Health status classification	Additional subgroup information	Studies; references	Sample size (Female, Male)	Age	Studies not reporting descriptive; references
Healthy	-	48; [29, 32, 42, 44–46, 49, 50, 52, 55, 56, 59–61, 68, 70, 75, 77, 102, 104–106, 112–115, 117–120, 123, 125–127, 129–132, 137, 140–142, 144–146, 149]	1293 (471F, 500M)	25.2 ± 8.2	15; [29, 44, 46, 49, 56, 59, 70, 105, 113, 114, 129, 137, 142, 145, 149]
Fatigue	Healthy	9; [48, 49, 52, 74, 96, 121, 131, 133, 140]	243 (134F, 92M)	24 ± 4.4	1; [49]
	Functional ankle instability	1; [54]	28 (?)	-	1; [54]
Ankle instability	-	1; [29]	16 (?)	23.9 ± 2.8	1; [29]
	Functional ankle instability	7; [54, 76, 101, 122, 124, 135, 138]	158 (62F, 49M)	26.3 ± 3.3	2; [54, 76]
	Chronic ankle instability	4; [45, 46, 73, 98]	156 (35F, 84M)	28.4 ± 10.6	1; [46]
Ankle sprain	-	2; [128, 147]	80 (52F, 28M)	20.4 ± 3	-
	Ankle inversion sprain	2; [56, 143]	38 (4F, 16M)	23.5 ± 0.5	1; [56]
	Chronic inversion injury	1; [62]	40 (16F, 4M)	23.3 ± 2.6	-
Osteoarthritis	-	4; [47, 79, 80, 103]	187 (93F, 94M)	60.9 ± 4.8	-
Patellofemoral pain syndrome	-	4; [53, 100, 107, 146]	113 (51F, 62M)	27.3 ± 4.3	-
Anterior cruciate ligament	Rupture	3; [72, 81, 82]	112 (9F, 103M)	27.5 ± 3.8	-
	Reconstruction	2; [134, 139]	40 (40F)	25.7 ± 1.8	-
Stroke	-	3; [58, 71, 78]	41 (23F, 38M)	53.1 ± 5.4	-
Shoulder impingement syndrome	-	5; [51, 106, 109, 111, 148]	68 (39F, 29M)	37.7 ± 15.1	1; [109]
Dynamic knee valgus	-	2; [69, 110]	43 (38F, 5M)	31.4 ± 16.8	-
Scapular asymmetry	-	1; [149]	16 (?)	?	1; [149]
Mechanical neck pain	-	1; [97]	66 (?)	22.9 ± 0.2	1; [97]
Sacroiliac joint dysfunction	-	1; [99]	30 (30F)	36. [103]7 ± 0.7	-
Chronic low back pain	-	1; [95]	30 (15F, 15M)	43.2 ± 1.5	-
Knee pain	-	1; [113]	22 (?)	70.1 ± 2.3	1; [113]
Medial tibial stress syndrome	-	1; [108]	16 (16M)	26.2 ± 3.3	-
Lateral epicondylitis		1; [145]	15 (?)	41.9 ± 6.8	1; [145]

### Type of tape

Fourteen different types of tapes were utilized in the included studies (Table S2). The tapes were classified as either rigid or elastic tapes based on the description provided in the respective studies. Overall, sixty studies had utilized elastic tapes [48, 50–54, 56–58, 65–67, 74–79, 81, 82, 84, 85, 87, 88, 90, 100–102, 105–108, 111, 113, 114, 116–118, 120, 123, 124, 126, 129–132, 137–147, 153, 154], and 33 studies had used rigid tapes [36, 39, 55, 59–62, 64, 80, 83, 86, 103, 104, 110, 115, 119, 122, 125, 127, 128, 133–136, 148–152]. Additionally, four studies had compared the efficacy across two different tapes. Here, two studies had compared the efficacy between elastic and rigid tapes [90, 109], whereas one study each had evaluated the efficacy between two different types of elastic [56], and rigid tapes [68].

### Proprioceptive assessment

Seven different types of proprioceptive assessments were used in the included studies to evaluate proprioceptive performance. Here, 71 of the included studies had used joint sense tests [36, 39, 48, 50, 54–61, 65–68, 74–78, 81–88, 100–107, 109, 110, 112–116, 118, 119, 122, 124–126, 128, 129, 131, 132, 134, 136–140, 142–150, 152, 153], four studies had used threshold to detect of passive motion test [62, 111, 127, 133], four had used active movement extent discrimination accuracy test [51, 52, 80, 90], one had used proprioceptive feedback index (i.e., derived from repositioning error and the correlation between instant movement and prototype instant movement) [130], one study had used active displacement test [154], one study had added proprioceptive test accuracy scores (i.e., moving target program on an isokinetic dynamometer) [120], one had used proprioceptive index (i.e., x, y, rotation values) [141], and one had evaluated the percentage of exact joint position sense trial [123]. Additionally, seven studies had performed assessment of joint position sense as well as threshold to detection of passive motion [53, 64, 79, 108, 117, 135, 151].

### Outcome

The outcomes of individual studies, categorized by the type of proprioceptive assessment used, are summarized as follows:

1. Joint position sense: Among the 78 studies assessing the impact of taping on joint position sense 47 reported a significant improvement in repositioning accuracy [36, 39, 48, 50, 53, 54, 56, 64, 75–79, 83–85, 87, 88, 102, 103, 106–108, 110, 112–116, 119, 124, 132, 134–136, 139, 140, 143, 144, 146–148, 150, 151, 153], 27 reported no difference [55, 57, 59–61, 65, 67, 81, 82, 86, 100, 101, 104, 105, 109, 117, 118, 122, 125, 126, 128, 129, 131, 137, 138, 142, 145, 149, 152], and four reported a significant decline in repositioning accuracy with taping [58, 66, 68, 74].
2. Threshold to detection of passive motion: In the 11 studies examining the effect of taping on the threshold to detection of passive motion, two studies observed a significant improvement [64, 117], one study reported significant deterioration [133], while eight studies found no significant impact of taping [53, 61, 62, 108, 111, 121, 127, 151].
3. Active movement extent discrimination apparatus: Three studies indicated a significant improvement in active movement extent discrimination [51, 52, 80], while one study found no difference in discrimination capabilities with taping [90].
4. Active displacement test: One study assessing the influence of taping on active displacement outcomes reported a significant improvement in displacement capabilities with taping [154].
5. Percentage of exact joint position sense trials: In one study, no significant effect of taping on the ability to perform exact joint repositioning trials was reported [123].
6. Proprioceptive feedback index: A study evaluating the impact of taping on the proprioceptive feedback index reported a significant improvement with taping.
7. Proprioceptive index: One study assessing the influence of taping on the proprioceptive index reported a significant improvement with taping [141].
8. Proprioceptive test accuracy: One study reported no significant effect of taping on proprioceptive test accuracy trials [120].

### Meta-analysis report

Table 3 provides comprehensive insights into the meta-analysis results for absolute repositioning error, threshold to detection of passive motion, and discrimination of active movement extent apparatus. It offers a thorough examination of the between-group analysis comparing taping, placebo, and no comparators. Similarly, Table 4 illustrates the outcomes of the meta-analysis within each group.

**Table 3:** Between-group meta-analysis outcomes for repositioning error

**No**	**Outcome**	**Number of studies; (References)**	**Meta-analysis outcome** Hedge’s g, 95% Confidence interval, *p*-value	**Heterogeneity** I^2^	**Figure**
**Absolute repositioning Error (comparator: No taping)**					
1	Overall	48; [29, 32, 42, 44, 48–50, 52–55, 60–62, 68, 72, 73, 75, 76, 80, 82, 96, 99, 101, 102, 105, 109, 110, 112, 117, 119–121, 123–127, 129–133, 144–148]	-0.39 (-0.54 to -0.24), *p* < 0.001	70%	4
**Study design**					
2	Randomized designs	38; [29, 42, 48–50, 53–55, 60, 61, 68, 72, 73, 75, 76, 80, 96, 99, 101, 102, 105, 109, 110, 112, 117, 119–121, 123–127, 129–133]	-0.39 (-0.57 to -0.21), *p* < 0.001	71%	S1
3	Non-randomized designs	11; [32, 44, 52, 62, 80, 82, 144–148]	-0.40 (-0.69 to -0.10), *p* = 0.009	68%	S2
**Tape type**					
4	Elastic tape	27; [42, 44, 48, 50, 52, 60, 61, 68, 72, 73, 75, 76, 82, 96, 101, 102, 109, 112, 119, 121, 124–127, 132, 133, 148]	-0.41 (-0.67 to -0.15), *p* = 0.002	79%	S3
5	Rigid tape	21; [29, 32, 49, 53–55, 62, 80, 99, 105, 110, 117, 120, 123, 129–131, 144–147]	-0.37 (-0.53 to -0.20), *p* < 0.001	44%	S4
**Population group**					
6	Healthy	32; [29, 32, 42, 44, 49, 50, 52, 55, 60, 61, 68, 75, 102, 105, 110, 112, 117, 119–121, 123, 125–127, 129–133, 144–146]	-0.29 (-0.47 to -0.11), *p* = 0.001	8.34%	S5
7	Healthy: fatigue	8; [48, 49, 52, 96, 121, 129, 131, 133]	-0.31 (-0.71 to 0.09), *p* = 0.12	4.6%	
8	Functional ankle instability	3; [54, 76, 101]	-0.66 (-2.29 to 0.97), *p* = 0.42	0.5%	
9	ACL rupture	2; [72, 82]	-0.66 (-1.24 to -0.09), *p* = 0.02	0%	
10	Shoulder impingement syndrome	2; [109, 148]	-0.56 (-1.51 to 0.38), *p* = 0.24	0%	
11	Functional ankle instability: fatigue	2; [54, 124]	-0.24 (-0.74 to 0.26), *p* = 0.35	0%	
12	PFPS	2; [53, 146]	-0.14 (-0.50 to 0.22), *p* = 0.45	0%	
13	Osteoarthritis	1; [80]	-0.02 (-0.42 to 0.38), *p* = 0.92	0%	
14	Chronic ankle instability	1; [73]	-		
15	Lateral epicondylitis	1; [145]	-		
16	Ankle instability	1; [29]	-		
17	Chronic ankle inversion injury	1; [62]	-		
18	Ankle sprain	1; [147]	-		
19	Sacroiliac joint dysfunction	1; [99]	-		
**Population group: Elastic tape**					
20	Healthy	17; [42, 44, 50, 52, 60, 61, 68, 75, 102, 112, 119, 121, 125–127, 132, 133]	-0.30 (-0.62 to 0.006), *p* = 0.055	10.3%	S6
21	Healthy: fatigue	5; [48, 52, 96, 121, 133]	-0.08 (-0.47 to 0.30), *p* = 0.67	0%	
22	ACL rupture	2; [72, 82]	-0.66 (-1.24 to -0.09), *p* = 0.02	0%	
23	Functional ankle instability	2; [76, 101]	-1.12 (-3.46 to 1.22), *p* = 0.34	0%	
24	Shoulder impingement syndrome	2; [109, 148]	-0.56 (-1.51 to 0.38), *p* = 0.24	0%	
25	Functional ankle instability: fatigue	1; [124]	-		
26	Chronic ankle instability	1; [73]	-		
**Population group: Rigid tape**					
27	Healthy	15; [29, 32, 49, 55, 105, 117, 120, 123, 129–131, 144–146]	-0.29 (-0.46 to -0.12), *p* = 0.001	1.3%	S7
28	Healthy: fatigue	3; [49, 129, 131]	-0.72 (-1.54 to 0.09), *p* = 0.082	0%	
29	Osteoarthritis	1; [80]	-0.02 (-0.42 to 0.38), *p* = 0.92	0%	
30	PFPS	2; [53, 146]	-0.14 (-0.50 to 0.22), *p* = 0.45	0%	
31	Chronic ankle inversion injury	1; [62]	-		
32	Ankle sprain	1; [147]	-		
33	Sacroiliac joint dysfunction	1; [99]	-		
34	Functional ankle instability	1; [54]	-		
35	Functional ankle instability: fatigue	1; [54]	-		
36	Lateral epicondylitis	1; [145]	-		
37	Ankle instability	1; [29]	-		
**Absolute repositioning Error (comparator: Placebo taping)**					
38	Overall	25; [42, 51, 59, 69–71, 73, 75, 78–80, 95–98, 100, 107, 108, 111, 113, 119, 124, 127, 132, 133]	-1.20 (-1.68 to -0.70), *p* < 0.001	93%	S8
**Study design**					
39	Randomized designs	24; [42, 51, 69–71, 73, 75, 78–80, 95–98, 100, 107, 108, 111, 113, 119, 124, 127, 132, 133]	-1.27 (-1.80 to -0.74), *p* < 0.001	93%	S9
40	Non-randomized designs	2; [59, 80]	-0.20 (-0.69 to 0.29), *p* = 0.42	0%	S10
**Tape type**					
41	Elastic tape	23; [42, 51, 59, 69–71, 73, 75, 78, 79, 95–97, 100, 107, 108, 111, 113, 119, 124, 127, 132, 133]	-1.13 (-1.65 to -0.60), *p* < 0.001	93%	S11
42	Rigid tape	2; [80, 98]	-1.67 (-3.48 to 0.13), *p* = 0.07	96%	S12
**Population group**					
43	Healthy	9; [42, 59, 70, 75, 98, 119, 127, 132, 133]	-0.61 (-1.09 to -2.46), *p* = 0.01	28.5%	S13
44	Osteoarthritis	2; [75, 80]	-2.21 (-4.26 to -0.16), *p* = 0.03	0%	
45	Healthy: fatigue	2; [96, 133]	-0.31 (-0.90 to 0.26), *p* = 0.28	0%	
46	PFPS	2; [100, 107]	-4.18 (-11.53 to 3.16), *p* = 0.26	0%	
47	Stroke	2; [71, 78]	-0.18 (-2.02 to 1.65), *p* = 0.84	0%	
48	Shoulder impingement syndrome	2; [51, 111]	-0.43 (-1.81 to 0.94), *p* = 0.54	0%	
49	Functional ankle instability: fatigue	1; [124]	-		
50	Mechanical neck pain	1; [97]	-		
51	Chronic ankle instability	1; [73]	-		
52	Chronic low back pain	1; [95]	-		
53	Dynamic knee valgus	1; [69]	-		
54	Knee pain	1; [113]	-		
55	MTSS	1; [108]	-		
**Population group: Elastic tape**					
56	Healthy	8; [42, 59, 70, 75, 119, 127, 132, 133]	-0.58 (-1.14 to -0.03), *p* = 0.039	28%	S14
58	Healthy: fatigue	2; [96, 133]	-0.31 (-0.89 to 0.26), *p* = 0.28	0%	
59	PFPS	2; [100, 107]	-4.18 (-11.53 to 3.16), *p* = 0.26	0%	
60	Shoulder impingement syndrome	2; [51, 111]	-0.43 (-1.81 to 0.94), *p* = 0.54	0%	
61	Stroke	2; [71, 78]	-0.18 (-2.02 to 1.65), *p* = 0.84	0%	
62	Chronic ankle instability	1; [73]	-		
63	Chronic low back pain	1; [95]	-		
64	Dynamic knee valgus	1; [69]	-		
65	Knee pain	1; [113]	-		
66	MTSS	1; [108]	-		
67	Osteoarthritis	1; [79]	-		
68	Mechanical neck pain	1; [97]	-		
69	Functional ankle instability: fatigue	1; [124]	-		
**Population group: Rigid tape**					
70	Osteoarthritis	1; [80]	-		
71	Healthy	1; [98]	-		
**Threshold to detection of passive motion (comparator: no tape)**					
72	Overall	8; [55, 56, 73, 112, 122, 128, 130, 146]	-0.017 (-0.18 to 0.15), *p* = 0.84	0%	S15
**Study design**					
73	Randomized designs	Same as outcome 72			
74	Non-randomized designs	-			
**Tape type**					
75	Elastic	2; [73, 112]	-0.26 (-0.66 to 0.13), *p* = 0.19	0%	S16
76	Rigid	6; [55, 56, 122, 128, 130, 146]	0.04 (-0.15 to 0.23), *p* = 0.68	0%	
**Population groups**					
77	Healthy	6; [55, 56, 112, 122, 130, 146]	0.004 (-0.21 to 0.22), *p* = 0.97	0%	S17
78	Ankle sprain	2; [56, 128]	0.06 (-0.55 to 0.67), *p* = 0.85	0%	
79	Chronic ankle instability	1; [73]	-	-	-
80	Patellofemoral pain syndrome	1; [146]	-	-	-
81	Functional ankle instability	1; [122]	-	-	-
**Population groups: Elastic tape**					
82	Healthy	1; [112]	-	-	-
83	Chronic ankle instability	1; [73]	-	-	-
**Population groups: Rigid tape**					
84	Healthy	5; [55, 56, 122, 130, 146]	0.06 (-0.55 to 0.67), *p* = 0.85	0%	S18
85	Ankle sprain	2; [56, 128]	0.05 (-0.18 to 0.29), *p* = 0.68	0%	
86	Patellofemoral pain syndrome	1; [146]	-		
87	Functional ankle instability	1; [122]	-		
**Threshold to detection of passive motion (comparator: placebo tape)**					
88	Overall	2; [73, 106]	-1.35 (-3.58 to 0.87), *p* = 0.23	0%	S19
**Study design**					
89	Randomized designs	Same as outcome number 88			
90	Non-randomized designs	-	-	-	-
**Tape type**					
91	Elastic	Same as outcome number 88			
92	Rigid	-	-	-	-
**Population type**					
93	Chronic ankle instability	1; [73]	-	-	-
94	Shoulder impingement syndrome	1; [106]	-	-	-
**Population groups: Elastic tape**					
95	Chronic ankle instability	1; [73]	-	-	-
96	Shoulder impingement syndrome	1; [106]	-	-	-
**Population groups: Rigid tape**					
97	None	-	-	-	-
**Active movement extent discrimination apparatus (comparator: no tape)**					
98	Overall	2; [45, 84]	0.17 (-0.37 to 0.72), *p* = 0.54	67%	S20
**Study design**					
99	Randomized designs	1; [45]	-	-	-
100	Non-randomized designs	1; [84]	-	-	-
**Tape type**					
101	Elastic	2; [45, 84]	0.39 (-0.07 to 0.85), *p* = 0.09	33%	S21
102	Rigid	1; [84]	-	-	-
**Population type**					
103	Healthy	2; [45, 84]	0.39 (-0.34 to 0.90), *p* = 0.37	0%	S22
104	Chronic ankle instability	1; [45]	-	-	-
**Population type: Elastic tape**					
105	Healthy	2; [45, 84]	0.28 (-0.34 to 0.90), *p* = 0.37	0%	S23
106	Chronic ankle instability	1; [45]	-	-	-
**Population type: Rigid tape**					
107	Healthy	1; [84]	-	-	-
**Active movement extent discrimination apparatus (comparator: placebo tape)**					
108	Overall	-	-	-	-

*ACL* Anterior cruciate ligament, *MTSS* Medial tibial stress syndrome, *PFPS* Patellofemoral pain syndrome, *S* Supplementary figure

**Fig. 4:**
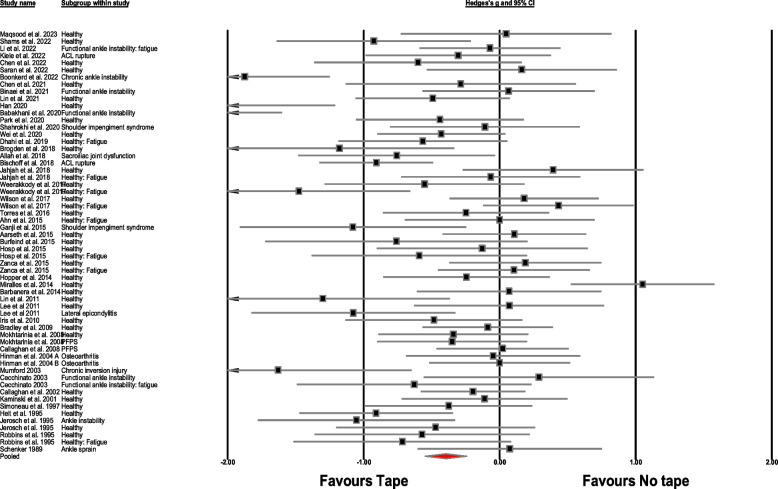
Forest plot illustrating the between group effect of taping on repositioning error. Black boxes: individual weighted effect sizes (Hedge's g), whiskers: 95% confidence intervals, red diamond: pooled weighted effect size and 95% CI, positive effect size: reduced repositioning error for the placebo/no-taping group, negative effect size: reduced repositioning error for the taping group

**Table 4:** Within-group meta-analysis outcomes for repositioning error

**No**	**Outcome**	**Number of studies; (References)**	**Meta-analysis outcome** Hedge’s g, 95% Confidence interval, *p*-value	**Heterogeneity** I^2^	**Figure**
**Absolute repositioning Error**					
109	Overall	45; [47, 54, 58, 59, 61, 62, 68–72, 77–81, 95–105, 107–114, 133–135, 137–143]	-0.65 (-0.91 to -0.38), *p* < 0.001	88%	S24
**Study design**					
**110**	Randomized design	31; [54, 61, 68–72, 77–80, 95–105, 107–114, 133]	-0.71 (-1.04 to -0.38), *p* < 0.001	88%	S25
**111**	Non-randomized design	15; [47, 58, 59, 62, 80, 81, 134, 135, 137–143]	-0.51 (-0.98 to -0.05), *p* = 0.02	87%	S26
**Tape type**					
112	Elastic tape	34; [47, 59, 61, 68–72, 78, 79, 81, 95–97, 100–104, 107–109, 111–113, 133–135, 137–142]	-0.63 (-0.95 to -0.32), *p* < 0.001	49.5%	S27
113	Rigid tape	11; [54, 58, 62, 77, 80, 98, 99, 105, 110, 114, 143]	-0.70 (-1.12 to -0.20), *p* = 0.006	0%	
**Population type**					
114	Healthy	16; [59, 61, 68, 70, 77, 98, 102, 104, 105, 112, 114, 133, 137, 140–142]	-0.55 (-0.97 to -0.13), *p* = 0.10	0%	S28
115	Osteoarthritis	4; [47, 79, 80, 103]	-0.71 (-1.62 to 0.19), *p* = 0.12	29.7%	
116	Functional ankle instability	4; [54, 101, 135, 138]	-0.45 (-1.9 to 0.99), *p* = 0.53	14.3%	
117	Healthy: Fatigue	3; [96, 133, 140]	0.58 (-0.35 to 1.51), *p* = 0.22	0%	
118	Stroke	3; [58, 71, 78]	-0.74 (-1.93 to 0.44), *p* = 0.21	0%	
119	ACL reconstruction	2; [134, 139]	-5.55 (-12.7 to 1.62), *p* = 0.13	0%	
120	ACL rupture	2; [72, 81]	-0.75 (-1.11 to -0.39); *p* < 0.001	0%	
121	Ankle sprain	2; [62, 143]	-0.62 (-1.12 to -0.11), *p* = 0.01	0%	
122	Dynamic knee valgus	2; [69, 110]	-1.42 (-1.99 to -0.85), *p* < 0.001	0%	
123	PFPS	2; [100, 107]	-0.27 (-0.60 to 0.06), *p* = 0.11	0%	
124	Shoulder impingement syndrome	2; [109, 111]	-0.26 (-0.76 to 0.23), *p* = 0.29	0%	
125	Mechanical neck pain	1; [97]	-	-	-
126	Knee pain	1; [113]	-	-	-
127	Sacroiliac joint dysfunction	1; [99]	-	-	-
128	Chronic low back pain	1; [95]	-	-	-
129	Functional ankle instability: Fatigue	1; [54]	-	-	-
130	MTSS	1; [108]	-	-	-
**Population type: Elastic tape**					
131	Healthy	12; [59, 61, 68, 70, 102, 104, 112, 133, 140–142]	-0.18 (-0.42 to 0.06), *p* = 0.14	0%	S29
132	Functional ankle instability	3; [101, 135, 138]	-0.67 (-2.57 to 1.23), *p* = 0.49	19.5%	
133	Healthy fatigue	3; [96, 133, 140]	0.58 (.0.35 to 1.51), *p* = 0.22	0%	
134	Osteoarthritis	3; [47, 79, 103]	-1.15 (-2.82 to 0.51), *p* = 0.17	11.4%	
135	ACL reconstruction	2; [134, 139]	-5.55 (-12.73 to 1.62), *p* = 0.13	0%	
136	ACL rupture	2; [72, 81]	-0.75 (-1.11 to -0.39), *p* < 0.001	0%	
137	Shoulder impingement syndrome	2; [109, 111]	-0.26 (-0.76 to 0.22), *p* = 0.29	0%	
138	Stroke	2; [71, 78]	-1.14 (-2.61 to 0.23), *p* = 0.29	0%	
139	PFPS	2; [100, 107]	-0.27 (-0.60 to 0.06), *p* = 0.11	0%	
140	Chronic low back pain	1; [95]	-	-	-
141	Dynamic knee valgus	1; [69]	-	-	-
142	Knee pain	1; [113]	-	-	-
143	Mechanical neck pain	1; [97]	-	-	-
144	MTSS	1; [108]	-	-	-
**Population type: Rigid tape**					
145	Healthy	4; [77, 98, 105, 114]	-1.52 (-2.45 to -0.59), *p* = 0.001	14.4%	S30
146	Osteoarthritis	1; [80]	-0.11 (-0.51 to 0.29), *p* = 0.58	0%	
147	Ankle sprain	2; [62, 143]	-0.62 (-1.12 to -0.11), *p* = 0.01	0%	
148	Dynamic knee valgus	1; [110]	-	-	-
149	Functional ankle instability	1; [54]	-	-	-
150	Functional ankle instability: Fatigue	1; [54]	-	-	-
151	Sacroiliac joint dysfunction	1; [99]	-	-	-
152	Stroke	1; [58]	-	-	-
**Threshold to detection of passive motion**					
153	Overall	5; [47, 58, 103, 106, 112]	-0.36 (-0.66 to -0.07), *p* = 0.01	0%	S31
**Study design**					
154	Randomized designs	3; [103, 106, 112]	-0.39 (-0.76 to -0.02), *p* = 0.03	17%	S32
155	Non-randomized designs	2; [47, 58]	-0.25 (-0.91 to 0.40), *p* = 0.44	0%	S33
156	Elastic tape	4; [47, 103, 106, 112]	-0.38 (-0.69 to -0.06), *p* = 0.018	0%	S34
157	Rigid tape	1; [58]	-	-	-
**Population type**					
158	Healthy	2 [106, 112];	-0.45 (-1.37 to 0.47), *p* = 0.33	0%	S35
159	Osteoarthritis	2 [47, 103];	-0.28 (-0.73 to 0.17), *p* = 0.22	0%	
160	Stroke	1 [58];	-	-	-
161	Shoulder impingement syndrome	1 [106];	-	-	-
**Population type: Elastic tape**					
162	Healthy	Same as outcome number 158			
163	Osteoarthritis	Same as outcome number 159			
164	Shoulder impingement syndrome	1 [106];	-	-	-
**Population type: Rigid tape**					
165	Stroke	1; [58]	**-**	**-**	**-**
**Active movement extent discrimination apparatus**					
166	Overall	1; [74]	**-**	**-**	**-**

*ACL* Anterior cruciate ligament, *MTSS* Medial tibial stress syndrome, *PFPS* Patellofemoral pain syndrome, *S* Supplementary figure

### Sensitivity analysis

A summary of the leave-one-out sensitivity analysis has been provided in Table 5. Specifically, studies were reported if the overall analysis yielded a *p*-value less than 0.05, and the removal of a specific study increased the *p*-value above this threshold. Conversely, studies were also reported if the overall analysis yielded a *p*-value greater than 0.05 and the removal of any particular study decreased the *p*-value below this threshold.

**Table 5:** Leave one out sensitivity analysis

No	Analysis	Meta-analysis *p*-value	**I**^**2**^	Studies impacting the *p*-value upon removal	***p*-value upon removal**
**Between group, absolute repositioning error: no taping comparator**					
1	Overall	< 0.001	70%	-	No effect
2	Randomized design	< 0.001	71%	-	No effect
3	Non-randomized design	0.009	68%	-	No effect
4	Elastic tape	0.002	79%	-	No effect
5	Rigid tape	< 0.001	79%	-	No effect
6	Healthy	0.001	8.3%		No effect
7	Healthy: fatigue	0.12	4.6%	-	No effect
8	Functional ankle instability	0.42	0.5%	F Binaei, R Hedayati, M Mirmohammadkhani, C Taghizadeh Delkhoush and R Bagheri [76] AL Cecchinato [54]	0.01 0.01
9	ACL rupture	0.02	0%	-	No effect
10	Shoulder impingement syndrome	0.24	84%	-	No effect
11	Functional ankle instability: fatigue	0.35	0%	-	No effect
12	PFPS	0.45	0%	-	No effect
13	Osteoarthritis	0.92	0%	-	No effect
14	Healthy (elastic tape)	0.05	10.3%	-	No effect
15	Healthy: fatigue (elastic tape)	0.67	0%	-	No effect
16	ACL rupture (elastic tape)	0.02	0%	-	No effect
17	Functional ankle instability	0.34	0%	-	No effect
18	Shoulder impingement syndrome	0.24	0%	-	No effect
19	Healthy (rigid tape)	0.001	1.3%	-	No effect
20	Healthy: fatigue (rigid tape)	0.082	0%	-	No effect
21	Osteoarthritis (rigid tape)	0.92	0%	-	No effect
22	PFPS (rigid tape)	0.45	0%	-	No effect
**Between group, absolute repositioning error: placebo comparator**					
23	Overall	< 0.001	93%	-	No effect
24	Randomized designs	< 0.001	93%	-	No effect
25	Non-randomized designs	0.42	0%	-	No effect
26	Elastic tape	< 0.001	93%	-	No effect
27	Rigid tape	0.07	96%	RS Hinman, KM Crossley, J McConnell and KL Bennell [80] osteoarthritis population M Alawna and AA Mohamed [98]	0.12 0.23
28	Healthy	0.01	28.5%	-	No effect
29	Osteoarthritis	0.03	0%	-	No effect
30	Healthy: fatigue	0.28	0%	IK Ahn, YL Kim, Y-H Bae and SM Lee [96]	0.04
31	PFPS	0.26	0%	A Aytar, N Ozunlu, O Surenkok, G Baltacı, P Oztop and M Karatas [100]	< 0.001
32	Stroke	0.84	0%	GLd Santos, MB Souza, K Desloovere and TL Russo [78]	0.001
33	Shoulder impingement syndrome	0.54	0%	HE Göktaş, S Çitaker and ED Yurtsever [51]	0.003
34	Healthy (elastic tape)	0.039	28%	J-T Han [119]	0.11
35	Healthy: fatigue (elastic tape)	0.28	0%	IK Ahn, YL Kim, Y-H Bae and SM Lee [96]	0.04
36	PFPS (elastic tape)	0.26	0%	A Aytar, N Ozunlu, O Surenkok, G Baltacı, P Oztop and M Karatas [100]	< 0.001
37	Shoulder impingement syndrome (elastic tape)	0.54	0%	HE Göktaş, S Çitaker and ED Yurtsever [51]	0.003
38	Stroke (elastic tape)	0.84	0%	GLd Santos, MB Souza, K Desloovere and TL Russo [78]	0.001
**Between group, absolute error: placebo comparator**					
24	Overall	0.01	55%	H-Y Chang, K-Y Chou, J-J Lin, C-F Lin and C-H Wang [165]	0.056
25	Repeated measures design*	Same as outcome number 24			
26	Elastic tape	Same as outcome number 24			
27	Rigid tape	-	**-**	-	-
28	Healthy	0.007	56%	H-Y Chang, K-Y Chou, J-J Lin, C-F Lin and C-H Wang [165]	0.089
29	Medial epicondylitis	0.31	0%	-	No effect
30	Functional ankle instability (fatigue)	-	-	-	-
31	Healthy (elastic tape)	Same as outcome number 28			
32	Medial epicondylitis (elastic tape)	Same as outcome number 29			
33	Functional ankle instability-fatigue (elastic tape)	-	-	-	-
**Between group, threshold to detection of passive motion (comparator: no tape)**					
34	Overall	0.84	0%	-	No effect
35	Elastic tape	0.19	0%	-	No effect
36	Rigid tape	0.68	0%	-	No effect
37	Healthy	0.97	0%	-	No effect
38	Ankle sprain	0.85	0%	-	No effect
39	Healthy (rigid tape)	0.85	0%	-	No effect
40	Ankle sprain (rigid tape)	0.68	0%	-	No effect
**Between group, threshold to detection of passive motion (comparator: placebo tape)**					
41	Overall	0.23	0%	C Boonkerd, K Thinchuangchan, N Chalarak, S Thonpakorb, R Wanasoonthontham, T Kitsuksan and T Laddawong [73]	< 0.001
**Between group, active movement extent discrimination apparatus (comparator: no tape)**					
42	Overall	0.54	67%	-	No effect
43	Elastic tape	0.09	33%	Z Long, R Wang, J Han, G Waddington, R Adams and J Anson [84]	0.01
44	Healthy	0.37	0%	-	No effect
45	Healthy (elastic tape)	0.37	0%	-	No effect
**Within group, absolute repositioning error**					
46	Overall	< 0.001	88%	-	No effect
47	Randomized design	< 0.001	88%	-	No effect
48	Non-randomized design	0.002	87%	-	No effect
49	Elastic tape	< 0.001	49.5%	-	No effect
50	Rigid tape	0.006	0%	-	No effect
51	Healthy	0.10	0%	-	No effect
52	Osteoarthritis	0.12	29.7%	-	No effect
53	Functional ankle instability	0.53	14.3%	-	No effect
54	Healthy: Fatigue	0.22	0%	-	No effect
55	Stroke	0.21	0%	-	No effect
56	ACL reconstruction	0.13	0%	-	No effect
57	ACL rupture	< 0.001	0%	-	No effect
58	Ankle sprain	0.001	0%	S Spanos, M Brunswic and E Billis [143]	0.29
59	Dynamic knee valgus	< 0.001	0%	-	No effect
60	PFPS	0.11	0%	-	No effect
61	Shoulder impingement syndrome	0.29	0%	-	No effect
62	Healthy (elastic tape)	0.14	0%	I Miralles, S Monterde, O del Rio, S Valero, S Montull and I Salvat [61]	0.005
63	Functional ankle instability (elastic tape)	0.49	19.5%	-	No effect
64	Healthy fatigue (elastic tape)	0.22	0%	-	No effect
65	Osteoarthritis (elastic tape)	0.17	11.4%	-	No effect
66	ACL reconstruction (elastic tape)	0.13	0%	-	No effect
67	ACL rupture (elastic tape)	< 0.001	0%	-	No effect
68	Shoulder impingement syndrome (elastic tape)	0.29	0%	-	No effect
69	Stroke (elastic tape)	0.29	0%	GLd Santos, MB Souza, K Desloovere and TL Russo [78]	< 0.001
70	PFPS (elastic tape)	0.11	0%	-	No effect
71	Healthy (rigid tape)	0.001	14.4%	-	No effect
72	Osteoarthritis (rigid tape)	0.58	0%	-	No effect
73	Ankle sprain (rigid tape)	0.01	0%	S Spanos, M Brunswic and E Billis [143]	0.29
**Within group, threshold to detection of passive motion**					
74	Overall	0.01	0%	R Torres, R Trindade and RS Gonçalves [112]	0.19
75	Randomized designs	0.03	17%	R Torres, R Trindade and RS Gonçalves [112] KA Keenan, JS Akins, M Varnell, J Abt, M Lovalekar, S Lephart and TC Sell [106]: subacromial impingement participants F Fazli, A Farsi, IE Takamjani, S Mansour, N Yousefi and F Azadinia [103]	0.28 0.11 0.14
76	Non-randomized designs	0.44	0%	-	No effect
77	Elastic tape	0.02	0%	F Fazli, A Farsi, IE Takamjani, S Mansour, N Yousefi and F Azadinia [103] R Torres, R Trindade and RS Gonçalves [112]	0.061 0.23
78	Healthy	0.33	0%	KA Keenan, JS Akins, M Varnell, J Abt, M Lovalekar, S Lephart and TC Sell [106]: healthy participants	0.007
79	Osteoarthritis	0.22	0%	-	No effect

*ACL* Anterior cruciate ligament, *PFPS* Patellofemoral pain syndrome

## Discussion

This systematic review and meta-analysis aimed to synthesize the current state of knowledge regarding the influence of taping on joint proprioception in healthy and patient population groups. The findings from the review suggest a positive influence of taping on improving joint repositioning accuracy against both placebo and no comparator groups.

To date, only two meta-analyses have quantified the influence of taping on proprioceptive accuracy [71, 72]. In the initial review, five studies reported medium effect enhancements (Hedge’s g: 0.25) in proprioceptive accuracy among individuals with ankle instability [72]. In an additional analysis with two studies, the authors reported trivial deterioration (g: -0.10) in knee proprioception among individuals with patellofemoral pain syndrome. In the second review, the authors included a total of seven studies and reported a positive influence of taping/bracing on joint position sense (0.20º) but not the threshold to movement detection (-0.24º) [71]. However, it is essential to note that the authors merged the outcomes of studies and did not differentiate the results between taping and bracing. This merged reporting of effects could be an essential factor that biases the understanding concerning the overall influence of taping on joint proprioception. The present study, through a review of 91 studies, represents a significant advancement over previous reviews. Firstly, unlike prior studies that merged various joint stabilizers, such as taping and bracing [71], the present study focused solely on taping, allowing for a more precise evaluation of taping’s efficacy. Secondly, deliberate analyses based on the type of assessment ensured distinct between-group and within-group comparisons, a modification absent in prior research. Thirdly, the review extended beyond previous studies by systematically differentiating outcomes according to study design i.e., distinguishing between randomized and non-randomized designs. Fourth, the study explored nuanced variations in taping outcomes across different health statuses, providing valuable insights for clinicians and patients. Fifth, the evaluation of tape elasticity, encompassing both elastic and rigid varieties, sheds light on how different tape properties influence joint proprioception. Overall, these additions enrich the existing literature and expand understanding concerning the taping's impact on proprioception.

In line with the previous findings, a *medium-*to-*large* effect improvement in joint repositioning accuracy was observed with taping in the between group analyses against no comparator (Hedge’s g: -0.39), placebo comparator (g: -1.20) and in the within-group (-0.65) analyses. While the magnitude of improvement for the joint position sense tests was larger for the placebo group compared to the no taping group, it's crucial to note that both of these improvements were statistically significant (*p* < 0.05). This suggests that regardless of the intervention (placebo or no taping), there was a substantial enhancement in repositioning accuracy. Moreover, the analysis revealed a notable difference in the number of studies included, with 48 studies in the no taping analysis compared to 25 studies in the placebo analysis. This variance in the number of studies might have influenced the observed difference in magnitude [155]. For instance, a larger pool of studies in the no taping analysis could potentially dilute the effect size, whereas a smaller number of studies in the placebo analysis might result in a more pronounced effect size. Therefore, despite the varying magnitudes of improvement, the consistent statistical significance across both groups underscores the importance of the observed enhancement in joint position sense accuracy. These effects were also visible in subsequent subgroup analyses, where the outcomes between RCTs and non-RCTs were differentially analyzed. Moreover, the robustness of these findings was confirmed through leave-one-out sensitivity analyses (see Table 5). This approach involved systematically removing individual data points from each study and rerunning the analysis to evaluate the consistency and stability of the results. By iteratively testing the impact of each data point on the overall outcome, leave-one-out sensitivity analysis provided valuable insights into the reliability of the statistical conclusions. Specifically, it allowed us to determine whether the findings were dependent on specific data points or if they held true across the entire dataset. The consistent patterns observed across multiple iterations of the analysis therefore indicated the robustness of the results. Furthermore, when it comes to the threshold to detect passive motion, no significant effect of taping (-0.02) was observed as compared to no comparator, but a significant effect was observed as compared to placebo comparator (-1.35). During the within-group analysis, a *medium* effect (-0.36) improvement was observed in the threshold to perceive passive motion. The change in the threshold to detect passive motion is a crucial measure in assessing proprioception because the test evaluates the ability to perceive passive motion, incorporating passive proprioceptive signals which may differ from consciously perceived tests of proprioception. This assessment is particularly valuable in cases of ACL-deficient knees [156], or individuals with rotator cuff tears [157], as it can identify subtle proprioceptive alterations commonly observed in such conditions. Moreover, the sensitivity and precision of the threshold to detect passive motion provide insights into prognostic outcomes and guide treatment planning. For instance, individuals with higher threshold values may exhibit greater functional impairment, signaling the need for more targeted interventions to improve proprioception and enhance joint stability. Furthermore, a between-group analysis was conducted to evaluate the influence of taping on active movement extent discrimination apparatus. However, no significant influence of taping was observed. This lack of effect on the ability to actively discriminate movements could perhaps be as a result of the high level of ecological validity demonstrated by the active movement extent discrimination apparatus [158]. The test reportedly assesses proprioception functions in conditions more analogous to natural settings [27]. Likewise, its ability to provide accurate and meaningful metrics, rooted in signal detection theory [159], is important as by analyzing response data amidst uncertainty using receiver operating characteristic analysis, the test offers a robust assessment of proprioception. This could potentially explain why modifications in joint position sense and threshold to detection of passive motion were observed, while none were noted in the active movement discrimination test. However, it's worth considering the impact of the number of studies included in the analysis. For instance, in the meta-analysis comparing joint position sense against no comparator, 48 studies were evaluated. In contrast, for threshold to detection of passive motion, eight studies were assessed, and for active movement discrimination apparatus, only two studies were evaluated. The lack of modification observed in active movement discrimination apparatus could be attributed to its reliability in assessing proprioception. However, it's also plausible that the limited number of studies prevented an effect from being observed, potentially due to a type II error [160].

### Influence of taping on healthy and patient population groups

In line with the existing studies where the use of taping has been emphasized to manage deficit joint proprioception [50, 76–78], significant *medium*-to-*large* effect increments for the outcomes of repositioning accuracy with taping were found for healthy population groups (no comparator: -0.29, placebo comparator: -0.61). The increments in repositioning accuracy were also found in population groups with anterior cruciate ligament rupture (no comparator: -0.66), and in individuals with osteoarthritis (placebo comparator: -2.21). These improvements were however, not confirmed in the within-group analyses where non-significant improvement in repositioning accuracy was evident in healthy population groups (g: -0.55, *p* = 0.10). These findings contrast with existing literature suggesting that augmentation of proprioceptive afferent by taping is more beneficial for individuals with poorer inherent proprioception than individuals with good proprioception [161]. The reason behind being that taping augmented proprioceptive afferent information might overload the “inherently good” proprioceptive pathways in healthy individuals. In contrast, individuals with poorer proprioception (i.e., injuries) might benefit from augmented afferent information [126, 162]. Although this theory is widely supported [61, 126, 163], two reasons might explain this differential result in the meta-analysis. First, there was a large difference in the number of studies in the subgroup analysis that evaluated effects of taping on different population groups. For instance, in the within-group analysis, the influence of taping was evaluated on healthy individuals among 15 studies, whereas in the between group analysis with no taping comparator there were 32 studies that had evaluated the effect of taping on healthy individuals. Moreover, in the within group analysis only four, three, and two studies evaluated taping’s impact on ankle instability, stroke, and anterior cruciate ligament reconstruction, respectively. The difference between the number of studies incorporating healthy and patient population group was also evident in between-group meta-analyses (i.e., in no comparator analyses healthy: 32 studies, functional ankle instability: three, patellofemoral pain syndrome: two). Second, in the analyses of healthy population groups, separate sub-group analyses to evaluate the differential influence on individuals with excellent and poor inherent proprioception were not conducted. This analysis was not performed because only a few of the included studies had reported their data differentially according to the intrinsic proprioceptive capabilities of their sample [61, 126]. Future studies are strongly recommended to classify the proprioceptive level of their population groups, as it will help in understanding the actual influence of taping on proprioceptive accuracy among healthy individuals.

### Influence of elastic and rigid *tapes* on proprioception

Various tapes had been used in the existing literature to influence proprioceptive outcomes in healthy and patient population groups (Table S2). However, seldomly some studies have directly compared the influence of different types of tapes on proprioceptive results [49, 56, 90]. In the meta-analyses different tapes were characterized as elastic or rigid tapes based on the description provided in the studies. All the between-group analysis revealed that both the elastic tape (no comparator: -0.40, placebo comparator: -1.13), and rigid tape (no comparator: -0.37, placebo comparator: -1.67) led to a significant improvement repositioning accuracy. The improvement in repositioning accuracy with elastic tape makes sense because previously published literature has demonstrated that tapes with low elastic modulus can support and stabilize the joints without restricting their range of motion [164–166]. Besides, owing to their better elasticity, tapes such as Kinesio tape have been reported to exert a pulling force on the skin, facilitating mechanoreceptors' stimulation [142]. Similarly, enhanced elasticity in the tape has been shown to provide better comfort as it aligns well with the contour of the body, and this could have led to an enhancement in proprioceptive performance [90]. With regards to the rigid tape, the higher elasticity modulus of such tapes could restrict the range of motion at a joint, thereby immobilizing its activity during the injury phase to facilitate healing [167, 168]. However, it's important to note that in some instances documented in the literature, certain rigid tapes have been reported to lose their elasticity rapidly, leading to inadequate restraint of joint motion [169–171].

The analysis did not report differences in the magnitude of effect between rigid (-0.70) and elastic (-0.63) tape during the within-group analyses as well. However, when evaluating the efficacy of these tapes in detecting passive motion thresholds, larger magnitude of improvements was noted in the threshold perception with the elastic tape (-0.26) as compared to the rigid tape (0.04). This difference in efficacy might stem from the restrictive nature of the rigid tapes, which while limiting ankle motion could affect joint forces higher up the kinetic chain, particularly in the knee joint [172]. Furthermore, subgroup analyses were conducted to evaluate the differential influence of elastic and rigid taping on proprioceptive outcomes in both healthy and injured population groups. Significant enhancement in joint proprioception was observed with both types of tape among in healthy individuals, with similar magnitudes of improvement noted for elastic (-0.30) and rigid tape (-0.29). However, among fatigued healthy individuals, although not statistically significant, there was a "medium" effect size improvement in repositioning accuracy with rigid tape (-0.72), contrasting with a "small" effect size improvement seen with elastic tape (-0.08). This difference in magnitude could be likely attributed to the fact that when a muscle or joint is fatigued, it becomes more susceptible to injury [173], and rigid tape can help to prevent this by limiting the range of motion and providing additional support. Likewise, rigid taping could have also restrained motion at the injured ligamentous tissue to its anatomical limits, and could have attenuated fatigue-induced instability, often associated with deficits in neuromuscular control, by improving the altered flow of afferent input to the central nervous system [38, 55, 174].

### Limitations

Despite the novelty of the present meta-analysis, the study has a few limitations. The principal objective of this study was to elucidate the influence of taping on joint repositioning accuracy, the threshold to detection of passive motive, and active movement extent discrimination accuracy. However, upon further assessment of the studies, it was observed that while some of the included studies had evaluated the direct influence of taping [76, 120, 140, 153], others had assessed the influence of the prolonged application of taping on the outcomes of joint proprioception [75, 78, 87, 103, 119, 134]. As it was not the initial goal to evaluate how prolonged taping could influence joint proprioception, separate subgroup analyses to compare the effect of prolonged taping on proprioception were not conducted. Existing studies have suggested that prolonged taping could have a larger impact on movement kinematics and kinetics than immediately after taping [103, 119, 175]. Therefore, future studies are strongly recommended to evaluate the differential influence of the prolonged application of taping on joint proprioception. Secondly, the majority of the studies included in the analysis did not blind assessors, therapists, and subjects, as determined by the PEDro scale used to assess methodological quality. This lack of blinding could have significantly impacted the results, and even though subgroup analyses were conducted to account for the differences between studies with blinding and randomization versus those without, readers are urged to interpret the findings with caution. Thirdly, substantial heterogeneity was also prominent regarding the different taping application methods. For instance, some studies included in the review adhered to a specific taping technique, such as Kenzo Kase’s technique [50, 78, 85], and basket-weave technique [36, 134, 148], whereas the majority had applied taping without following any standardized approach [39, 59, 61, 76, 77, 110]. This heterogeneous approach to using tape complicates understanding of taping’s influence on joint proprioception. Future studies are recommended to adhere to standardized taping applications as they can help develop practical, evidence-based guidelines. Another major limitation of the study was that fewer studies were included in certain meta-analyses, such as between-group analyses of stroke population, individuals with ankle sprain (i.e., two studies), active movement extent discrimination apparatus (two studies), and within-group analysis of threshold to detect passive motion (three studies for overall analysis). The fewer studies could increase the chances of a type II error [176]. Lastly, as the present review mainly incorporated studies that evaluated the influence of taping on joint repositioning accuracy tests, it is important to understand the inherent constraints associated with joint position tests to grasp the overall impact of taping on proprioception [27, 177]. The literature suggests that joint re-positioning tests lack ecological validity because the testing conditions are significantly different from normal daily activities [27, 178]. For instance, conditions such as slow angular velocities, non/partial-weight bearing conditions, absence of auditory and visual feedback, and isolation of the joint under investigation mean that these tasks do not accurately reflect the normal performance of the proprioceptive system in real-world scenarios [27]. Additionally, since joint position sense tests heavily rely on memory and attention, the outcomes may not solely reflect an individual's proprioceptive ability [27, 177]. For example, in cases where an individual has good proprioception but suffers from memory deficits or attention issues, their performance on joint position sense tests may be adversely affected. This suggests that the results of joint position sense tests may not accurately isolate and evaluate proprioceptive function when other cognitive factors come into play. The reader is recommended to infer the results of this review in light of the aforementioned limitations.

### Future directions

Although the number of studies incorporating taping for improving proprioception in healthy and patient population groups has increased in the past decade, a few aspects still warrant exploration. For instance, limited research has evaluated the long-term retention of proprioceptive accuracy after the application of taping [78, 103]. Conventionally, taping has been identified as a transient approach that facilitates performance transiently by guiding the movement when it is being worn. However, once it’s removed, the lack of guidance (see guidance hypothesis [179]) by taping forces improved accuracy back to initial levels [171]. An effective means by which this feedback dependency of taping could be countered by tapering the extent of tactile feedback provided over time. Here, perhaps reducing the length of taping applied [51], or even the tension with which taping is used could reduce the extent of feedback being provided to the performer and allow them to form robust internal feedback/feed-forward models concerning the task at hand. Future studies should try to evaluate these outcomes to ascertain if tactile stimulation via taping can also enhance learning as compared to performance.

## Conclusion

The meta-analysis suggests a positive influence of taping on proprioceptive accuracy outcomes in healthy population groups. The increments for repositioning accuracy were confirmed to be higher in the between group analysis against both placebo and no taping comparators. Besides, subgroup analyses revealed that both elastic taping and rigid taping had similar efficacy in improving repositioning accuracy. Despite the sensitivity analyses confirming the robustness of the findings, readers are recommended to interpret these results cautiously as the studies included in the review were of "fair" methodological quality, and high levels of heterogeneity were observed in the meta-analyses. Nonetheless, the study provides evidence for incorporating taping to promote joint repositioning accuracy.

### Supplementary Information

Supplementary Material 1.

## Data Availability

The datasets used and/or analyzed during the current study are available from the corresponding author on reasonable request.
