# Supplementary material for: Influence of taping on joint proprioception: a systematic review with between and within group meta-analysis
Source: BMC Musculoskelet Disord. 2024 Jun 18;25:480. doi: 10.1186/s12891-024-07571-2 (PMC11186105; doi:10.1186/s12891-024-07571-2)
Supplement: Supplementary file 1 — Supplementary Material 1. [file 12891_2024_7571_MOESM1_ESM.docx]

**Influence of taping on joint proprioception: A systematic review with between and within group meta-analysis**

**Supplementary file**

**Table S1.** PRISMA checklist

| **Section and Topic** | **Item #** | **Checklist item** | **Location where item is reported** |
| --- | --- | --- | --- |
| **TITLE** | | |  |
| Title | 1 | Identify the report as a systematic review. | 1 |
| **ABSTRACT** | | |  |
| Abstract | 2 | See the PRISMA 2020 for Abstracts checklist. | 1 |
| **INTRODUCTION** | | |  |
| Rationale | 3 | Describe the rationale for the review in the context of existing knowledge. | 2-4 |
| Objectives | 4 | Provide an explicit statement of the objective(s) or question(s) the review addresses. | 4 |
| **METHODS** | | |  |
| Eligibility criteria | 5 | Specify the inclusion and exclusion criteria for the review and how studies were grouped for the syntheses. | 5 |
| Information sources | 6 | Specify all databases, registers, websites, organisations, reference lists and other sources searched or consulted to identify studies. Specify the date when each source was last searched or consulted. | 4-5 |
| Search strategy | 7 | Present the full search strategies for all databases, registers and websites, including any filters and limits used. | 5 |
| Selection process | 8 | Specify the methods used to decide whether a study met the inclusion criteria of the review, including how many reviewers screened each record and each report retrieved, whether they worked independently, and if applicable, details of automation tools used in the process. | 5 |
| Data collection process | 9 | Specify the methods used to collect data from reports, including how many reviewers collected data from each report, whether they worked independently, any processes for obtaining or confirming data from study investigators, and if applicable, details of automation tools used in the process. | 5 |
| Data items | 10a | List and define all outcomes for which data were sought. Specify whether all results that were compatible with each outcome domain in each study were sought (e.g. for all measures, time points, analyses), and if not, the methods used to decide which results to collect. | 5 |
|  | 10b | List and define all other variables for which data were sought (e.g. participant and intervention characteristics, funding sources). Describe any assumptions made about any missing or unclear information. | 5 |
| Study risk of bias assessment | 11 | Specify the methods used to assess risk of bias in the included studies, including details of the tool(s) used, how many reviewers assessed each study and whether they worked independently, and if applicable, details of automation tools used in the process. | 5 |
| Effect measures | 12 | Specify for each outcome the effect measure(s) (e.g. risk ratio, mean difference) used in the synthesis or presentation of results. | 5-6 |
| Synthesis methods | 13a | Describe the processes used to decide which studies were eligible for each synthesis (e.g. tabulating the study intervention characteristics and comparing against the planned groups for each synthesis (item #5)). | 6 |
|  | 13b | Describe any methods required to prepare the data for presentation or synthesis, such as handling of missing summary statistics, or data conversions. | 6 |
|  | 13c | Describe any methods used to tabulate or visually display results of individual studies and syntheses. | 6 |
|  | 13d | Describe any methods used to synthesize results and provide a rationale for the choice(s). If meta-analysis was performed, describe the model(s), method(s) to identify the presence and extent of statistical heterogeneity, and software package(s) used. | 6 |
|  | 13e | Describe any methods used to explore possible causes of heterogeneity among study results (e.g. subgroup analysis, meta-regression). | 6 |
|  | 13f | Describe any sensitivity analyses conducted to assess robustness of the synthesized results. | 6 |
| Reporting bias assessment | 14 | Describe any methods used to assess risk of bias due to missing results in a synthesis (arising from reporting biases). | 6 |
| Certainty assessment | 15 | Describe any methods used to assess certainty (or confidence) in the body of evidence for an outcome. | - |
| **RESULTS** | | |  |
| Study selection | 16a | Describe the results of the search and selection process, from the number of records identified in the search to the number of studies included in the review, ideally using a flow diagram. | 7 |
|  | 16b | Cite studies that might appear to meet the inclusion criteria, but which were excluded, and explain why they were excluded. | - |
| Study characteristics | 17 | Cite each included study and present its characteristics. | 8-51, 59-63, Table 1 |
| Risk of bias in studies | 18 | Present assessments of risk of bias for each included study. | 52-58, Table 2 |
| Results of individual studies | 19 | For all outcomes, present, for each study: (a) summary statistics for each group (where appropriate) and (b) an effect estimate and its precision (e.g. confidence/credible interval), ideally using structured tables or plots. | 8-51, 59-63, Table 1 |
| Results of syntheses | 20a | For each synthesis, briefly summarise the characteristics and risk of bias among contributing studies. | 52-58, Table 2 |
|  | 20b | Present results of all statistical syntheses conducted. If meta-analysis was done, present for each the summary estimate and its precision (e.g. confidence/credible interval) and measures of statistical heterogeneity. If comparing groups, describe the direction of the effect. | 63-70, Table 4, 5 |
|  | 20c | Present results of all investigations of possible causes of heterogeneity among study results. | 63-70, Table 4, 5 |
|  | 20d | Present results of all sensitivity analyses conducted to assess the robustness of the synthesized results. | 71-74, Table 6 |
| Reporting biases | 21 | Present assessments of risk of bias due to missing results (arising from reporting biases) for each synthesis assessed. | 11 |
| Certainty of evidence | 22 | Present assessments of certainty (or confidence) in the body of evidence for each outcome assessed. | - |
| **DISCUSSION** | | |  |
| Discussion | 23a | Provide a general interpretation of the results in the context of other evidence. | 75-79 |
|  | 23b | Discuss any limitations of the evidence included in the review. | 75-77 |
|  | 23c | Discuss any limitations of the review processes used. | 78 |
|  | 23d | Discuss implications of the results for practice, policy, and future research. | 78-79 |
| **OTHER INFORMATION** | | |  |
| Registration and protocol | 24a | Provide registration information for the review, including register name and registration number, or state that the review was not registered. | 4 |
|  | 24b | Indicate where the review protocol can be accessed, or state that a protocol was not prepared. | 4 |
|  | 24c | Describe and explain any amendments to information provided at registration or in the protocol. | - |
| Support | 25 | Describe sources of financial or non-financial support for the review, and the role of the funders or sponsors in the review. | - |
| Competing interests | 26 | Declare any competing interests of review authors. | - |
| Availability of data, code and other materials | 27 | Report which of the following are publicly available and where they can be found: template data collection forms; data extracted from included studies; data used for all analyses; analytic code; any other materials used in the review. | - |

*From:*  Page MJ, McKenzie JE, Bossuyt PM, Boutron I, Hoffmann TC, Mulrow CD, et al. The PRISMA 2020 statement: an updated guideline for reporting systematic reviews. BMJ 2021;372:n71. doi: 10.1136/bmj.n71

Table S2 Description of included studies

| Study  Design | Health status/ medical condition  *Joint assessed* | Sample size (N)  Gender distribution (F, M)  (Age in years as Mean ± SD/ range) | Taping methods | Proprioception assessment | Taping technique | Taping applicant  *Taping frequency* | Timing of post-test | Results |
| --- | --- | --- | --- | --- | --- | --- | --- | --- |
| F Fazli, A Farsi, IE Takamjani, S Mansour, N Yousefi and F Azadinia [95]  Randomized controlled design | Osteoarthritis  *Knee* | KT: N= 28  17F, 11M  (54.4 ± 3.2)  *KO: N= 28  11F, 17M  (55.7 ± 5.3) | KT: Kinesio tape  *KO: Knee orthosis | Absolute active RE during flexion for a target angle of 45º  Absolute active RE during flexion for a target angle of 70º  TTDPM at 0.25º/sec with 45º flexion starting position  WOMAC-OA index (stiffness, pain, physical function) | KT: Applied according to Kase’s technique [24]  I-strip applied on quadriceps femoris along rectus femoris, vastus lateralis, vastus medialis  Rectus femoris: applied 10 cm below ASIS to superior border of patella (50% to 70% stretch), knee flexed at 45º and rest of strip applied towards tibial tuberosity (0% stretch)  Vastus medialis: applied from middle 1/3^rd^ medial side of thigh to medial border of patella (50% to 75% stretch), knee flexed at 45º and rest of strip applied towards tibial tuberosity (0% stretch)  Vastus lateralis: applied from under the greater trochanter to lateral border of patella (50% to 75% stretch), knee flexed at 45º and rest of strip applied towards tibial tuberosity (0% stretch) | -  Once a week for four weeks (KT stayed for 5 consecutive days) | Pre-KT  Post-KT: after 4 weeks | Significant ↓ in absolute active RE with KT during flexion for a target angle of 45º  No difference in absolute active RE with KT during flexion for a target angle of 70º  No difference in TTDPM with KT  Significant ↓ in subjective WOMAC-OA scores with KT |
| EP Kisa and BK Kaya [96]  Cross sectional design | Healthy (sports personnel)  *Shoulder* | N= 27  ?F, ?M  (11 to 14 years) | KT: Kinesio tape | Absolute active displacement test (cm) for RE during glenohumeral forward flexion at 90 º forward flexion | KT: Applied from origin to insertion with 50% tension according to L Pyšný, J Pyšná and D Petrů [97]  Procedure: I-strip applied to provide activation of only lower trapezius and continued form the lower trapezius to upper trapezius along medial border of scapula on the dominant side | Clinician  - | Pre-KT  Post-KT: immediately after | Significant ↓ in absolute active displacement RE with KT |
|  | Scapular asymmetry (sports personnel)  *Shoulder* | N= 16  ?F, ?M  (11 to 14 years) |  |  |  |  |  | Significant ↓ in absolute active displacement RE with KT |
| İ Poyraz and Ö Vergili [98]  Pretest-posttest quasi-experimental design | Healthy (non-professional sports personnel)  *Knee* | N= 13  2F, 11M  (12.4) | KT: Kinesio tape | Absolute active RE during extension for a target angle of 45º | KT: Applied on quadriceps femoris muscle with facilitation technique from origin to insertion  Step 1: Anchor applied at the origin of muscle to quadriceps muscle in the tense position with knee in maximum flexion  Step 2: I-strip applied on the patellar tendon with applying tension from anterior superior iliac spine | Not reported  - | Pre-KT  Post-KT: after 1-week, after 2-week | No difference in absolute active RE with KT during extension for a target angle of 45º after 1-week and after 2-week |
|  | Healthy fatigue (non-professional sports personnel)  *Knee* |  |  |  |  |  | Post-KT fatigue:  after 1-week, after 2-week | No difference in absolute active RE with KT during extension for a target angle of 45º after 1-week and after 2-week of fatigue protocol |
| M Maqsood and M Váczi [99]  Randomized crossover design | Healthy  *Knee* | N= 12  5F, 7M  (20.7 ± 2.1) | KT: Kinesio tape  NT: No tape | Absolute active RE during plantarflexion for a target angle randomly selected between 15º, 20º, 25º, 75º, 80º and 85º | KT: Applied according to [24] (35 to 40% stretch longitudinally and diagonally) with knee flexed at 90º in origin to insertion direction for rectus femoris, vastus lateralis, and medialis | Not reported  - | Pre-KT  Post-KT: Immediately after | No difference in absolute active RE with KT as compared to NT |
| F Saki, A Shayesteh, F Ramezani and S Shahheidari [100]  Randomized controlled design | Medial tibial stress syndrome  *Ankle* | KT: N= 16  16M  (26.2 ± 3.9)  PT: N= 16  16M  (24.6 ± 2.7) | KT: Kinesio tape  PT: Placebo tape | Absolute active RE during plantarflexion for a target angle of 20º  Absolute active RE during dorsiflexion for a target angle of 10º | KT: Kinesio tape applied with 75% stretch  Step 1: Y-strip applied on proximal 1/3^rd^ of medical part of tibia  Step 2: One half of the Y-strip passed over anterior part of tibia and medial malleolus  Step 3: Other half of the tape passed over posterior part of the leg and inner ankle and ended under the medial longitudinal arch of the foot  PT: Placebo KT applied (0% stretch) | Not reported  - | Pre-KT  Post-KT: Immediately after | Significant ↓ in absolute active RE with KT as compared to PT during plantarflexion for a target angle of 20º  Significant ↓ in absolute active RE with KT as compared to PT during dorsiflexion for a target angle of 10º |
| F Shams, M Hadadnezhad, A Letafatkar and J Hogg [101]  Randomized controlled design | Dynamic knee valgus  *Knee* | MT Py: N= 16  16F  (24.9 ± 4.5)  NT Py: N= 16  16F  (24 ± 3.9)  NT: N= 16  16F  (24.4 ± 4) | MT Py: Mulligan tape with plyometrics  NT Py: No tape with plyometrics  NT: No tape | Absolute active RE during flexion for a target angle of 45º in closed kinetic chain | MT: Applied according to Mulligan technique with knee flexed at 25º and hip internally rotated, MT applied in spiral manner to the fibular neck across the front of the tibia while applying internal tibial torsion. Then tape was passed posteriorly and inferiorly to medial knee joint line and centrally over posterior aspect of knee joint. Tape continued in this direction and finished at the lateral lower 1/3^rd^ of thigh | Not reported  - | Pre-MT  Post MT: after six weeks | Significant ↓ in absolute active RE with MT Py as compared to NT Py and NT during flexion for a target angle of 45º |
| P Mehta, AJ Prabhakar and C Eapen [70]  Randomized controlled design | Healthy  *Shoulder* | KT: N= 20  ?F, ?M  (26.1 ± 7.6)  PT: N= 20  ?F, ?M  (24.5 ± 5.5) | KT: Kinesio tape  PT: Placebo tape | Absolute active RE during flexion for a target angle of 60º  Absolute active RE during flexion for a target angle of 90º  Absolute active RE during flexion for a target angle of 120º  Absolute active RE during abduction for a target angle of 60º  Absolute active RE during abduction for a target angle of 90º  Absolute active RE during abduction for a target angle of 120º  Absolute active RE during external rotation for a target angle of 30º  Absolute active RE during external rotation for a target angle of 45º  Absolute active RE during external rotation for a target angle of 90º | KT: Y-strip and I applied from origin to insertion with muscle (supraspinatus, deltoid, infraspinatus, pectoralis major) placed in a stretched position, applied in two steps  Step 1: Base of KT applied at the origin with no tension (0% stretch). Once the base was fixed, stress spread (50-55% stretch) evenly along the length of the strip  Step 2: Once the strip reached one to two inches near insertion, the tape was applied to the insertion with no tension (0% stretch)  KT rubbed along the length to ensure proper fixation  PT: KT applied on the superior and lateral aspects of the shoulder | Not reported  *Once* | Pre-KT  Post-KT: immediately after KT | Significant ↓ in absolute active RE with KT as compared to PT during flexion for a target angle of 60º  Significant ↓ in absolute active RE with KT as compared to PT during flexion for a target angle of 90º  No difference in absolute active RE during flexion for a target angle of 120º  No difference in absolute active RE during abduction for target angles 60º, 90º, 120º  No difference in absolute active RE during flexion for a target angle of 120º  No difference in absolute active RE during external rotation for target angles 30º, 45º  Significant ↓ in absolute active RE with KT as compared to PT during external rotation for a target angle of 90º |
| D Kielė and R Solianik [72]  Randomized controlled design | Anterior cruciate ligament rupture  *Knee* | KT: N= 16  16M  (24.7 ± 3.7)  NT: N= 16  16M  (26.2 ± 8.7) | KT: Kinesio tape with physiotherapy  NT: No tape with physiotherapy | Absolute active RE during flexion for target angle 40º  Absolute active RE during flexion for target angle 80º  Tegner activity scale | KT: K-active Kinesio tape was applied to the injured leg using muscle facilitation and functionally corrective technique according to Kase’s procedure [102]; KT applied in two steps  Step 1: Y-strip applied in origin to insertion direction to facilitate muscle with the subject lying in the lateral position, Y-strip applied on quadriceps femoris with knee 90º flexed (25-30% stretch), then KT applied on biceps femoris (30% stretch) by bringing hip to flexion and knee to extension  Step 2: I-strip applied with the knee in 20 to 30º flexion on tibial tuberosity to the medial and lateral condyle of the femur (75-100% stretch) to limit anterior tibial translation | Physical therapist  *KT changed 3 to 5 days for four weeks* | Pre-KT  Post-KT: after 4 weeks | Significant ↓ in absolute active RE with KT as compared to NT during knee flexion for a target angle of 40º  Significant ↓ in absolute active RE with KT as compared to NT during knee flexion for a target angle of 80º  No difference in Tegner activity scores for physical activity with KT as compared to NT |
| JH Kim, KH Kim and DH Kim [71]  Randomized controlled design | Stroke  *Knee* | KT: N=20  9F, 11M  (53.7 ± 9.6)  PT: N=20  7F, 13M  (54.4 ± 7.2) | KT: Kinesio tape with proprioceptive training  PT: Placebo tape with proprioceptive training | Absolute active RE during extension for target angle (randomly one angle chosen) 30°, 60°, 90°, 120°, 150° | KT: Applied with Y-strip and I-strip  Strip 1: I-strip applied from knee ligament below knee cap to middle area of rectus femoris (10-15% stretch)  Strip 2: Y-strip unsplit end applied at knee cap below the knee ligament (30% stretch), then one split end attached to the middle of vastus lateralis, the other split end applied to the middle area of vastus medialis  PT: KT applied horizontally to knee cap and middle area of quadriceps femoris muscle | Physical therapist  *Once* | Pre-KT  Post-KT: after four weeks | Significant ↓ in absolute active RE with KT as compared to PT during extension |
| P Li, Z Wei, Z Zeng and L Wang [103]  Randomized cross over design | Functional ankle instability  *Ankle* | N= 28  13F, 15M  (21.2 ± 2) | FKT: Facilitatory Kinesio tape  ABT: Ankle balancing tape  PT: Placebo tape  NT: No tape | Absolute RE during dorsiflexion for target angle 5º  Absolute RE during plantarflexion for target angle 5º  Variable RE during plantarflexion for target angle 5º  Variable RE during dorsiflexion for target angle 5º | FKT: Applied according to T Halseth, WM John and D Mark [104] while maintaining 50% stretch  ABT: Applied according to BG Lee and JH Lee [105] while maintaining 50% stretch  PT: Applied with 0% stretch | Physical therapist  Once | Pre-KT  Post-KT: immediately after | No difference in absolute RE during dorsiflexion and plantarflexion between FKT, ABT, PT and NT for target angle 5º  No difference in variable RE during dorsiflexion and plantarflexion between FKT, ABT, PT and NT for target angle 5º |
| H-S Chen, Y-Z Chang, C-M Fang, C-Y Lin and W-C Yang [44]  Cross over design | Healthy (sports personnel)  *Hip, knee* | N= 13  ?F, ?M  (?) | KT: Kinesio tape  NT: No tape | Absolute RE during dominant hip flexion for target angle 60º  Absolute RE during dominant knee flexion for target angle 60º  Absolute RE during non-dominant knee flexion for target angle 60º | - | Not reported  - | Pre-KT  Post-KT: immediately after | Significant ↓ in absolute active RE with KT as compared to NT during dominant hip flexion for a target angle of 60º  Significant ↓ in absolute active RE with KT as compared to NT during dominant knee flexion for a target angle of 60º  Significant ↓ in absolute active RE with KT as compared to NT during non-dominant knee flexion for a target angle of 60º |
| M Saran, S Pawaria and S Kalra [68]  Randomized controlled design | Healthy (sports personnel)  *Shoulder* | KT: N= 15  15M  (25.6 ± 2.1)  NT: N= 15  15M  (24.8 ± 2.7) | KT: Kinesio tape with ballistic plyometric training  NT: No tape with ballistic plyometric training | Absolute active RE during internal rotation for 90% of internal rotation maximum range  Absolute active RE during external rotation for 90% of external rotation maximum range | KT: Applied with a “Y” pattern with subjects’ arm lying on arm rest and elbow at 90º | Not reported  *Once* | Pre-KT  Post-KT: after 8 weeks | Significant ↑ in RE with KT as compared to NT during external, internal rotation for 90% of internal rotation maximum range after eight weeks |
| F Saki, H Romiani, M Ziya and N Gheidi [69]  Randomized controlled design | Dynamic knee valgus (sports personnel)  *Knee* | KT: N= 12  12F  (18.9 ± 2.5)  PT: N= 12  12F  (19.2 ± 1.8) | KT: Kinesio tape  PT: Placebo tape | Absolute active RE during flexion for target angle 30º | KT: Applied for gluteus medius, tibialis anterior, applied in four steps  Step 1: Y-strip applied from iliac crest to the greater trochanter in side position with hip flexed, adducted and internally rotated in 90º, unsplit end of Y-strip applied on the lateral surface of the greater trochanter (0% stretch), anterior split end used towards the anterior superior iliac spine (15-25% stretch) and posterior split end towards the posterior superior iliac spine (15-25% stretch) or paper-off tension, last 1-2 inches applied with no tension  Step 2: Applied with an I-strip on the tibialis anterior, ankle position in plantarflexion and inversion, KT applied from the proximal lateral portion of the tibia to the first metatarsal and medial cuneiform, then the base of KT applied on the proximal lateral part of the tibia with no tension, then spread with stress (15-25% stretch) or paper-off tension, last 1-2 inches applied with no pressure, glue activated at the end  PT: Applied similarly to KT but without hip flexion, adduction, and internal rotation for gluteus medius KT and ankle plantarflexion, eversion foot for tibialis anterior KT | Not reported  *Once* | Pre-KT  Post-KT: 3 days after KT | Significant ↓ in RE with KT as compared to PT during flexion for target angle 30º |
| CG Bayu, M Andriana and A Pawana [58]  Pretest-posttest quasi-experimental design | Stroke  *Knee* | N= 8  4F, 4M  (46.3 ± 11.0) | MPT: McConnell patellar taping, rigid tape | Absolute active RE during flexion for target angle 15º  Absolute active RE during flexion for target angle 30º  Absolute active RE during flexion for target angle 60º  TTDPM at 1º/sec | MPT: Applied by affixing the knee joint positioned and pulled medially, i.e., from the lateral kneecap covering the medial end of the semitendinosus muscle | Not reported  *Once* | Pre-MPT  Post-MPT: 30 minutes after MPT | Significant ↓ in TTDPM with MPT, at 30-min post MPT as compared to pre-MPT  Significant ↑ in RE with MPT during flexion for a target angle of 15º, at 30-min post MPT as compared to pre-MPT  Significant ↑ in RE with MPT during flexion for a target angle of 30º, at 30-min post MPT as compared to pre-MPT  No difference in RE with MPT during flexion for a target angle of 60º, at 30-min post MPT as compared to pre-MPT |
| HE Göktaş, S Çitaker and ED Yurtsever [51]  Randomized controlled design | Sub acromial impingement syndrome  *Shoulder* | DT: N=16  11F, 5M  (51.1 ± 11.6)  PT: N=15  10F, 5M  (50.6 ± 8.0) | DT: Dynamic tape  PT: Placebo tape | Absolute RE during internal rotation for target angle 30º  Absolute RE during external rotation for target angle 30º | DT (Indirect method of upper extremity load bearing): Applied in four steps according to [85]  Step 1: 45º to 70º abduction, 20º horizontal extension, DT applied 5cm above olecranon process    Step 2: 30º to 45º horizontal flexion, DT stretched to create a force vector in the superior direction  Step 3: Shoulder flexed, and DT stretched to maintain upward rotation of the scapula and applied anteriorly to prevent anterior translation of the humeral head  Step 4: Tensionless DT 5cm applied obliquely down the scapula | Physical Therapist  *Once* | Pre-DT  Post-DT: after one week | No difference in RE between DT and PT during internal rotation  No difference in RE between DT and PT during internal rotation |
| K Grütters, S Narciss, SM Beaudette and L Oppici [106]  Randomized controlled design | Healthy  *Lumbar* | LT: N=6  ?F, ?M  (23 ± 3)  KT: N=5  ?F, ?M  (23 ± 3) | LT: Leuko tape  KT: Kinesio tape | Absolute active RE during lumbar flexion angle between T12 and S1 vertebrae for target angle: 30º  Absolute active RE during lumbar flexion angle for target angle: 60º  Pre/post lifting task | Initial position: standing with a neutral spine  LT: applied on the left and right extensor muscles from the T12 to S1 vertebras  KT: KT applied with tension (10-15% baseline stretch) from T12 to S1 vertebras | Researcher  *LT: six times (re-applied after each lifting task as the tape peeled off)*  *KT: Once* | Pre-LT/KT  Post-LT/KT: 60 minutes after LT/KT | Significant ↓ in absolute active RE in KT as compared to LT for both target angles (i.e., 30º, 60º) and before/after lifting task  Small-to-moderate ↓ effect size reduction in RE after lifting with KT for a 30º target angle  No difference in RE after lifting with LT for a 30º target angle  No difference in RE after lifting with KT for a 60º target angle  Small effect size ↓ in RE after lifting with LT for 60º target angle |
| C Boonkerd, K Thinchuangchan, N Chalarak, S Thonpakorb, R Wanasoonthontham, T Kitsuksan and T Laddawong [107]  Randomized crossover design | Chronic ankle instability  *Ankle* | N= 28  28M  (22.5 ± 3.8) | KT: Kinesio tape  PT: Placebo tape  NT: No tape | Absolute active RE during inversion for target angle 15º  Absolute active RE during inversion for maximum inversion range  Absolute passive RE during inversion for target angle 15º  Absolute passive RE during inversion for maximum inversion range  TTDPM for inversion, eversion with angular velocity 5º/sec | KT: Three strips used on the ankle according to Kase’s technique [24]  Strip 1: applied on the tibialis anterior, from origin (0% tension), ankle plantar flexed and inverted, KT attached (on to lateral malleolus 15-35% stretch)  Strip 2: Applied to activate the peroneal muscle, feet placed in plantarflexion  with inversion and KT fixed on the origin of forces (0% stretch), then KT applied on medial malleolus (15-35% stretch), end of KT pushed across arch (0% stretch) to insertion  Strip 3: Figure-of-eight application to facilitate talofibular ligament function, KT applied along anterior talofibular (15-35% stretch) ligament from inner portion of the tarsal navicular joint to outer ankle, KT applied above the ankle (75-100% stretch) and the inner side of the ankle (15-25% stretch), KT pulled across foot (15-25% stretch) and angled down towards heel over the anterior talofibular ligament  KT rubbed after application to activate adhesive  PT: 2 KT strips without stretch  Strip 1: wrapped outer and inner ankles  Strip 2: tied to the front and back of the leg | Physical Therapist  *Once* | Pre-KT/PT  Post-KT: immediately after KT/PT  The 3-days gap between each condition (KT/PT) | Significant ↓ in passive RE during maximum inversion in KT as compared to PT and NT  No difference in TTDPM for inversion, eversion between KT, PT, NT  No difference in active RE for inversion for both target angles between KT, PT, NT  No difference in passive RE during inversion for target angle 15º in KT as compared to PT and NT |
| Y-S Chen, W-C Tseng, C-H Chen and Y-X Lu [75]  Randomized cross over design | Healthy  *Ankle* | N= 10  10M  (22.3 ± 1.7) | KT 0%: Kinesio tape with 0% stretch  KT 50%: Kinesio tape with 50% stretch  KT 100%: Kinesio tape with 100% stretch  NT: No tape | Absolute active RE during dorsiflexion for target angle 5º  Absolute active RE during dorsiflexion for target angle 10º  Absolute active RE during dorsiflexion for target angle 15º  Absolute passive RE during dorsiflexion for target angle 5º  Absolute passive RE during dorsiflexion for target angle 10º  Absolute passive RE during dorsiflexion for target angle 15º  Constant active RE during dorsiflexion for target angle 5º  Constant active RE during dorsiflexion for target angle 10º  Constant active RE during dorsiflexion for target angle 15º  Constant passive RE during dorsiflexion for target angle 5º  Constant passive RE during dorsiflexion for target angle 10º  Constant passive RE during dorsiflexion for target angle 15º | KT 0%: Applied on tibialis anterior with I-strip technique (0% stretch) according to Kenzo Kase’s method [24]  KT 50%: Similar to KT 0% but applied with 50% stretch  KT 100%: Similar to KT 0% but applied with 100% stretch | Physical Therapist  *Once* | Pre-KT 0%/ KT 50%/ KT 100%  Post-KT 0%/ KT 50%/ KT 100%: 1 day after with KT 0%/ KT 50%/ KT 100%: | No difference in absolute active RE with KT 0%, KT 50%, and KT 100% as compared to NT during dorsiflexion for target angles 5º, 10º, 15º  No difference in absolute passive RE with KT 0%, KT 50%, and KT 100% as compared to NT during dorsiflexion for target angles 5º, 10º, 15º  No difference in constant active RE with KT 0%, KT 50%, and KT 100% as compared to NT during dorsiflexion for target angles 5º, 10º, 15º  No difference in constant passive RE with KT 0%, KT 50%, and KT 100% as compared to NT during dorsiflexion for target angles 5º, 10º, 15º |
| R Adams, C Ganderton, J Han, G Waddington, J Witchalls and Z Yang [46]  Randomized cross over design | Chronic ankle instability  *Ankle* | N= 17  ?F, ?M  (?) | KT: Kinesio tape  KT shoe: Kinesio tape with shoes  NT: No tape | Area under the curve for ankle inversion discrimination apparatus for landing | - | Not reported  *Once* | Pre-KT/KT shoe  Post-KT/KT shoe: immediately after | Significant ↑ in the area under the curve for ankle inversion discrimination apparatus with KT as compared to NT  Significant ↑ in the area under the curve for ankle inversion discrimination apparatus with KT shoe as compared to NT |
|  | Healthy  *Ankle* | N= 17  ?F, ?M  (?) |  |  |  |  |  | Significant ↑ in the area under the curve for ankle inversion discrimination apparatus with KT as compared to NT  No difference in the area under the curve for ankle inversion discrimination apparatus with KT shoe as compared to NT |
| R Yu, Z Yang, J Witchalls, R Adams, G Waddington and J Han [45]  Randomized cross over design | Chronic ankle instability  *Ankle* | N= 15  8F, 7M  (23.9 ± 2.6) | KT short: Kinesio tape short taping  KT mid: Kinesio tape mid taping  KT long: Kinesio tape long taping  NT: no tape | Area under the curve for ankle inversion discrimination apparatus for landing at target angles 10º, 12º, 14º, 16º | KT short: Applied in four strips with moderate tension (25-35% stretch) of different colors only on the foot and ankle complex  Strip 1: Yellow strip started from the dorsum of the foot around the first cuneiform and first metatarsal bones extending upwards along the anterior tibialis muscle  Strip 2: Purple strip started from the first metatarsal and passed around the lateral malleolus, and extended proximally to just distal to the proximal head of the fibula  Strip 3: Red strip applied at the medial malleolus and extended posteriorly along the posterior-medial margin of the tibia and fibula  Strip 4: Green strip applied just anterior to lateral malleolus extended under the plantar aspect of the foot and was pulled up the transverse arch of the foot  KT mid: similar to KT short but applied below the knee  KT long: similar to KT short but applied above the knee | Physical Therapist  *Once* | Pre-KT short/KT mid/KT long  Post- KT short/KT mid/KT long: 24 hours after KT | Significant ↑ in the area under the curve for ankle inversion discrimination apparatus with KT as compared to NT  No difference in the area under the curve for ankle inversion discrimination apparatus with KT short as compared to NT  Significant ↑ in the area under the curve for ankle inversion discrimination apparatus with KT mid as compared to NT  Significant ↑ in the area under the curve for ankle inversion discrimination apparatus with KT long as compared to NT |
|  | Healthy  *Ankle* | N= 15  7F, 8M  (23.1 ± 2) |  |  |  |  |  | No difference in the area under the curve for ankle inversion discrimination apparatus with KT short as compared to NT  No difference in the area under the curve for ankle inversion discrimination apparatus with KT mid as compared to NT  Significant ↑ in the area under the curve for ankle inversion discrimination apparatus with KT long as compared to NT |
| E Smyth, G Waddington, J Witchalls, P Newman, J Weissensteiner, S Hughes, T Niyonsenga and M Drew [74]  Randomized controlled design | Healthy (sports personnel with fatigue)  *Ankle* | N=53  53F  (17.7)  26 and 27 individuals completed both LT SEP and LT self-taping conditions | LT SEP: Leuko tape applied by sports and exercise physiotherapist | AMEDA during inversion for five angles at 1º intervals between 10º to 14º  Pre/post fatigue (netball session) | LT SEP: Skin prep and microfoam applied to anterior and posterior aspects of the ankle, initial position ankle dorsiflexed, LT anchor applied on: distal 1/3^rd^ of shin, two times anchor applied medially to lateral stirrups, two times anchors starting and ending laterally, one half heel lock starting and ending laterally, one half heel lock starting and ending medially, two to three closing anchors ensuring full skin coverage at the bottom 1/3^rd^ of leg | Physical Therapist  *Once* | Pre-LT SEP/LT self  Post-LT SEP/LT self: -, netball training, LT removed | Significant ↑ in AMEDA score with LT SEP pre-fatigue (after the application of LT SEP)  Significant ↑ in AMEDA score with LT SEP post-fatigue (after the netball session)  Significant ↑ in AMEDA score post-fatigue (after the netball session) with LT SEP removed |
|  |  |  | LT self: Elastoplast Leuko tape applied by the athletes on themselves |  | LT self: Similar to LT SEP but tape applied by the athletes on themselves | No  *Once* |  | Significant ↑ in AMEDA score with LT self, pre-fatigue (after the application of LT self)  Significant ↑ in AMEDA score with LT self, post-fatigue (after the netball session)  Significant ↑ in AMEDA score post-fatigue (after the netball session) with LT self, removed |
| M Alawna, B Unver and E Yuksel [77]  Randomized controlled design | Healthy  *Ankle* | *ZnOT: N=37  37F  (23.2 ± 2.8)  Bandaging: N=37  14F, 23M  (22.9 ± 4.1) | *ZnOT: Zinc oxide tape, rigid tape | Absolute active RE during dorsiflexion for target angle 10º  Absolute active RE during neutral position  Absolute active RE during plantarflexion for target angle 10º  Absolute active RE during plantarflexion for target angle 20º | ZnOT: Applied in three steps  Step 1: Anchor tape applied circumferentially above the malleolar level at the lower end of the shank  Step 2: Stirrup was applied as the foot was held in the neutral position, and the tape passed from the medial side of the ankle under the foot over the heel area along the lateral side of the ankle. The same procedure was repeated twice for the second stirrup  Step 3: Two ends of the stirrups were firmly attached to the anchor tape during the first step, and the attachment was reinforced with locking tape, which was applied circumferentially above the malleolar level at the lower end of the shank | Physical Therapist  *Once* | Pre-ZnOT  Post-ZnOT: 20 minutes, 24 hours, 24 hours ZnOT removed | Significant ↓ in RE during dorsiflexion for target angle 10º at assessment periods 20-min post-ZnOT, 24-hour post-ZnOT as compared to pre-ZnOT and 24 hours after without ZnOT  Significant ↓ in RE during neutral position at assessment periods 20-min post-ZnOT, 24-hour post-ZnOT as compared to pre-ZnOT and 24-hour post without ZnOT  Significant ↓ in RE during plantarflexion for target angles 10º, 20º at assessment periods 20-min post-ZnOT, 24-hour post-ZnOT as compared to pre-ZnOT and 24-hour post without ZnOT |
| F Binaei, R Hedayati, M Mirmohammadkhani, C Taghizadeh Delkhoush and R Bagheri [76]  Randomized controlled design | Functional ankle instability  *Ankle* | Weight-bearing exercise with KT: N=19  ?F, ?M  (29.7 ± 8.1)  Weight-bearing exercise NT: N=18  ?F, ?M  (29.7 ± 8.1)  NT: N=19  ?F, ?M  (29.7 ± 8.1) | KT: Kinesio tape  NT: No tape | Absolute active RE during inversion for target angle 5º  Absolute active RE during plantarflexion for target angle 15º  Absolute passive RE during inversion for target angle 5º  Absolute passive RE during plantarflexion for target angle 15º | KT: applied in four steps, with the foot in partial plantarflexion  Strip 1: Applied from anterior mid-foot to area immediately inferior to tibial tuberosity over tibialis anterior (115-120% stretch)  Strip 2: applied to the upper part of the medial malleolus, then spread through the heel (i.e., placed laterally to strip 1), used at the end on a location near the fibular head  Strip 3: Applied on the anterior surface of the ankle to cover medial and lateral malleoli  Strip 4: Applied from mid-portion of longitudinal arc and stretched (6 inches) above medial and lateral malleoli | Not reported  *18 times (3 times per week for six weeks)* | Pre-KT  Post-KT: 6-weeks | Significant ↓ in RE in weight-bearing exercise with KT as compared to NT in active condition for both inversion (5º), plantarflexion (15º)  No difference in RE in weight-bearing exercise with KT as compared to weight-bearing training NT in active condition for both inversion (5º), plantarflexion (15º)  No difference in RE in weight-bearing exercise with KT as compared to weight-bearing NT, NT in passive condition for both inversion (5º), plantarflexion (15º) |
| Z-M Lin, J-F Yang, Y-L Lin, Y-C Cheng, C-T Hung, C-S Chen and L-W Chou [42]  Randomized cross over design | Healthy  *Wrist* | N=24  12F, 12M  (22.9 ± 1.5) | KT 20%: Kinesio tape with 20% additional stretch  PT: Placebo tape  NT: No tape | Absolute active RE during flexion for target angle 15º | KT 20%: Y-shape strip applied on the skin (0% stretch) above finger flexors with an anchor set at the wrist (20% stretch in the middle section), then KT extended to tendon region of finger flexor  PT: Similar to KT but with 0% stretch and no stress on the ends of the tape | Not reported  *Once* | Pre-KT/KT 20%  Post- KT/KT 20%: - | Significant ↓ in RE with KT, KT 20% as compared to NT during flexion |
| ME Ucuzoglu, B Unver, DC Sarac and G Cilga [108]  Randomized controlled design | Healthy  *Wrist* | *RT: N= 34  ?F, ?M  (23.1 ± 1.5)  Bandaging: N= 34  ?F, ?M  (22.5 ± 1) | *RT: Rigid tape (OctaCare) | Absolute active RE during flexion for target angle 30º  Absolute active RE during extension for target angle 30º  Absolute active RE during radial deviation for target angle 10º  Absolute active RE during ulnar deviation for target angle 15º | RT: Applied on the wrist in the neutral position. First, the hypoallergenic tape was applied first with no tension (0% stretch), and then RT applied in X-taping form | Not reported  *Once* | Pre-RT  Post-RT: 20 minutes, 24 hours, 24 hours RT removed | Significant ↓ in RE with RT during flexion for target angle 30º at assessment periods 20-min post-RT, 24-hour post-RT, 24-hour post without RT as compared to pre-RT  Significant ↓ in RE with RT during extension for target angle 30º at assessment periods 20-min post-RT, 24-hour post-RT, 24-hour post without RT as compared to pre-RT  Significant ↓ in RE with RT during radial deviation for target angle 10º at assessment periods 20-min post-RT, 24-hour post-RT, 24-hour post without RT as compared to pre-RT  Significant ↓ in RE with RT during ulnar deviation for target angle 15º at assessment periods 20-min post-RT, 24-hour post-RT, 24-hour post without RT as compared to pre-RT |
| J-T Han [109]  Randomized crossover design | Healthy  *Ankle* | N= 13  5F, 8M  64.4 ± 6.95 | KT: Kinesio tape  PT: placebo tape  NT: No tape | Absolute active RE during plantarflexion for target angle 20º | KT: Applied in an initial position with calf stretched according to J-h Lee and W-g Yoo [110] from the proximal gastrocnemius muscle insertion to calcaneus bone with tension (15-20% stretch), KT applied in four steps  Step 1: KT anchored at heel with ankle joint in the neutral position  Step 2: Calf muscle stretched  Step 3: Y-strip divided the proximal end of the tape and then attached to the medial and lateral end of the gastrocnemius muscle  Step 4: I-strip applied from the posterior surface of the calcaneus to the upper part of the gastrocnemius junction  PT: KT was applied in three strips on the heel and medial, and lateral head of the gastrocnemius muscle | Physical Therapist  *Once* | Pre-KT/PT  Post-KT/PT: immediately after with KT/PT | Significant ↓ in RE with KT as compared to NT during plantarflexion for target angle 20º |
| A Trost [111]  Randomized controlled design | Knee pain  *Knee* | KT: N= 11  ?F, ?M  (68.5 ± 15.6)  PT: N= 11  ?F, ?M  (71.8 ± 10.6) | KT: Kinesio tape  PT: Placebo tape | Absolute active RE during extension for target angle 30º in the open kinetic chain  Absolute active RE during extension for target angle 30º in the closed kinetic chain  Relative active RE during extension for target angle 30º in the open kinetic chain  Relative active RE during extension for target angle 30º in the closed kinetic chain  Knee injury and osteoarthritis outcome score (symptoms, pain, activities of daily living, sports and recreation, and quality of life) | KT: Applied for quadriceps femoris activation in three steps  Step 1: With the leg extended, I-strip was applied 10 cm below the anterior superior iliac spine, then along the course of rectus femoris, KT was applied under tension (50% stretch) until the border of the patella, knee then flexed to 45º and remaining strip used without pressure over the superior border of the patella  Step 2: Base of second I-strip applied to the greater trochanter and tape applied with tension (50% stretch) along the course of vastus lateralis until the lateral border of the patella, knee then flexed to 45º and remaining strip applied without tension over the lateral border of the patella  Step 3: Base of third I-strip applied from middle 1/3^rd^ of medial aspect of the thigh, then tape pulled with tension (50% stretch) along vastus medialis towards the medial border of the patella, knee then flexed to 45º and remaining strip applied without pressure over the medial border of patella ending about tibial tuberosity  PT: KT applied incorrectly from origin to insertion of the rectus femoris (0% stretch), starting with the leg straight and then bending to 45º | Not reported  *Once* | Pre-KT  Post-KT:  Three days, three days KT was removed, and three days more after KT removed | No difference in absolute RE with KT as compared to PT during extension for target angle 30º in the open kinetic chain at 0-day, three days, and after KT removed after three days  Significant ↓ in absolute RE with KT as compared to PT during extension for target angle 30º in the close kinetic chain at 0-day, three days, and after KT removed after three days  No difference in relative RE with KT as compared to PT during extension for target angle 30º in the open kinetic chain at 0-day, three days, and after KT removed after three days  Significant ↓ in relative RE with KT as compared to PT during extension for target angle 30º in the close kinetic chain at 0-day, three days, and after KT removed after three days  Significant ↓ in Knee injury and osteoarthritis outcome score in KT and PT as compared to NT |
|  | Healthy  *Knee* | N= 11  ?F, ?M  (51.5 ± 20.5) |  |  |  |  |  | No difference in absolute RE with KT during extension for target angle 30º in the open kinetic chain at 0-day, three days, and after KT removed after three days  Significant ↓ in absolute RE with KT during extension for target angle 30º in the close kinetic chain at 0-day, three days, and after KT removed after three days  No difference in relative RE with KT during extension for target angle 30º in the open kinetic chain at 0-day, three days, and after KT removed after three days  Significant ↓ in relative RE with KT during extension for target angle 30º in the close kinetic chain at 0-day, three days, and after KT removed after three days |
| KA Alahmari, RS Reddy, JS Tedla, PS Samuel, VN Kakaraparthi, K Rengaramanujam and I Ahmed [112]  Randomized controlled design | Mechanical neck pain  *Cervical* | KT: N=33  ?F, ?M  (22.7 ± 6.7)  PT: N=33  ?F, ?M  (23.1 ± 6.6) | KT: Kinesio tape  PT: Placebo tape | Absolute active RE during flexion  Absolute active RE during extension  Absolute active RE during left rotation  Absolute active RE during right rotation  Neck disability index | KT: Applied according to [113], “Y” and “I” strips used in two layers  Y-strip: Un-split end attached on the mid-thoracic region (0% tension), the neck was then flexed with chin touching the chest, split ends of Y strip applied on either ridge of the spine over cervical musculature (15-25% stretch)  I-strip 1: extended from T1-T2 to C1-C2  I-strip 2: applied perpendicularly over Y-strip, covered maximum cervical musculature to C3—C6 (full stretch)  KT was applied with both ends stretched, the middle portion used first and then pulled and released for applying ends without stretch  PT: Y-strip and I strips were applied without stretch, and the cervical spine was placed in neutral during PT application | Physical Therapist  *Thrice (at baseline, three, seven days)* | Pre-KT:  Post-KT: three days, seven days | Significant ↓ in RE during flexion between KT and PT during three and seven days follow up  Significant ↓ in RE during extension between KT and PT during three, seven days follow up  Significant ↓ in RE during left rotation between KT and PT during seven days follow up  Significant ↓ in RE during left rotation between KT and PT during three days follow up  Significant ↓ in neck disability index between KT and PT during three and seven days follow up |
| S Abbasi, M-R Hadian Rasanani, N Ghotbi, GR Olyaei, A Bozorgmehr and O Rasouli [114]  Randomized controlled design | Chronic low back pain  *Lumbar* | KT: N=15  9F, 6M  (44.3 ± 3.6)  PT: N=14  6F, 9M  (42.1 ± 6.9) | KT: Kinesio tape  PT: Placebo tape | Absolute active RE during flexion, extension at target angle 15º  Absolute active RE during flexion, extension at target angle 45º  Absolute active RE during flexion, extension at target angle 60º  Constant active RE during flexion, extension at target angle 15º  Constant active RE during flexion, extension at target angle 45º  Constant active RE during flexion, extension at target angle 60º  Oswestry disability index | KT: Applied according to [115], four “I” strips were applied in a sitting position in a star shape  Strip 1: Applied vertically  Strip 2: Applied horizontally  Strip 3, 4: applied at 45º to the vertical strip (15-25% stretch), over the point of maximum pain in the lumbar area  PT: Single I-strip of KT applied transversally on the maximum point of pain in the lumbar region | Physical Therapist  *Once* | Pre-KT  Post-KT: 3 days | No difference in absolute, constant RE at a target angle of 15º during flexion, extension between KT and PT  No difference in absolute, constant RE at target angle 45º during flexion, extension between KT and PT  No difference in absolute, constant RE at target angle 60º during flexion, extension between KT and PT |
| F Babakhani, M Heydarian and M Hatefi [116]  Randomized controlled design | Functional ankle instability  *Ankle* | KT: N=12  12M  (24.2 ± 90.2)  NT: N=12  12M  (25.2 ± 21)  KT wobble: N=12  12M  (25.1 ± 30.6)  Wobble: N=12  12M  (25 ± 8.8) | KT: Kinesio tape  NT: No tape | Absolute RE during plantarflexion for target angle 10º  Absolute RE during plantarflexion for target angle 30º | KT: Four tape strips used  Strip 1: applied to modify the function to limit plantar flexion and inversion, applied from outside of tibia and head of fibula when ankle placed in dorsiflexion and eversion (20% stretch) along the leg, end of strip applied on foot and in front of the outer ankle, then foot placed in plantar flexion, inversion and KT attached on left shin, the KT is rotated around the heel from outside of the foot in shape-of-six and placed on the outside and above the outer ankle (50% stretch)  Strip 2: applied above the inner ankle and wrapped around the heel (50% stretch) like a stirrup and attached outside the first strip above the outer ankle  Strip 3: applied from the middle of the arch of the foot (20% stretch) and comes cross-wide from both sides (50% stretch) in a figure-of-eight manner to the top of the ankle and stuck behind the shin  KT was applied from the beginning to the end of strips without pressure (0% stretch)  KT wobble: Kinesio taping with wobble board exercises | Not reported  *Once* | Pre-KT  Post-KT: - | Significant ↓ in RE with KT and KT wobble as compared to NT during plantarflexion for target angle 10º  Significant ↓ in RE with KT and KT wobble as compared to NT during plantarflexion for target angle 30º |
| S-Y Park, M-J Kim, S-E Seol, C Hwang, J-S Hong, H Kim and W-S Shin [117]  Randomized crossover design | Healthy  *Shoulder* | N= 20  8F, 12M  (23.4 ± 1.5) | DT: Dynamic tape  PT: Placebo tape  NT: No tape | Absolute active RE during flexion for target angle 50º  Absolute active RE during flexion for target angle 90º  Absolute active RE during flexion for target angle 110º | DT: Applied at two places to provide directional vector and resist forward movement of the humeral head, applied with gender consideration (female: 25cm, male: 30cm), applied in two steps  Step 1: DT applied on the arm extended forward on 2/3 part of biceps brachii, then with scapula raised, DT applied past humerus while covering the clavicle to the scapular body (150% stretch), end of the tape with no tension (0% stretch)  Step 2: with arm extended backwards, DT applied on 2/3^rd^ triceps brachii, then  with shoulder raised DT applied to the clavicle (150% stretch) through the spine of the scapula, the end of the tape with no tension (0% stretch)  PT: DT applied (0% stretch) on the distal part of the deltoid on the side above the clavicle in the sagittal plane | Not reported  *Once* | Pre-DT  Post-DT: - | Significant ↓ in RE with DT as compared to NT during flexion for target angle 50º  No difference in RE with DT as compared to NT during flexion for target angles 90º, 110º  No difference in RE with DT as compared to PT during flexion for target angles 50º, 90º, 110º |
| M Alawna and AA Mohamed [118]  Randomized controlled design | Chronic ankle instability  *Ankle* | ZnOT: N=33  15F, 18M  (44.3 ± 3.6)  PT: N=34  15F, 19M  (22.9 ± 3.2)  *Bandaging: N =33  19F, 14M  (23.5 ± 4.2) | ZnOT: Zinc oxide tape  PT: Placebo tape | Absolute active RE during dorsiflexion for target angle 10º  Absolute active RE for neutral position  Absolute active RE during plantarflexion for target angle 10º  Absolute active RE during dorsiflexion for target angle 20º | ZnOT: Non-adhesive gauze used on the front and back of the ankle, pre-wrap adhesive tape wrapped from the arch of the foot to the bottom of the calf muscle  ZnOT applied with three strips according to [61]  Strip 1 (anchor): applied circumferentially above the malleolus at the lower end of the shank  Strip 2 (stirrup): applied twice on the foot in the neutral position, ZnOT passed under the foot (medially), over the heel (posterior 1/3^rd^), laterally on the ankle  Strip 3 (locking): reinforced application to attach the ends of the stirrup to the anchor, applied circumferentially above the malleolus at the lower end of the shank  ZnOT was applied on the lateral side of the leg above the lateral malleolus while being aligned with the peroneus longus tendon  PT: Non-adhesive gauze is used on the front and back of the ankle, and pre-wrap adhesive tape is wrapped from the arch of the foot to the bottom of the calf muscle  ZnOT was applied on the lateral side of the leg above the lateral malleolus while being aligned with the peroneus longus tendon | Physical Therapist  *Four times (once every two weeks)* | Pre-ZnOT  Post-ZnOT: immediately after with ZnOT, two weeks after ZnOT removed, two months after ZnOT removed | Significant ↓ in RE with ZnOT as compared to PT during dorsiflexion for target angle 10º (two weeks, two months)  Significant ↓ in RE with ZnOT as compared to PT during neutral repositioning (two weeks, two months)  Significant ↓ in RE with ZnOT as compared to PT during plantarflexion for target angle 10º (two weeks, two months)  Significant ↓ in RE with ZnOT as compared to PT during plantarflexion for target angle 20º (two weeks, two months)  No differences in the RE with ZnOT as compared to PT immediately after application for all target angles |
| H Shahrokhi, H Miri and S Yekedehghan [119]  Randomized controlled design | Shoulder impingement syndrome  *Shoulder* | KT: N= 15  15F  (18 to 25)  NT: N= 15  15F  (18 to 25) | KT: Kinesio tape  NT: No tape | Absolute active RE during internal and external rotation for three different target angles | KT: Applied for upper trapezius and lower trapezius in two steps  Step 1: KT was applied from the outer 1/3^rd^ of the posterior edge of the clavicle to the occipital bone, then the subject bent the head to the opposite side and turned it towards the same side, and KT was applied on upper trapezius fibers (25% stretch), KT ends applied without tension (0% stretch)  Step 2: Applied on the lateral aspect of spinous processes from T7 to T12, then applied to the base of the scapula, then with the arm in 120º abduction KT applied with tension (70% stretch), then subject placed the arm in external rotation and end of KT applied with no pressure (0% stretch) | Not reported  *Once* | Pre-KT  Post-KT: - | Significant ↓ in RE with KT as compared to NT during internal and external rotation for three different target angles |
| Z Wei, X-X Wang and L Wang [120]  Randomized crossover design | Healthy  *Knee* | N= 35  35M  (22.5 ± 2.6) | KT-T: Kinesio tape with tension  KT: Kinesio tape with no tension  PT: Placebo tape  NT: No tape | Absolute RE during extension for target angle 30º | KT-T: Applied in three steps according to Kase’s technique [24] on rectus femoris, vastus medialis oblique, vastus lateralis (50% stretch)  Step 1: Applied from a point 10 cm below the anterior superior iliac spine and split in the form of “Y” above the  the patella, ending at the tibial tuberosity  Step 2: Applied from the middle 1/3^rd^ of the medial thigh to the medial edge of the patella  Step 3: Applied from the greater trochanter of the femur to the lateral edge of the patella  KT: Applied similarly to KT-T but without tension (0% stretch)  PT: KT applied without tension (0% stretch) 10 cm below the anterior superior iliac spine and 10cm upper edge of the patella | Not reported  *Once* | Pre-KT/KT-T  Post-KT/KT-T: immediately after with KT, 0.5 hours with KT, 1 hour with KT | No difference in RE between KT-T, KT, PT, and NT during extension for target angle 30º |
| B Rajabzadeh, A Amiri, B Vasaghi-Gharamaleki and SH Saneii [121]  Pretest-posttest quasi-experimental design | Healthy (sports personnel)  *Shoulder* | N= 30  30F  (24.7 ± 6.1) | KT: Kinesio tape | Absolute active RE during external rotation for target angle 30º  Absolute active RE during external rotation for target angle 60º | KT: Applied in three steps according to Kase’s technique [24]  Strip 1: I-strip applied on supraspinatus, KT placed 5cm below greater tuberosity humerus (0% stretch), stretch on supraspinatus instigated by abducting subject arm behind the trunk and moving neck to the opposite side with lateral flexion, then KT attached with slight tension on the skin along supraspinatus, the upper end of KT placed above scapular spine at the upper corner and inside the scapula  Strip 2: Y-strip applied on the deltoid, initially placed 5cm below deltoid tuberosity (0% stretch), to instigate stretch on anterior deltoid subject’s arm adducted 45º with some external rotation and anterior part of Y-strip placed on the skin to cover the anterior edge of the deltoid, for instigating stretch on posterior deltoid, shoulder adducted and flexed and rear part of Y-strip put on the skin to cover the posterior margin of the deltoid  Strip 3: I-strip applied to support strips used as strips 1 and 2, one end of strip 3 was placed on the coracoid process with the shoulder joint in slight internal rotation. When strip 3 reached the humerus mid-point arm flexed, and the posterior part of strip 3 was applied below the scapular spine, so that strip 3 passed over strips 1 and 2 and provided support | Not reported  *Once* | Pre-KT  Post-KT: 30 minutes | Significant ↓ in RE with KT during external rotation for a target angle of 60º  No difference in RE with KT during external rotation for a target angle of 30º |
| M Dhahi and MS Abdelsalam [48]  Randomized controlled design | Healthy (sports personnel with fatigue)  *Ankle* | KT: N=20  20M  (25.8 ± 3.7)  NT: N=20  20M  (24.4 ± 3.2) | KT: Kinesio tape  NT: No tape | Absolute active RE during inversion for target angle 10º  Absolute active RE during inversion for target angle maximum range-5º  Pre/post fatigue | KT: Applied according to Kase’s technique [24] from insertion to the origin to inhibit muscle activity  Strip 1 (anchor): application started without tension from the lateral side of the base of 1^st^ metatarsal and lateral side of cuneiform over to the lateral border of the foot, crossing the lateral side of the cuboid, then ankle placed in plantar flexion and inversion to increase tissue tension on lateral aspect, KT then applied behind the lateral malleolus (25% stretch) through peroneus longus up to fibular head | Physical Therapist  *Once* | Pre-KT  Post-KT: after fatigue protocol with KT | Significant ↓ in RE with KT as compared to NT during inversion for target angle 10º post-fatigue  No difference in RE with KT as compared to NT during inversion for target angle maximum-5º pre-fatigue  No difference in absolute RE in KT as compared to NT during inversion for target angle maximum-5º pre/post fatigue |
| I Narasinta, RH Masduchi and PM Kurniawati [47]  Pretest-posttest quasi experimental design | Osteoarthritis  *Knee* | N=8  7F, 1M  (59.3 ± 6.2) | KT: Kinesio tape | Absolute RE during extension at target angle 30º  Absolute RE during extension at target angle 45º  Absolute RE during extension at target angle 60º  TTDPM performed with angular velocity 1º/sec  Affected/un-affected side | KT: Applied with superior and inferior Y technique (25% stretch) | Not reported  *Once* | Pre-KT  Post-KT: 30 minutes | Significant ↓ in RE during extension with KT for a target angle of 30º on the affected side  No difference in RE during extension with KT for a target angle of 30º on the unaffected side  No difference in RE during extension with KT for a target angle of 45º on the un-affected side, affected side  No difference in RE during extension with KT for a target angle of 60º on the un-affected side, affected side  No difference in TTDPM with KT on the un-affected side, affected side |
| K Liu, J Qian, Q Gao and B Ruan [81]  Retrospective case series design | Anterior cruciate ligament rupture  *Knee* | N=48  48M  (26 ± 4.7) | KT: Kinesio tape | Absolute active RE during extension for target angle 30º  Lysholm scale | KT: Applied in 3 steps  Step 1: Y shape on both sides of the patella from the tibial tuberosity to the anterior inferior iliac spine (10% stretch) to induce detoning effect on the quadriceps  Step 2: Another Y shape KT applied from ischial tuberosity to medial tibial condyle and upper aspect of the fibular head (10% stretch) to induce a detoning effect on ischiocrural muscle  Step 3: KT applied over tibial tuberosity with dorsal tension (20% stretch) | Physical Therapist  *Once* | Pre-KT  Post-KT: immediately after with KT, seven days after with KT | Significant ↓ in RE with KT during extension for target angle 30º during the 0-day assessment  Significant ↓ in RE with KT during extension for target angle 30º after seven days  Significant ↑ in Lysholm scale score with KT during extension for target angle 30º after one day and seven days |
| CM Brogden, K Marrin, RM Page and M Greig [50]  Randomized crossover design | Healthy  *Ankle* | N=12  12M  (25 ± 5.0) | KT: Kinesio tape  RT: Rock tape  NT: No tape | Absolute active RE during inversion for target angle 15º  Absolute active RE during inversion for target angle maximum range-5º | KT: Applied according to Kase’s technique [122] and [104] with full stretch (i.e., 100%) was considered 40% of the overall tape length  From the anterior midfoot over tibialis anterior (115-120% stretch), attached distal to the anterior tibial tuberosity, middle 1/3^rd^ of KT (20% stretch), no tension applied on ends  Strip 2: applied proximally to the medial malleolus, wrapped around the heel like a stirrup, attached laterally to Strip 1  Strip 3: applied across the anterior ankle, covering lateral and medial malleolus  Strip 4: applied at the arch and stretched above medial and lateral malleolus (5 inches above)  25-min given for tape to gain adhesive strength  RT: Similar to KT | Physical Therapist  *Once* | Pre-KT/RT  Post-KT  Post-RT  Three days gap between each condition | Significant ↓ in RE with RT as compared to NT during inversion for target angle 15º  Significant ↓ in RE with RT as compared to NT during inversion for target angle maximum range-5º  No difference in RE with KT as compared to NT or RT during inversion for any target angle |
| NHN Allah, GA Mohamed, SM Elhafez and IM Emran [123]  Randomized controlled design | Sacroiliac joint dysfunction  *Hip* | ZnOT: N=15  15F  (37.3 ± 4.5)  NT: N=15  15F  (36.2 ± 3.1) | ZnOT: Zinc oxide tape | Absolute active RE during hip flexion for target angle 45º  Absolute active RE during hip abduction for target angle 15º | ZnOT: Hypo-allergic tape was applied first. After that, ZnOT (rigid) was used with the hip joint flexed to 45º and the femur in the neutral position  Strip 1: applied to restrain anterior iliac translation and pulled from the anterior superior iliac spine to the posterior superior iliac spine in a straight anterior-posterior direction  Strip 2: applied to restraint anterior iliac tilt and firmly pulled from the anterior superior iliac spine in an arching manner to the posterior superior iliac spine | Not reported  *Five times (once every two days)* | Pre-ZnOT  Post-ZnOT: 15 days | No difference in RE with ZnOT as compared to NT during flexion for a target angle of 45º  No difference in RE with ZnOT as compared to NT during abduction for a target angle of 15º |
| L Bischoff, C Babisch, J Babisch, F Layher, K Sander, G Matziolis, S Pietsch and E Röhner [82]  Crossover design | ACL rupture  *Knee* | N=48  9F, 39M  (33.1 ± 10.6) | KT: Kinesio tape | Absolute active RE  Lysholm scale  International knee documentation committee | KT: applied with detoning effect over quadriceps and toning effect over ischiocrural muscle  KT applied over tibial tuberosity with dorsal tension | Not reported  *Once* | Pre-KT  Post-KT: immediately after KT | Significant ↓ in RE with KT as compared to NT (uninjured leg)  Significant ↑ in Lysholm scale and International knee documentation committee score with KT |
| YF Shih, YF Lee and WY Chen [124]  Randomized controlled design | Shoulder impingement syndrome (sports personnel)  *Shoulder* | KT: N= 15  8F, 7M  (24.3 ± 2.8)  PT: N= 15  8F, 7M  (23.3 ± 3.3) | KT: Kinesio tape  PT: Placebo tape | Absolute active scapular RE during scapular upward/downward rotation  Absolute active scapular RE during scapular internal/external rotation  Absolute active scapular RE during scapular anterior/posterior tilt | KT: I-strip and Y-strip applied  Y-strip: Applied to encircle lower trapezius from origin to insertion with participants arm at 90º flexion with maximum scapular protraction  I-strip: Fully stretched applied perpendicular to muscle belly of the upper trapezius when scapula held in maximum retraction and depression  PT: 3M micropore tape applied (0% stretch) in a relaxed sitting position | Not reported  *Once* | Pre-KT  Post-KT: immediately after KT/PT | Significant ↓ in RE during scapular anterior/posterior tilt with KT as compared to PT  Significant ↓ in RE during scapular upward/downward rotation with KT as compared to PT  No difference in RE during scapular internal/external rotation between KT and PT |
| H Momeni-lari, M Ghasemi, K Khademi-kalantari and A Akbarzadeh-baghban [125]  Pretest-posttest quasi-experimental design | Functional ankle instability  *Ankle* | N= 20  20F  (27.7 ± 8.1) | KT: Kinesio tape | Absolute active RE during plantarflexion for target angle 30º | KT: Applied with two I-strips and one Y-strip with tendon correction technique  I-strip 1: Applied (50% stretch) from anterior tibialis anterior in plantar flexion and eversion from the middle of the leg to the tibial tuberosity  I-strip 2: Applied (50% stretch) for gastrocnemius in dorsiflexion from posterior ankle to knee joint  Y-strip 1: Applied (50% stretch) for peroneus in dorsiflexion and inversion from the outer ankle surface to the back of the head of the fibula | Not reported  *Once* | Pre-KT  Post-KT: immediately after KT | Significant ↓ in RE with KT during plantarflexion for target angle 30º |
| A Jahjah, D Seidenspinner, K Schüttler, A Klasan, TJ Heyse, D Malcherczyk and BF El-Zayat [49]  Randomized controlled design | Healthy (fatigue)  *Ankle* | LT: N=17  ?F, ?M  (26.7 ± 3.7)  NT: N=17  ?F, ?M  (26.8 ± 2.6) | LT: Leuko tape  NT: No tape | Absolute active RE during inversion for target angle 15º  Absolute active RE during inversion for target angle inversion-5º  Absolute passive RE during inversion for target angle 15º  Absolute passive RE during inversion for target angle inversion-5º  Variable active RE during inversion for target angle 15º  Variable active RE during inversion for target angle inversion-5º  Variable passive RE during inversion for target angle 15º  Variable passive RE during inversion for target angle inversion-5º  Pre/post fatigue | LT: Applied according to Macdonald’s method [126], pre-tape and LT are applied to support the lateral aspect of the ankle. The support provided by LT and its interface with anchors pull off the skin during foot activity intended to elicit the proprioceptive response | Not reported  *Once* | Pre-fatigue: With LT  Post-fatigue: after fatigue protocol with LT | No difference in absolute active RE during ankle inversion, pre/post fatigue for target angle 15º, inversion-5º  No difference in absolute passive RE during ankle inversion, pre/post fatigue for target angle 15º, inversion-5º  No difference in variable active RE during ankle inversion, pre-fatigue for target angle 15º, inversion-5º  No difference in variable passive RE during ankle inversion, pre-fatigue for target angle 15º, inversion-5º  Significant ↓ in absolute active RE during ankle inversion, post-fatigue for target angle inversion-5º  No difference in absolute active RE during ankle inversion, post-fatigue for target angle 15º  No difference in absolute passive RE during ankle inversion, post-fatigue for target angle 15º, inversion-5º  No difference in variable active RE during ankle inversion, pre/post fatigue for target angle 15º, inversion-5º  No difference in variable passive RE during ankle inversion, pre/post fatigue for target angle 15º, inversion-5º |
| SH Cho and HJ Moon [127]  Pretest-posttest quasi-experimental design | Healthy  *Ankle, knee* | Ankle KT: N= 14  9F, 5M  (22.5 ± 1.6) | KT: Kinesio tape | Proprioceptive index  X: degree of shaken to  the leftward and rightward  Y: degree  of shaken backward and forward  Rotation value: degree of turned left and right | KT: Applied on ankle with tape wrapped around the heel and stopped on lateral malleolus | Not reported  *-* | Pre-KT  Post-KT: immediately after KT | Significant ↓ in proprioceptive index (x-value) with KT ankle  No difference in proprioceptive index (y-value, rotation value) with KT ankle |
|  |  | Knee KT: N= 14  7F, 7M  (23.1 ± 1.3) |  |  | KT: Y-strip applied on femur with knee flexed |  |  | Significant ↓ in proprioceptive index (x-value) with KT knee  No difference in proprioceptive index (y-value, rotation value) with KT knee |
| KA Keenan, JS Akins, M Varnell, J Abt, M Lovalekar, S Lephart and TC Sell [128]  Randomized controlled design | Subacromial impingement syndrome  *Shoulder* | KT: N= 10  5F, 5M  (24.9 ± 5.1)  PT: N= 10  2F, 8M  (23.7 ± 3.1) | KT: Kinesio tape  PT: Placebo tape | TTDPM at 0.25º/ sec for shoulder internal rotation  TTDPM at 0.25º/ sec for shoulder external rotation | KT: Applied according to MD Thelen, JA Dauber and PD Stoneman [129] in two steps  Step 1: Applied with first Y-strip applied from insertion to origin of supraspinatus flowing the border of muscle belly, during the application subject laterally flexed cervical spine towards contralateral shoulder and internally rotated and extended the ipsilateral shoulder with hand placed on contralateral hip  Step 2: Applied from insertion of deltoid following anterior and posterior border of muscle belly, with anterior arm applied with shoulder externally rotated and horizontally abducted, posterior arm applied with shoulder internally rotated and horizontally adducted  PT: Cover-roll taping applied | Researcher  *-* | Pre-KT  Post-KT/PT: immediately after KT | No difference in TTDPM during shoulder internal rotation with KT as compared to PT  No difference in TTDPM during shoulder external rotation with KT as compared to PT |
|  | Healthy  *Shoulder* | KT: N= 10  7F, 3M  (25.7 ± 3.7) |  |  |  |  |  | No difference in TTDPM during shoulder internal rotation with KT  No difference in TTDPM during shoulder external rotation with KT |
| N Weerakkody and T Allen [130]  Randomized crossover design | Healthy (sports personnel with fatigue)  *Shoulder* | N= 14  14M  (26) | RT: Rigid tape  NT: No tape | Absolute active RE during flexion for target angle 45º  Absolute active RE during flexion for target angle 60º  Absolute active RE during flexion for target angle 90º  Pre/post fatigue | RT: Applied with fabric rigid strapping (50 mm) and with elastic adhesive (75 mm) bandage, RT applied with no tension (0% stretch) in four steps  Step 1: Initial position of the shoulder in 45º abduction, 1^st^ anchor strap applied over the shoulder (~3 cm) above the nipple over the top clavicle to the same level on the back  Step 2: Second  anchor applied around the torso extending perpendicular from the ends of the first anchor  Step 3: Third anchor applied around the upper arm at the lateral edge of the deltoid insertion  Step 4: Fourth and fifth supporting straps applied diagonally  in opposite directions from the shoulder anchor to the arm anchor  Step 5: Sixth supporting strap applied on the acromioclavicular joint to the lateral insertion of the deltoid  Step 6: Two final anchoring strips are applied on the first and third anchors to  lock in place the support straps, the elastic tape was placed over the anchors and supporting straps to complete the taping | Researcher  *Once* | Pre-AdT:  Post-AdT: 30 minutes after fatigue protocol with AdT | No difference in RE during flexion pre-fatigue for target angles 45º, 60º, 90º  Significant ↓ in RE with RT as compared to NT during flexion post-fatigue for a target angle of 45º  Significant ↓ in RE with RT as compared to NT during flexion post-fatigue for a target angle of 60º  No difference in RE between RT as compared to NT during flexion post-fatigue for a target angle of 90º |
| LM Wilson and M Greig [52]  Crossover design | Healthy (fatigue)  *Shoulder* | N=25  10F, 15M  (22) | KT: Kinesio tape  NT: No tape | Absolute active RE at 90-90 position during internal rotation for target angle 40º  Absolute active RE at 90-90 position during external rotation for target angle 60º  Absolute active RE at 90-90 position during mid-range for target angle 30º  Absolute active RE at the modified neutral position during internal rotation for target angle 40º  Absolute active RE at the modified neutral position during external rotation for target angle 60º  Absolute active RE at the modified neutral position during mid-range for target angle 30º  Absolute active RE at the diagonal position during internal rotation for target angle 0º  Absolute active RE at the diagonal position during external rotation for target angle 90º  Absolute active RE at the diagonal position during mid-range for target angle 45º  Pre/post maximal isometrics (fatigue) | KT: Applied along fascial lines whilst the fascia was on stretch, initial position with arm stretched applied in 2 steps  Step 1: Anterior strip of KT applied from the wrist travelling upwards across the forearm flexor muscles, Biceps Brachii, across the Pectoralis Major muscle finishing before the sternum (0% stretch)  Step 2: Posterior strip of KT applied similarly as step 1 from wrist upwards across the forearm extensor muscles, triceps brachii, posterior deltoid, upper fibres of trapezius, finishing at the lower cervical vertebrae (0% stretch) | Not reported  *Once* | Pre-KT:  Post-KT: after fatigue protocol with KT | Significant ↑ in RE with KT as compared to NT at 90-90 position during external rotation for target angle 60º pre-fatigue  No difference in RE with KT as compared to NT at 90-90 position during internal rotation for target angle 40º both pre/ post fatigue  No difference in RE with KT as compared to NT at 90-90 position during external rotation for target angle 60º post fatigue  No difference in RE with KT as compared to NT at 90-90 position during mid-range for target angle 30º both pre/ post fatigue  No difference in RE with KT as compared to NT at a modified neutral position during internal rotation for target angle 40º both pre/ post fatigue  No difference in RE with KT as compared to NT at a modified neutral position during external rotation for target angle 60º both pre/ post fatigue  No difference in RE with KT as compared to NT at a modified neutral position during mid-range for target angle 30º both pre/ post fatigue  No difference in RE with KT as compared to NT at a diagonal position during internal rotation for target angle 0º both pre/ post fatigue  No difference in RE with KT as compared to NT at a diagonal position during external rotation for target angle 90º both pre/ post fatigue  No difference in RE with KT as compared to NT at the diagonal position during mid-range for target angle 45º both pre/ post fatigue |
| D Bailey and P Firth [131]  Randomized crossover design | Healthy(sportspersonnel)  *Ankle* | KT: N=20  20M  (25.2 ± 5.8) | KT: Kinesio tape | Proprioceptive test accuracy score measured by the active center of pressure control | The authors did not explain the application procedure. Below is the application described by the reviewer based on the image representation in the manuscript  KT: Strip 1: applied from the head of metatarsals passing on posteriorly to the heel and extended till the gastrocnemius muscles  Strip 2: applied from the medial 2/3^rd^ of the tibia to the lateral 2/3^rd^ of the fibula, KT covered medial and lateral malleolus and passed over Strip 1 under the foot over the calcaneus | Not reported  *Once* | Pre-KT  Post-KT: immediately after KT | No difference in proprioceptive test accuracy score with KT |
| Y-S Bae [115]  Pretest-posttest quasi experimental design | Functional ankle instability  *Ankle* | N=35  21F, 14M  (21.3 ± 1.4) | ST: Spiral tape | Absolute active RE during plantarflexion at a target angle of 10º  Absolute active RE during plantarflexion at a target angle of 20º  Absolute active RE during inversion at a target angle of 10º  Absolute active RE during inversion at a target angle of 20º | ST: Applied in 3 locations  Location 1: 3 X 4 shape ST applied under, above and below medial malleolus, ST direction diagonal towards the right (30-35º) with 8mm interval  Location 2: 3 X 4 shape ST applied under, above and below lateral malleolus, ST direction diagonal towards the right (30-35º) with 8mm  Location 3: 3 X 4 shape ST applied under, above and below lateral talocrural joint, ST direction diagonal towards the right (30-35º) with 8mm | Not reported  *Once* | Pre-ST  Post-ST: immediately after ST | Significant ↓ in RE with ST during plantar flexion for target angle 10º  No difference in RE with ST during plantar flexion for a target angle of 20º  Significant ↓ in RE with ST during inversion for a target angle of 10º  Significant ↓ in RE with ST during inversion for a target angle of 10º |
| GLd Santos, MB Souza, K Desloovere and TL Russo [78]  Randomized crossover design | Stroke  *Shoulder* | N=13  3F, 10M  (59.4 ± 8.8) | KT: Kinesio tape  PT: Cremer tape | Absolute RE during abduction for a target angle of 30º  Absolute RE during abduction for a target angle of 60º  Absolute RE during flexion for target angle 30º  Absolute RE during flexion for a target angle of 60º  Paretic and non-paretic sides  Perceived subjective effects (improved perception, sensitivity, use of limb) | KT: with “paper-tension” (10-15% stretch), the acromioclavicular joint is considered as an initial anchor and one point immediately below deltoid insertion as the final anchor  Strip 1: applied on the anterior portion of the deltoid with the shoulder in 30º passive extension  Strip 2: applied on the middle portion of the deltoid with the shoulder in 30º passive horizontal adduction  Step 3: applied on posterior deltoid with shoulder placed in 90º  Passive flexion  PT: Similar to KT | Physical Therapist  *Twice* | Pre-KT/PT  Post-KT  Post-PT  1-month gap between each condition | Paretic side:  Significant ↓ in RE with KT as compared to PT during abduction for target angles 30º, 60º (0-day)  Significant ↓ in RE with PT as compared to KT during abduction for target angles 30º, 60º (0-day)  Significant ↓ in RE with KT as compared to PT during flexion for target angles 30º, 60º (0-day)  Significant ↓ in RE with PT as compared to KT during flexion for target angles 30º, 60º (0-day)  Non-paretic side:  No difference in RE with KT as compared to PT  during abduction for target angles 30º, 60º (0-day, 1-month)  No difference in RE with KT as compared to PT  during flexion for target angle 30º, 60º (0-day, 1-month)  No difference in the perceived effects with KT as compared to PT |
| Z Long, R Wang, J Han, G Waddington, R Adams and J Anson [84]  Randomized crossover design | Healthy  *Ankle* | N=24  12F, 12M  (22 ± 1.5) | KT: Kinesio tape  AT: Athletic tape  NT: No tape | Proprioceptive differentiation scores with AMEDA during full weight bearing during ankle inversion for a target angle of 10º  Proprioceptive differentiation scores with AMEDA during full weight bearing during ankle inversion for a target angle of 12º  Proprioceptive differentiation scores with AMEDA during full weight bearing during ankle inversion for a target angle of 14º  Proprioceptive differentiation scores with AMEDA during full weight bearing during ankle inversion for a target angle of 16º  Perceived comfort, support, and proprioceptive performance | KT: Applied according to Kase’s technique [102]  AT: Applied with a figure-of-eight approach followed by a heel lock and a stirrup to the heel | Physical Therapist  *Once* | Pre-KT/AT  Post-KT/AT: immediately after KT/AT | No difference in proprioceptive differentiation scores between KT, AT and NT during ankle inversion for all target angles  Significantly ↑ comfort and performance with KT as compared to AT |
| SA Ruggiero, LR Frost, LA Vallis and SHM Brown [59]  Non-randomized controlled design | Healthy  *Lumbar, thoracic* | KT: N= 12  12F  (20 to 24)  PT: N= 12  12F  (20 to 24) | KT: Kinesio tape with tension  PT: Placebo tape tape | Absolute active RE during lumbar flexion for a target angle of 45º  Absolute active RE during lumbar flexion for the neutral position  Absolute active RE during thoracic flexion for target angle 45º  Absolute active RE during thoracic flexion for a neutral position | KT: Applied horizontally at L1-L2 vertebrae (80% stretch)  PT: KT applied horizontally at L1-L2 vertebrae (0% stretch) | Not reported  *Once* | Pre-KT  Post-KT: after 30 minutes with KT, after 30 minutes without KT | No difference in RE during lumbar flexion for a target angle of 45º with KT and PT  No difference in RE during the lumbar neutral position with KT and PT  Significant ↑ in RE during lumbar flexion for a target angle of 45º with KT and PT  No difference in RE during the lumbar neutral position with KT and PT removed  No difference in RE during thoracic flexion for a target angle of 45º with KT and PT  No difference in RE during thoracic flexion for a target angle of 45º with KT and PT removed  Significant ↑ in RE during thoracic neutral position with KT and PT  Significant ↑ in RE during thoracic neutral position with KT and PT  removed |
| R Torres, R Trindade and RS Gonçalves [132]  Randomized controlled design | Healthy  *Knee* | KT: N= 20  10F, 10M  (20.9 ± 1.3)  NT: N= 10  5F, 5M  (21.8 ± 1.1) | KT: Kinesio tape  NT: No tape | Absolute active RE during flexion for target angle 30º  Absolute active RE during flexion for target angle 60º  Absolute passive RE during flexion for target angle 30º  Absolute passive RE during flexion for target angle 60º  TTDPM during flexion for target angle 30º with angular velocity 0.25º/sec  TTDPM during flexion for target angle 60º with angular velocity 0.25º/sec | KT: Applied according to Kase’s technique [133], with hip in the neutral position and knee flexed, Y-strip applied from the anterior inferior iliac spine to the anterior tibial tuberosity to stimulate sensory mechanoreceptors on quadriceps, KT applied with tension in the middle (50-75% stretch) and no tension (0% stretch) on ends of the KT | Physical Therapist  *Once* | Pre-KT  Post-KT: immediately after KT, one day after without KT | No difference in active RE with KT as compared to NT during flexion for target angles 30º and 60º at 0-day and one day after application  No difference in passive RE with KT as compared to NT during flexion for target angles 30º and 60º at 0-day and one day after application  Significant ↓ in TTDPM with KT as compared to PT during flexion for target angle 30º with angular velocity 0.25º/sec at 0-day and one day after application  Significant ↓ in TTDPM with KT as compared to PT during flexion for target angle 60º with angular velocity 0.25º/sec at 0-day and one day after application |
| EE Kurt, Ö Büyükturan, HR Erdem, F Tuncay and H Sezgin [134]  Randomized controlled design | Patellofemoral pain syndrome  *Knee* | KT: N=44  25F, 19M  (31.6 ± 6.9)  PT: N=40  24F, 16M  (30.9 ± 7.2) | KT: Kinesio tape  PT: Placebo tape | Absolute passive RE during extension for target angle 60º  Tampa Kinesiophobia scale | KT: Applied according to Kase’s technique [24] for vastus medialis oblique muscle facilitation with patellar correction strip, initial position knee flexed at 90º, applied in two steps  Step 1: Y-strip’s unsplit end applied on quadriceps, then anchor applied at mid-thigh (0% stretch), then each unsplit end applied bracketing and terminating below the patella, mid-section of Y-strip applied under tension and ends without  Step 2: Patellar strip applied with medium tension alongside knee cap  PT: 2 KT strips applied horizontally with knee straight  Strip 1: Applied 7 cm above superior patellar border (0% stretch)  Strip 2: Applied 7cm below inferior patellar border (0% stretch) | Physical Therapist  *Twice for 22 participants as KT lost adhesiveness* | Pre-KT  Post-KT: 2 days after KT | Significant ↓ in RE with KT as compared to PT during extension  Significant ↓ in Tampa Kinesiophobia scale with KT as compared to PT during extension |
| H-D Seo, M-Y Kim, J-E Choi, G-H Lim, S-I Jung, S-H Park, S-H Cheon and H-Y Lee [135]  Pretest-posttest quasi-experimental design | Healthy  *Ankle* | N=26  ?F, ?M  (21.2 ± 0.8) | KT: Kinesio tape | Absolute RE during dorsiflexion  Absolute RE during plantarflexion  Absolute RE during inversion  Absolute RE during eversion | KT: Applied with ankle placed in the inverted position, KT applied from calcaneus to talus, another KT applied over the previous strip at 90º, and 20 cm above the lateral and medial malleolus, the middle section of KT passed under the center of the sole, so KT applied over the top of the foot while covering the floor from toes to the Achilles tendon in a figure-of-eight shape | Not reported  *Once* | Pre-KT  Post-KT: immediately after KT | Significant ↓ in RE with KT during dorsiflexion  No difference in RE with KT during plantarflexion  Significant ↓ in RE with KT during inversion  No difference in RE with KT during eversion |
| M Akbari, G Pahnabi and H Karimi [136]  Pretest-posttest quasi-experimental design | Post-ACL reconstruction (sports personnel)  *Knee* | N= 20  20M  (24.4 ± 1.4) | KT: Kinesio tape | Absolute active RE from flexion to extension for a target angle of 15º  Absolute active RE from flexion to extension for a target angle of 30º  Absolute active RE from flexion to extension for a target angle of 60º  Absolute active RE from extension to flexion for a target angle of 15º  Absolute active RE from extension to flexion for a target angle of 30º  Absolute active RE from extension to flexion for a target angle of 60º  Closed kinetic chain/open kinetic chain | KT: Knee in 90º flexion KT applied from tibial tuberosity to the lower third of the thigh in the anteromedial and anterolateral position of knee flexed by 30º (60% stretch), distal and proximal bases of the KT without tension | Not reported  *Once* | Pre-KT  Post-KT: immediately after KT | Significant ↓ in RE with KT from flexion to extension for target angles 15º, 30º, 60º (both weight-bearing/non-weight bearing)  Significant ↓ in RE with KT from extension to flexion for target angles 15º, 30º, 60º (both weight-bearing/non-weight bearing) |
| IK Ahn, YL Kim, Y-H Bae and SM Lee [137]  Randomized controlled design | Healthy fatigue  *Knee* | KT: N= 15  15F  (29 ± 2.8)  PT: N= 15  15F  (31.3 ± 4.1)  NT: N= 15  15F  (30.2 ± 3.4) | KT: Kinesio tape  PT: Placebo tape  NT: No tape | Absolute active RE for a target angle of 135º  Variable active RE for a target angle of 135º | KT: Applied on quadriceps (40% stretch)  Rectus femoris: applied from ASIS to superior border of patella during full knee flexion  Vastus medialis: applied from lower part of intertrochanteric line to medial superior aspect of patella during full knee flexion  Vastus lateralis: applied from greater trochanter of femur to lateral superior aspect of patella during full knee flexion  PT: Y-strip applied from muscle belly of quadriceps (0% stretch) below ASIS to level of patella | PT  Once | Pre-KT  Post-KT: immediately after KT | No difference in absolute active RE with KT as compared to PT or NT for a target angle of 135º  No difference in variable active RE with KT as compared to PT or NT for a target angle of 135º |
| Z Barzegar Ganji, F Dehghan-Manshadi, K Khademi-Kalantari, M Ghasemi and SM Tabatabaee [138]  Non-randomized controlled design | Shoulder impingement syndrome  *Shoulder* | N=12  12M  (50.7 ± 8.4) | KT: Kinesio tape  NT: No tape | Absolute RE during abduction at a target angle of 90º  Absolute RE during abduction at target angle half range of motion  Absolute RE during abduction at target angle maximum range-10º | KT: Explanation provided by the reviewer based on the image representation in the manuscript  KT applied on deltoid, trapezius, pectoralis major, middle trapezius fibers, supraspinatus | Not reported  *Once* | Pre-KT  Post-KT: immediately after KT | Significant ↓ in RE with KT as compared to NT during abduction at a target angle of 90º  Significant ↓ in RE with KT as compared to NT during abduction’s half range of motion  Significant ↓ in RE with KT as compared to NT during abduction at target maximum range-10º |
| LM Aarseth, DN Suprak, GR Chalmers, L Lyon and DT Dahlquist [60]  Randomized cross over design | Healthy (sports personnel)  *Shoulder* | N=27  16F, 11M  (20.4 ± 1.0) | KT: Kinesio tape  NT: No tape | Absolute RE during shoulder elevation (scapular plane: 35º anterior to the coronal plane) at a target angle of 50º  Absolute RE during shoulder elevation (scapular plane: 35º anterior to the coronal plane) at a target angle of 90º  Absolute RE during shoulder elevation (scapular plane: 35º anterior to the coronal plane) at a target angle of 110º  Variable RE during shoulder elevation (scapular plane: 35º anterior to the coronal plane) at a target angle of 50º  Variable RE during shoulder elevation (scapular plane: 35º anterior to the coronal plane) at a target angle of 90º  Variable RE during shoulder elevation (scapular plane: 35º anterior to the coronal plane) at a target angle of 110º | KT: application included one “Y” strip and two “I” strips  The authors did not explain the application procedure. Below is the application described by the reviewer based on the image representation in the manuscript  Y-strip: Unsplit part applied on the insertion of the deltoid and the anterior split end reached the coracoid process, and the posterior split end reached the junction of the scapular spine and acromion  I-strip 1: applied to join the two split ends of the Y-strip over the superior aspect of the shoulder joint  I-strip 2: applied from the acromion process to the lateral distal 2/3^rd^ of the humerus | Not reported  *Once* | Pre-KT  Post-KT: immediately after KT | Significant ↑ in absolute RE with KT as compared to NT during shoulder elevation at a target angle of 90º  No difference in absolute RE with KT as compared to NT during shoulder elevation at target angles 50º, 110º  No difference in variable RE with KT as compared to NT during shoulder elevation at target angles 50º, 90º, 110º |
| H-y Cho, E-H Kim, J Kim and YW Yoon [79]  Randomized controlled design | Osteoarthritis  *Knee* | KT: N=23  17F, 6M  (58.2 ± 4.5)  PT: N=24  16F, 7M  (57.5 ± 4.4) | KT: Kinesio tape  PT: Placebo tape | Absolute active RE during flexion for a target angle of 15°  Absolute active RE during flexion for a target angle of 30°  Absolute active RE during flexion for a target angle of 45 | KT: Applied to quadriceps with proper tension according to Kase’s technique [102], KT applied in two steps  Step 1: I-strip applied with hip extended and knee flexed at 60º. I-strip started at the origin of rectus femoris  Step 2: Y-strip applied proximal to the superior patellar border, no tension (0% stretch) on both ends, the portion between the anchor and superior patella applied with pressure (15-25% stretch)  PT: Applied similarly to KT without tension on rectus femoris, with knee not in flexion | Physical Therapist  *Once* | Pre-KT  Post-KT: immediately after KT | Significant ↓ in RE with KT as compared to PT during flexion at a target angle of 15°  Significant ↓ in RE with KT as compared to PT during flexion at a target angle of 15°  Significant ↓ in RE with KT as compared to PT during flexion at a target angle of 15° |
| SM Burfeind and N Chimera [139]  Randomized controlled design | Healthy  *Shoulder* | KT: N=8  3F, 5M  (24.3 ± 3.9)  NT: N=8  4F, 4M  (23.3 ± 1.3) | KT-spider: Kinesio tape with spider tech  NT: No tape | Absolute active RE during flexion at target angle maximum range-10%  Absolute active RE during extension at target angle maximum range-10%  Absolute active RE during external rotation at target angle maximum range-10%  Absolute active RE during internal rotation at target angle maximum range-10% | KT: pre-cut tape applied in six sections  Section 1: With the arm resting at the side, applied to the shoulder at long the junction of the upper arm and shoulder at mid-deltoid  Section 2: With the arm placed behind the back, applied along the top of the shoulder blade (with gentle stretch)  Section 3: applied into the back of the shoulder (without stretch)  posterior shoulder border of strip 1  Step 4: arm moved to across the front of the body (i.e., hand laced on the opposite shoulder), applied (no stretch) direct to the back border of section 1  Section 5: arm extended straight behind the body, applied on the lateral portion of biceps (no stretch)  Section 6: applied on biceps (no stretch)  Section 7: with the arm extended forward and elbow straight at the height of the shoulder, applied on the skin along the triceps muscle (no stretch)  Section 8: with the arm pointing out in front and elbow bent at the height of the shoulder, applied on triceps (no stretch)  KT was rubbed to activate the glue | Athletic trainer  *Once* | Pre-KT-spider  Post-KT: immediately after KT | Significant ↓ in RE with KT as compared to NT during flexion at target angle maximum range-10%  No difference in RE with KT as compared to NT during extension at target angle maximum range-10%  Significant ↓ in RE with KT as compared to NT during external rotation at target angle maximum range-10%  No difference in RE with KT as compared to NT during internal rotation at target angle maximum range-10% |
| S Hosp, G Bottoni, D Heinrich, P Kofler, M Hasler and W Nachbauer [140]  Randomized crossover design | Healthy (fatigue)  *Knee* | N=12  12F  (23.6 ± 2) | KT: Kinesio tape  NT: No tape | Absolute RE for a random target angle between 20º and 70º  Pre/post fatigue | KT: Applied according to Kase’s technique [24] with initial position knee flexed at 90º  Strip 1: Applied on vastus lateralis from 10cm below the greater trochanter major to the lateral edge of the patella  Strip 2: Applied as an anchor around the patella, initial 3cm (no stretch), area between the anchor and the superior patella was stretched (75% stretch), tape around patella stretched (120%), both tape ends (no stretch) and intersected  Strip 3: Applied over the patella with maximal tension (10cm) | Not reported  *Once* | Pre-KT  Post-KT: after 30 minutes of uphill walking with KT | No difference in absolute RE between KT and NT at pre-fatigue  No difference in absolute RE between KT and NT at post-fatigue |
| GG Zanca, SM Mattiello and AR Karduna [141]  Randomized crossover design | Healthy (fatigue)  *Shoulder* | N= 24  12F, 12M  (21.5 ± 2.7) | KT: Kinesio tape  PT: Placebo tape  NT: No tape | Absolute active RE during elevation for a target angle of 50º  Absolute active RE during elevation for a target angle of 70º  Absolute active RE during elevation for a target angle of 90º | KT: Applied on the deltoid muscle from origin to insertion in three steps according to Kase’s technique [24]  Step 1: Base of KT applied withy no pressure over the acromioclavicular joint with arm at the side of trunk  Step 2: Shoulder placed in abduction, external rotation and horizontal abduction and tape applied along anterior deltoid over deltoid tuberosity  Step 3: Posterior strip applied with shoulder moved into horizontal adduction with internal rotation while maintaining around 90º abduction  PT: KT applied similarly but arm remained on the side with no pressure | Not reported  - | Pre-KT  Post-KT: Immediately after KT, after fatigue protocol | No difference in absolute RE between KT and NT during elevation for a target angle of 50º after KT and after fatigue protocol  No difference in absolute RE between KT and NT during elevation for a target angle of 70º after KT and after fatigue protocol  No difference in absolute RE between KT and NT during elevation for a target angle of 90º after KT and after fatigue protocol |
| DM Hopper, TL Grisbrook, M Finucane and K Nosaka [142]  Randomized crossover design | Healthy  *Ankle* | N= 20  20F  (22.9 ± 3.9) | RT: Rigid tape  NT: No tape | Absolute active RE during plantarflexion at a target angle of 10º  Absolute active RE during plantarflexion at a target angle of 15º  Absolute active RE during plantarflexion at a target angle of 20º  Absolute passive RE during plantarflexion at a target angle of 10º  Absolute passive RE during plantarflexion at a target angle of 15º  Absolute passive RE during plantarflexion at a target angle of 20º | RT: Applied on the ankle joint to support medial and lateral ligament complex while allowing complete range of motion with hindfoot taping technique from DM Hopper, P McNair and BC Elliott [143]  RT applied in three steps  Step 1: two stirrups applied  Step 2: stirrups were followed by two half eights  Step 3: RT finished with a horizontal locking tape | Physical therapist  - | Pre-RT  Post-RT: immediately after RT | No difference in active RE with RT during plantarflexion at a target angle of 10º as compared to NT  No difference in active RE with RT during plantarflexion at a target angle of 15º as compared to NT  No difference in active RE with RT during plantarflexion at a target angle of 20º as compared to NT  No difference in passive RE with RT during plantarflexion at a target angle of 10º as compared to NT  No difference in passive RE with RT during plantarflexion at a target angle of 15º as compared to NT  No difference in passive RE with RT during plantarflexion at a target angle of 20º as compared to NT |
| I Miralles, S Monterde, O del Rio, S Valero, S Montull and I Salvat [61]  Randomized controlled design | Healthy  *Ankle* | KT: N=35  20F, 15M  (22.7)  NT: 33  20F, 13M  (22.5) | KT: Kinesio tape  NT: No tape | Absolute active RE during neutral position  Absolute active RE during dorsiflexion at a target angle of 5º  Absolute active RE during dorsiflexion at a target angle of 10º  Absolute active RE during plantarflexion at a target angle of 5º  Absolute active RE during plantarflexion at a target angle of 10º  Absolute active RE during inversion at a target angle of 5º  Absolute active RE during eversion at a target angle of 5º | KT: Applied according to Kase’s technique [24], a single strip was applied over the anterior talofibular ligament from distal to proximal (50% stretch) direction, proximal and distal ends used with no tension (0% stretch), a similar procedure was repeated on the calcaneofibular ligament and posterior talofibular ligament | Not reported  *Once* | Pre-KT  Post-KT: immediately after KT, after two days without KT | No difference in RE with KT as compared to NT during neutral position at time points 0-day, two days  No difference in RE with KT as compared to NT during dorsiflexion for target angles 5º and 10º at time points 0-day, two days  No difference in RE with KT as compared to NT during plantarflexion for target angle 5º at time points 0-day, two days  No difference in RE with KT as compared to NT during plantarflexion for a target angle of 10º at time points 0-day  Significant ↑ in RE with KT as compared to NT during plantarflexion for target angle 10º at time points 2 days  No difference in RE with KT as compared to NT during inversion for target angles 5º at time points 0-day, two days  No difference in RE with KT as compared to NT during eversion for target angles 5º at time points 0-day, two days |
| M Barbanera, FdA Mazuchi, JPB Batista, JdM Ultremare, JdS Iwashita and UF Ervilha [144]  Crossover design | Healthy  *Ankle* | N=16  16F  (20.8 ± 2.3) | IT: Impermeable tape  NT: No tape | Absolute active RE during inversion at target angle 10º  Absolute active RE during inversion at target angle 25º  Absolute active RE during plantarflexion at a target angle of 15º  Absolute active RE during plantarflexion at a target angle of 30º  Variable active RE during inversion at target angle 10º  Variable active RE during inversion at target angle 25º  Variable active RE during plantarflexion at a target angle of 15º  Variable active RE during plantarflexion at a target angle of 30º | IT: No explanation of the application procedure was provided by the authors. Below is the application described by the reviewer based on the image representation in the manuscript  IT covered metatarsals, also applied on the medial malleolus and the base of the foot (i.e., calcaneum). IT wrapped around the lower 1/3^rd^ of the tibia and fibula | Not reported  *Once* | Pre-IT  Post-IT: after 15 minutes with IT | No difference in absolute RE with IT as compared to NT during inversion at target angles 10º, 25º  No difference in absolute RE with IT as compared to NT during plantarflexion at target angles 15º, 30º  No difference in variable RE with IT as compared to NT during inversion at target angles 10º, 25º  Significant ↓ in variable RE with IT as compared to NT during plantarflexion at a target angle of 15º  No difference in variable RE with IT as compared to NT during plantarflexion at a target angle of 30º |
| G Fratocchi, F Di Mattia, R Rossi, M Mangone, V Santilli and M Paoloni [145]  Randomized crossover design | Healthy  *Elbow* | N= 20  3F, 17M  (23.6 ± 2.3) | KT: Kinesio tape  PT: Placebo tape  NT: No tape | % of exact trials during passive joint position sense during elbow flexion for target angles for target angles of 20º, 50º, 80º, 110º, 140º | KT: Applied over the length of biceps muscle, starting from the biceps muscle from insertion (distally) to origin while embracing sides with tension (75% stretch), ends of I-strips applied with no pressure  PT: Two I-strips applied without tension in transverse section across proximal and distal portion of the biceps muscle belly | Not reported  - | Pre-KT  Post-KT/PT: immediately after KT | No significant difference in the % of exact trials during passive joint position sense with KT as compared to PT and NT during elbow flexion for target angles for target angles of 20º, 50º, 80º, 110º, 140º |
| H Niknam, A Sarmadi, M Salavati and F Madadi [146]  Pretest posttest quasi experimental design | Anterior cruciate ligament reconstruction  *Knee* | N=20  20M  (27 ± 5.5) | KT: Kinesio tape | Absolute RE during squat for target angle 30º  Absolute RE during squat for target angle 60º | - | Not reported  - | Pre-KT  Post-KT: immediately after with KT, after 10 days with KT | Significant ↓ in RE during squat for target angles 30º, 60º at 0-day, 10 days |
| Jj Lin, CJ Hung and PL Yang [147]  Randomized crossover design | Healthy  *Shoulder* | N=10  10M  (23.7 ± 4.8) | KT: Kinesio tape  NT: No tape | Active proprioceptive feedback index angle error for the magnitude of the end range tasks  Active proprioceptive feedback index angle error for the magnitude of mid-range tasks  Active proprioceptive feedback index for similarity index of end  range tasks  Active proprioceptive feedback index for similarity index of mid-range tasks | KT: Applied according to the modified method of JS Lewis, C Wright and A Green [148], I-strip applied with the subject fully retracting and depressing their scapula, I-strip applied from the inferior margin of medial 1/3^rd^ of the clavicle to T12 with full tension | Not reported  *Once* | Pre-KT  Post-KT: immediately after KT | Significant ↓ proprioceptive feedback index for KT as compared to NT for end-range task  Significant ↓ proprioceptive feedback index for KT as compared to NT for mid-range task  No difference in the proprioceptive feedback index for the similarity index of mid-range and end-range tasks |
| A Aytar, N Ozunlu, O Surenkok, G Baltacı, P Oztop and M Karatas [149]  Randomized controlled design | Patellofemoral pain syndrome  *Knee* | KT: N= 12  12F  (22.4 ± 1.6)  PT: N= 10  10F  (26.2 ± 3.5) | KT: Kinesio tape  PT: No tape | Absolute passive RE during extension for target angle 45º | KT: Applied according to Kase’s technique [24], two Y-strips were applied over the quadriceps in two steps:  Step 1: KT applied approximately on mid-thigh over vastus medialis with thigh in 45º knee flexion with paper-off tension (0% stretch) applied until Y part of KT reached the superior pole of the patella, then the tail of Y-strip applied with pressure (50%-75% stretch) for correction below tibial tuberosity with no initial tension, then the tail of Y-strips was applied without stress in the medial and lateral border of patella including vastus medialis and lateralis  Step 2: two mechanical patellar correction strips with two I-strips applied around the patella with tension (50%-75% stretch) with the knee in 45º flexion  PT: Applied similarly to KT but with sticking plaster without tension | Physical therapist  *Once* | Pre-KT  Post-KT: immediately after KT | No difference in RE with KT as compared to PT during extension for a target angle of 45º |
| W-H Lee, O-Y Kwon, C-H Yi, H-S Jeon and S-M Ha [150]  Crossover design | Healthy  *Wrist* | N= 15  ?F, ?M  (42 ± 5.2) | ZnOT: Zinc oxide tape  NT: No tape | Absolute active RE during wrist extension for target angle 20º, 25º, 30º | ZnOT: Applied with the wrist extended to contract with extensor carpi radialis brevis, then tape applied on the proximal forearm starting medially and tracking laterally, the process repeated twice or thrice  The tape tightened as per the subject’s tolerability and was snug during the contraction of wrist extensors | Physical therapist  *Once* | Pre-ZnOT  Post-ZnOT: Immediately after ZnOT | Significant ↓ in RE with ZnOT as compared to NT during extension for target angles 20º, 25º, 30º  No difference in RE with ZnOT as compared to NT during extension for target angles 20º, 25º, 30º |
|  | Lateral epicondylitis  *Wrist* | N= 15  ?F, ?M  (41.9 ± 6.8) |  |  |  |  |  |  |
| M Iris, S Monterde, M Salvador, I Salvat, J Fernández-Ballart and B Judith [151]  Randomized controlled design | Healthy  *Ankle* | LT: N= 18  ?F, ?M  (23.2 ± 4.2)  NT: N=15  ?F, ?M  (23.2 ± 4.2) | LT: Leuko tape  NT: No tape | Absolute active RE during dorsiflexion at target angle 10º  Absolute active RE during neutral position  Variable active RE during plantarflexion at target angle 10º  Variable active RE during plantarflexion at target angle 20º | LT: Pre-tape applied before LT, then two proximal anchorage strips applied below the largest part of the calf muscles, then one or two distal anchorage strips applied according to the foot, then three active strips applied from internal to external in a fan distribution, proximal strips were then closed, then two eight shaped strips were applied to restrict ankle inversion, and one eight shape strip was finished at the anterior dorsal part of the foot, lastly closing strips were applied | Physical Therapist  *Once* | Pre-LT  Post-LT: 2 days after LT | Significant ↓ in RE with LT as compared to NT during dorsiflexion at a target angle of 10º  Significant ↓ in RE with LT as compared to NT during neutral position  No difference in RE with LT as compared to NT during plantarflexion at target angles 10º, 30º |
| T Bradley, C Baldwick, D Fischer and GAC Murrell [152]  Randomized crossover design | Healthy (sports personnel)  *Shoulder* | N= 33  33M  (22 ± 3.6) | LT: Leuko tape  NT: No tape | Absolute active RE during flexion at a target angle of 30º  Absolute active RE during abduction at a target angle of 90º  Absolute active RE during external rotation at a target angle of 90º | LT: Applied with an elastic overwrap with three anchors and three support strips  Anchor 1: applied from 3cm above the nipple over the top of the clavicle and to the same level of back  Anchor 2: Applied perpendicularly from the ends of the first anchor around the torso below the axilla  Anchor 3: Applied around the upper arm at the lateral edge of the deltoid insertion  Strip 1: Applied over the shoulder anchor to the anchor of the arm, halfway between the shoulder and on the lateral insertion of the deltoid  Strip 2: Applied on acromion-clavicular joint following the anterior edge of trapezius from over the shoulder anchor to the same point as the lateral deltoid insertion  Strip 3: Applied from 1/3^rd^ distance between the second strip and second anchor to the same point of lateral deltoid insertion along the scapular spine  All strips were applied with no tension (0% stretch), two final anchor strips were applied over the first anchor to lock the tape in place, and then elastic overwrap was placed on the strips and anchors | Physical Therapist  *Once* | Pre-LT  Post-LT: immediately after with LT | No difference in RE with LT as compared to NT during flexion at a target angle of 30º  No difference in RE with LT as compared to NT during abduction at a target angle of 90º  No difference in RE with LT as compared to NT during external rotation at a target angle of 90º |
| KM Refshauge, J Raymond, SL Kilbreath, L Pengel and I Heijnen [153]  Randomized crossover design | Ankle sprain  *Ankle* | N=16  14F, 2M  (22 ± 3) | LT: Leuko tape  NT: No tape | TTDPM (70%) during inversion with angular velocity 0.1º/sec  TTDPM (70%) during inversion with angular velocity 0.5º/sec  TTDPM (70%) during inversion with angular velocity 2.5º/sec  TTDPM (70%) during eversion with angular velocity 0.1º/sec  TTDPM (70%) during eversion with angular velocity 0.5º/sec  TTDPM (70%) during eversion with angular velocity 2.5º/sec | LT: Applied using a standard inversion taping technique with a combination of heel locks, figure of six, and stirrups | Not reported  *Once* | Pre-LT  Post-LT: immediately after with LT | Significant ↑ in TTDPM during eversion perception with LT as compared to NT for angular velocities 0.5º/sec  No difference in TTDPM during inversion perception with LT as compared to NT for angular velocities 0.1º/sec  Significant ↑ in TTDPM during inversion perception with LT as compared to NT for angular velocities 0.5º/sec  No difference in TTDPM during inversion perception with LT as compared to NT for angular velocities 2.5º/sec  No difference in TTDPM during eversion perception with LT as compared to NT for angular velocities 0.1º/sec  No difference in TTDPM during eversion perception with LT as compared to NT for angular velocities 2.5º/sec |
| MJ Callaghan, J Selfe, A McHenry and JA Oldham [53]  Randomized crossover design | Patellofemoral pain syndrome  *Knee* | N= 32  14F, 18M  (31.9 ± 11.2) | PatellarT: Patellar taping  NT: No tape | Absolute active RE during extension for a target angle of 60º  Absolute active RE during extension for a target angle of 20º  Absolute passive RE during extension for a target angle of 60º  Absolute passive RE during extension for a target angle of 20º  Variable active RE during extension for a target angle of 60º  Variable active RE during extension for a target angle of 20º  Variable passive RE during extension for a target angle of 60º  Variable passive RE during extension for a target angle of 20º  Relative active RE during extension for a target angle of 60º  Relative active RE during extension for a target angle of 20º  Relative passive RE during extension for a target angle of 60º  Relative passive RE during extension for a target angle of 20º | PatellarT: with an extended knee, one strip was applied across the center of the patella (no stretch), the center of tape as near as possible to the center of the patella and medial and lateral edges of tape aligned with medial., and lateral joint lines  PatellarT not pulled in either medial or lateral direction | Physical Therapist  *Once* | Pre-PatellarT  Post-PatellarT: immediately after with PatellarT | No difference in absolute active RE during extension for target angles 60º, 20º  No difference in absolute passive RE during extension for target angles 60º, 20º  No difference in variable active RE during extension for target angles 60º, 20º  No difference in variable passive RE during extension for target angles 60º, 20º  No difference in relative active RE during extension for target angles 60º, 20º  No difference in relative passive RE during extension for target angles 60º, 20º |
| S Spanos, M Brunswic and E Billis [154]  Pretest-posttest quasi experimental design | Ankle inversion sprain  *Ankle* | N= 20  4F, 16M  (23.1) | AdT: Adhesive tape  NT: No tape | Absolute active RE during plantarflexion for a target angle of 10º  Absolute active RE during plantarflexion for a target angle of 30º  Absolute active RE during inversion for a target angle of 5º  Absolute active RE during inversion for a target angle of 20º | AdT: Applied with closed basket weave with a double heel lock | Physical Therapist  *Once* | Pre-AdT  Post-AdT: immediately after AdT | Significant ↓ in RE with AdT as compared to NT during plantarflexion for a target angle of 10º  Significant ↓ in RE with AdT as compared to NT during plantarflexion for a target angle of 30º  Significant ↓ in RE with AdT as compared to NT during inversion for a target angle of 5º  Significant ↓ in RE with AdT as compared to NT during inversion for a target angle of 20º |
| H Mokhtarinia, TI Ebrahimi, M Salavati, S Goharpai and A Khosravi [155]  Crossover design | Healthy  *Knee* | N= 25  25M  (23.5 ± 3.1) | PatellarT: Patellar tape  NT: No tape | Absolute active RE during flexion at a target angle of 20º  Absolute active RE during flexion at a target angle of 60º  Absolute passive RE during flexion at a target angle of 20º  Absolute passive RE during flexion at a target angle of 60º  TTDPM during knee flexion at target angle 45º with angular velocity 2º/sec | PatellarT: Applied with initial position with the knee extended and quadriceps relaxed, base layer applied from lateral femoral condyle posteriorly to medial femoral condyle, the patella completely covered by base layer, then another layer attached from a slight distance (one thumb’s breath) from the lateral patellar border without pushing the patella, gathering the soft tissue over the medial condyle and adhering to the medial condyle | Physical Therapist  *Once* | Pre-PatellarT  Post-PatellarT: immediately after with PatellarT | Significant ↓ in active RE with PatellarT as compared to NT during flexion at target angle 60º  No difference in active RE with PatellarT as compared to NT during flexion at a target angle of 20º  No difference in passive RE with PatellarT as compared to NT during flexion at target angles 20º, 60º  No difference in TTDPM with PatellarT during flexion at a target angle of 45 º as compared to NT |
|  | Patellofemoral pain syndrome  *Knee* | N= 25  25M  (23.3 ± 3.0) |  |  | PatellarT: Applied similarly as healthy participants, but PatellarT was re-applied until a 50% reduction in visual analogue scale was reported by patients during single leg squat or stair ascending provocation tests |  |  | Significant ↓ in active RE with PatellarT as compared to NT during flexion at a target angle of 60º  No difference in active RE with PatellarT as compared to NT during flexion at a target angle of 20º  No difference in passive RE with PatellarT as compared to NT during flexion at target angles 20º, 60º  No difference in TTDPM with PatellarT during flexion at a target angle of 45 º as compared to NT |
| RS Hinman, KM Crossley, J McConnell and KL Bennell [80] A  Pretest-posttest quasi experimental design | Osteoarthritis  *Knee* | N=18  6F, 12M  (67 ± 7) | LT: Leuko tape  PT: Placebo tape  NT: No tape | Absolute active RE during flexion at a target angle of 20º  Absolute active RE during flexion at a target angle of 40º  Variable active RE during flexion at a target angle of 20º  Variable active RE during flexion at a target angle of 40º | LT: Applied over hypo-allergenic under-tape to re-position patella (medial glide, lateral and anteroposterior tilt) to relieve strain on either an infrapatellar fat pad or pes anserinus (unloading)  PT: PT applied over hypo-allergenic tape similar to LT | Yes  *Once* | Pre-LT  Post-LT: immediately after with LT, after three weeks without LT | No difference in absolute RE with LT as compared to PT and NT during flexion at target angles 20º, 40º  Significant ↑ in constant RE with LT as compared to PT and NT during flexion at a target angle of 40º  No difference in constant RE with LT as compared to PT and NT during flexion at a target angle of 20º |
| RS Hinman, KM Crossley, J McConnell and KL Bennell [80] B  Randomized controlled design |  | N= 87  30F, 57M  (69 ± 9) |  |  |  | Yes  *Three times (once every week)* | Three weeks | No difference in absolute RE with LT as compared to PT and NT during flexion at target angles 20º, 40º  No difference in constant RE with LT as compared to PT and NT during flexion at target angles 20º, 40º |
| T Halseth, WM John and D Mark [104]  Pretest-posttest quasi experimental design | Healthy  *Ankle* | N=30  15F, 15M  (18 to 30) | KT: Kinesio tape  NT: No tape | Absolute active RE during plantarflexion for target angle between 1º and 35º  Absolute active RE for inversion with plantarflexion for target angle between 1º and 10º  Constant active RE during plantarflexion for target angle between 1º and 35º  Constant active RE for inversion with plantarflexion for target angle between 1º and 10º | KT: Applied according to Kase’s technique [24] in four strips, initial position with the ankle in slight plantarflexion  Strip 1: Applied on anterior mid-foot (115-120% stretch), attached below anterior tibial tuberosity over tibialis anterior  Strip 2: Applied above the medial malleolus, the wrapped around heel like a stirrup, attached just lateral to strip 1  Strip 3: Applied across the ankle anteriorly, covering both lateral and medial malleoli  Strip 4: Applied on the arch with a slight stretch (4-6 inches) above both lateral and medial malleoli | Athletic trainer  *Once* | Pre-KT  Post-KT: immediately after KT | No difference in absolute RE with KT as compared to NT during plantarflexion for a target angle between 1º and 35º  No difference in absolute RE with KT as compared to NT during inversion with plantarflexion for a target angle between 1º and 10º  No difference in constant RE with KT as compared to NT during plantarflexion for a target angle between 1º and 35º  No difference in constant RE with KT as compared to NT during inversion with plantarflexion for a target angle between 1º and 10º |
| K Mumford [62]  Crossover design | Chronic inversion injury  *Ankle* | LT: N= 10  10F  (23.3 ± 2.6)  GT: N= 10  6F, 4M  (23.3 ± 2.6) | LT: Leuko tape  GT: Gibney tape | Absolute active RE during inversion  Absolute active RE during eversion  Constant active RE during inversion  Constant active RE during eversion  Variable active RE during inversion  Variable active RE during eversion | LT: applied using a series of three stirrups, a figure of six shapes and a heel lock  GT: Applied with Gibney-basket-weave technique with a series of alternating stirrups and horizontal strips | Not reported  *Once* | Pre-LT/GT  Post-LT/GT: immediately after with LT/GT, 10 minutes after with LT/GT, 20 minutes after with LT/GT | Significant ↑ in constant RE with LT as compared to GT during eversion after 10 minutes of tape application  No difference in absolute active RE with either LT or GT during inversion or eversion at all assessment points  No difference in constant active RE with either LT or GT during inversion, eversion at all immediately after and 20 minutes after tape application  No difference in variable active RE with either LT or GT during inversion or eversion at all assessment points |
| AL Cecchinato [54]  Randomized controlled design | Functional ankle instability (fatigue)  *Ankle* | AT: N=10  ?F, ?M  (?)  NT: 10  ?F, ?M  (?)  *Brace: N= 11  ?F, ?M  (?) | AT: Athletic tape  NT: No tape  Brace | Absolute RE during full weight bearing at target angles between 0º to 6º  Absolute RE during full weight bearing at target angles between 7º to 13º  Absolute RE during full weight bearing at target angles between 14º to 20º  Pre/post (fatigue) | AT: Closed-basket weave ankle tape protocol with heel and lace pads  Skin prepared with adhesive spray and heel lace pads placed on tibiotalar joint anteriorly and posteriorly on posterior calcaneus and Achilles tendon  Pre-wrap covered foot and ankle from mid-arch to distal gastrocnemius  AT applied with two anchors placed on pre-wrap at distal gastrocnemius, then an alternating pattern of three medial to lateral stirrups and horseshoes were applied, anchors and horseshoes were used to cover gaps, then three sets of medial to lateral heel locks were applied | Not reported  *Once* | Pre-AT  Post-AT: immediately after AT, after fatigue protocol with KT | No difference in RE with AT as compared to NT during full weight bearing at target angles between 0º to 6º  No difference in RE with AT as compared to NT during full weight bearing at target angles between 7º to 13º  No difference in RE with AT as compared to NT during full weight bearing at target angles between 14º to 20º |
| MJ Callaghan, J Selfe, PJ Bagley and JA Oldham [55]  Randomized crossover design | Healthy  *Knee* | N=52  27F, 25M  (23.2 ± 4.6) | PatellarT: Patellar tape  NT: No tape | Absolute active RE during flexion at a target angle of 45º  Absolute passive RE during flexion at a target angle of 45º  TTDPM during flexion at target angle 90º with angular velocity 30º/sec | PatellarT: with extended knee, one strip applied across center of patella (no stretch), center of tape as near as possible to center of patella and medial, lateral edges of tape aligned with medial., lateral joint lines  PatellarT not pulled in either medial or lateral direction | Physical Therapist  *Once* | Pre-PatellarT  Post-PatellarT: immediately after with PatellarT | No difference in TTDPM with PatellarT as compared to NT during flexion  No difference in RE with PatellarT as compared to NT during active flexion at target angle 45º  No difference in RE with PatellarT as compared to NT during passive flexion at target angle 45º |
| TJ Hubbard and TW Kaminski [156]  Randomized crossover design | Healthy  Functional ankle instability  *Ankle* | N= 16  8F, 8M  (21.6 ± 1.7) | AdT: Adhesive tape with closed basket weave  NT: No tape  *Ankle LOK brace  *Aircast air-stirrup brace | TTDPM during inversion/ eversion | AdT: Applied with closed basket weave technique | Not reported  *Once* | Pre-AdT  Post-AdT: immediately after AdT | No difference in TTDPM with AdT as compared to NT during inversion/eversion with an unstable and uninjured ankle |
| TW Kaminski and TM Gerlach [157]  Randomized crossover design | Healthy  *Ankle* | N=20  20F  (20.8 ± 2.7) | GT: Gibney tape  NT: No tape  *Neoprene brace | Absolute active RE during eversion at target angle 10º  Absolute active RE at the subtalar neutral position at 0º  Absolute active RE during inversion at a target angle of 20º  Absolute passive RE during inversion at a target angle of 30º  Absolute passive RE during eversion at a target angle of 10º  Absolute passive RE at the subtalar neutral position at 0º  Absolute passive RE during inversion at a target angle of 20º  Absolute passive RE during inversion at a target angle of 30º | GT: Applied with closed basket-weave technique, an adhesive tape was applied over taping under wrap base | Athletic trainer  *Once* | Pre-GT  Post-GT: immediately after GT | No difference in active RE with GT as compared to NT during all positions and target angles  No difference in passive RE with GT as compared to NT during all positions and target angles |
| KM Refshauge, SL Kilbreath and J Raymond [56]  Randomized controlled design | Healthy  *Ankle* | N= 25  ?F, ?M  (21.3 ± 4.4) | LT: Leuko tape  NT: No tape | TTDPM (70%) during small dorsiflexion/plantarflexion with angular velocity 0.1º/sec  TTDPM (70%) during small dorsiflexion/plantarflexion with angular velocity 0.5º/sec  TTDPM (70%) during small dorsiflexion/plantarflexion with angular velocity 2.5º/sec | LT: Applied with a basket-weave technique with a combination of heel locks held by three anchors, stirrups, and figure of six application | Not reported  *Once* | Pre LT  Post-LT: immediately after with LT | No difference in TTDPM during dorsiflexion/plantarflexion perception with LT as compared to NT for angular velocities 0.1º/sec, 0.5º/sec, 2.5º/sec |
|  | Ankle inversion sprain  *Ankle* | N= 18  ?F, ?M  (21.6 ± 5.0) |  |  |  |  |  | No difference in TTDPM during dorsiflexion/plantarflexion perception with LT as compared to NT for angular velocities 0.1º/sec, 0.5º/sec, 2.5º/sec |
| GG Simoneau, RM Degner, CA Kramper and KH Kittleson [158]  Randomized crossover design | Healthy  *Ankle* | N= 20  20M  (20.3 ± 1.5) | AT: Athletic tape  NT: No tape | Absolute passive RE during plantarflexion for target angle 10º in the open kinetic chain  Absolute passive RE during dorsiflexion for target angle 5º in the open kinetic chain  Absolute passive RE during plantarflexion for target angle 10º in the close kinetic chain  Absolute passive RE during dorsiflexion for target angle 5º in the close kinetic chain  TTDPM during dorsiflexion at 0.25º/sec in the open kinetic chain  TTDPM during plantarflexion at 0.25º/sec in the open kinetic chain  TTDPM during dorsiflexion at 0.25º/sec in the closed kinetic chain  TTDPM during plantarflexion at 0.25º/sec in the closed kinetic chain | AT: Two 12.7 cm strips were applied in two steps to provide cutaneous stimulation without mechanical constriction and pressure  Step 1: Applied approximately 7.6 cm proximal to the ankle joint line and ended 5.1 cm distal to the ankle joint line, AT was applied directly on the skin over the anterior aspect of the ankle joint  Step 2: Applied posteriorly over the Achilles tendon and calcaneus | Not reported  *Once* | Pre-AT  Post-AT: immediately after AT | Significant ↓ in RE during plantarflexion for target angle 10º in the open kinetic chain  Significant ↓ in RE during dorsiflexion for target angle 10º in the open kinetic chain  No difference in RE during plantarflexion for a target angle of 10º in the closed kinetic chain  No difference in RE during dorsiflexion for a target angle of 5º in the closed kinetic chain  No difference in TTDPM during plantarflexion in the open kinetic chain  No difference in TTDPM during dorsiflexion in the open kinetic chain  No difference in TTDPM during plantarflexion in the closed kinetic chain  No difference in TTDPM during dorsiflexion in the closed kinetic chain |
| EJ Heit, SM Lephart and SL Rozzi [32]  Crossover design | Healthy  *Ankle* | N= 26  16F, 10M  18.9 ± 0.8 | AT: Athletic tape  NT: no tape  *Bracing: Swede-O brace | Absolute active RE during plantarflexion for target angle 30º  Absolute active RE during inversion for target angle 15º | AT: Combination of methods of [159] and [160], applied over an under-wrap with two proximal and one distal circumferential anchoring strips, remained of AT consisted of four stirrups overlapped by half and anchored with a proximal circumferential strip, four horse shoe strips overlapped by one-half, two alternating figure-of-eight, circumferential strips, and continuous medial-lateral heel locks | Athletic trainer  *Once* | Pre-AT  Post-AT: immediately after AT | Significant ↓ in absolute RE with AT as compared to NT during plantarflexion for target angle 30º  Significant ↓ in absolute RE with AT as compared to NT during inversion for target angle 15º |
| J Jerosch, I Hoffstetter, H Bork and M Bischof [29]  Crossover design | Healthy  *Ankle* | N=14  ?F, ?M  (23.9 + 2.83) | GT: Gibney tape  NT: No tape  *Mikros-lace on brace  *Aircast stirrup brace | Absolute active RE during inversion at target angle 5º  Absolute active RE during inversion at a target angle of 15º  Absolute active RE during inversion at target angle 20º | GT: Applied with the basket-weave technique with stabilizing effect provided with a figure-of-eight bandage | Physical Therapist  *Once* | Pre-GT  Post-GT: immediately after GT | Significant ↓ in RE with GT as compared to NT during inversion at target angles 5º, 15º, 20º |
|  | Ankle instability  *Ankle* | N=16  ?F, ?M  (23.9 + 2.83) |  |  |  |  |  | Significant ↓ in RE with GT as compared to NT during inversion at target angles 5º, 15º, 20º |
| S Robbins, E Waked and R Rappel [161]  Randomized crossover design | Healthy  *Ankle* | GT: N= 12  ?F, ?M  (26.6)  NT: N= 12  ?F, ?M  (26.6) | GT: Gibney tape  NT: No tape | Absolute RE during foot position sense testing at surface slope 0-10º  Absolute RE during foot position sense testing at surface slope 10-20º  Absolute RE during foot position sense testing at surface slope >20º  Net RE during foot position sense testing at surface slope 0-10º  Net RE during foot position sense testing at surface slope 10-20º  Net RE during foot position sense testing at surface slope >20º | GT: Applied with the basket-weave technique with stabilizing effect provided with a figure-of-eight bandage | Physical Therapist  *Once* | Pre-GT  Post-GT: immediately after GT, 30 minutes post GT | Significant ↓ in absolute RE with GT as compared to NT during foot position sense testing at surface slope >20º at baseline and post 30 minutes of GT application  No difference in absolute, net RE with GT as compared to NT during foot position sense testing at surface slope 0-10º at baseline and post 30 minutes of GT application  No difference in absolute, net RE with GT as compared to NT during foot position sense testing at surface slope 10-20º at baseline and post 30 minutes of GT application  Significant ↑ in net RE with GT as compared to NT during foot position sense testing at surface slope >20º at baseline and post 30 minutes of GT application |
|  | Healthy (fatigue)  *Ankle* |  |  |  |  |  | Pre-GT  Post-GT: 30 minutes post fatigue protocol with GT | Significant ↓ in absolute RE with GT as compared to NT during foot position sense testing at surface slope >20º at baseline and post 30 minutes of GT application  No difference in absolute, net RE with GT as compared to NT during foot position sense testing at surface slope 0-10º at baseline and post 30 minutes of GT application  No difference in absolute, net RE with GT as compared to NT during foot position sense testing at surface slope 10-20º at baseline and post 30 minutes of GT application  Significant ↑ in net RE with GT as compared to NT during foot position sense testing at surface slope >20º at baseline and post 30 minutes of GT application |
| M Schenker [162]  Crossover design | Ankle sprain (sports personnel)  *Ankle* | N= 16  6F, 10M  (18.9 ± 2.3) | LT: Leuko tape  NT: No tape  *Mikros brace | Absolute active RE during plantarflexion | - | Not reported  - | Pre-LT  Post-LT: - | No difference in RE with LT as compared to NT during plantarflexion on foot 1 and foot 2 |
| ASIS: Anterior superior iliac spine, ACL: Anterior cruciate ligament, AMEDA: Active movement extent discrimination apparatus, BT: Balance training with wobble board, COP: Centre of pressure, PSIS: Posterior superior iliac spine, RCT: Randomized controlled trial, RE: Repositioning error, TTDPM: Threshold to detect passive movement, *: Knee orthosis not considered as a comparator for this study | | | | | | | | |

**Figures**

Figure S1. Forest plot illustrating the effect of taping on repositioning error (randomized design trials). A positive effect size meant reduced repositioning error for no taping comparator group, whereas a negative overall effect size indicates reduced repositioning error for taping group.

Figure S2. Forest plot illustrating the effect of taping on repositioning error (non-randomized design trials). A positive effect size meant reduced repositioning error for no taping comparator group, whereas a negative overall effect size indicates reduced repositioning error for taping group.

Figure S3. Forest plot illustrating the effect of elastic taping on repositioning error. A positive effect size meant reduced repositioning error for no taping comparator group, whereas a negative overall effect size indicates reduced repositioning error for elastic taping group.

Figure S4. Forest plot illustrating the effect of rigid taping on repositioning error. A positive effect size meant reduced repositioning error for no taping comparator group, whereas a negative overall effect size indicates reduced repositioning error for rigid taping group.

Figure S5. Forest plot illustrating the effect of taping on repositioning error on different population groups. A positive effect size meant reduced repositioning error for no taping comparator group, whereas a negative overall effect size indicates reduced repositioning error for taping group.

Figure S6. Forest plot illustrating the effect of elastic taping on repositioning error on different population groups. A positive effect size meant reduced repositioning error for no taping comparator group, whereas a negative overall effect size indicates reduced repositioning error for elastic taping group.

Figure S7. Forest plot illustrating the effect of rigid taping on repositioning error on different population groups. A positive effect size meant reduced repositioning error for no taping comparator group, whereas a negative overall effect size indicates reduced repositioning error for rigid taping group.

Figure S8. Forest plot illustrating the effect of taping on repositioning error on different population groups. A positive effect size meant reduced repositioning error for placebo taping comparator group, whereas a negative overall effect size indicates reduced repositioning error for taping group.

Figure S9. Forest plot illustrating the effect of taping on repositioning error on different population groups (randomized design trials). A positive effect size meant reduced repositioning error for placebo taping comparator group, whereas a negative overall effect size indicates reduced repositioning error for taping group.

Figure S10. Forest plot illustrating the effect of taping on repositioning error on different population groups (non-randomized design trials). A positive effect size meant reduced repositioning error for placebo taping comparator group, whereas a negative overall effect size indicates reduced repositioning error for taping group.

Figure S11. Forest plot illustrating the effect of elastic taping on repositioning error on different population groups. A positive effect size meant reduced repositioning error for placebo taping comparator group, whereas a negative overall effect size indicates reduced repositioning error for elastic taping group.

Figure S12. Forest plot illustrating the effect of rigid taping on repositioning error on different population groups. A positive effect size meant reduced repositioning error for placebo taping comparator group, whereas a negative overall effect size indicates reduced repositioning error for rigid taping group.

Figure S13. Forest plot illustrating the effect of taping on repositioning error on different population groups. A positive effect size meant reduced repositioning error for placebo taping comparator group, whereas a negative overall effect size indicates reduced repositioning error for taping group.

Figure S14. Forest plot illustrating the effect of elastic taping on repositioning error on different population groups. A positive effect size meant reduced repositioning error for placebo taping comparator group, whereas a negative overall effect size indicates reduced repositioning error for elastic taping group.

Figure S15. Forest plot illustrating the effect of taping on threshold to detection of passive motion. A positive effect size meant reduced threshold to detection for no taping comparator group, whereas a negative overall effect size indicates reduced threshold to detection for taping group.

Figure S16. Forest plot illustrating the effect of elastic/rigid taping on threshold to detection of passive motion. A positive effect size meant reduced threshold to detection for no taping comparator group, whereas a negative overall effect size indicates reduced threshold to detection for elastic/rigid taping group.

Figure S17. Forest plot illustrating the effect of taping on threshold to detection of passive motion on different population groups. A positive effect size meant reduced threshold to detection for no taping comparator group, whereas a negative overall effect size indicates reduced threshold to detection for taping group.

Figure S18. Forest plot illustrating the effect of rigid taping on threshold to detection of passive motion on different population groups. A positive effect size meant reduced threshold to detection for no taping comparator group, whereas a negative overall effect size indicates reduced threshold to detection for rigid taping group.

Figure S19. Forest plot illustrating the effect of taping on threshold to detection of passive motion. A positive effect size meant reduced threshold to detection for placebo taping comparator group, whereas a negative overall effect size indicates reduced threshold to detection for taping group.

Figure S20. Forest plot illustrating the effect of taping on active movement extent discrimination. A positive effect size indicated increased active movement extent discrimination for no taping comparator group, whereas a negative overall effect size increased active movement extent discrimination for taping group.

Figure S21. Forest plot illustrating the effect of elastic taping on active movement extent discrimination. A positive effect size indicated increased active movement extent discrimination for no taping comparator group, whereas a negative overall effect size increased active movement extent discrimination for elastic taping group.

Figure S22. Forest plot illustrating the effect of taping on active movement extent discrimination on different population group. A positive effect size indicated increased active movement extent discrimination for no taping comparator group, whereas a negative overall effect size increased active movement extent discrimination for taping group.

Figure S23. Forest plot illustrating the effect of elastic taping on active movement extent discrimination on different population group. A positive effect size indicated increased active movement extent discrimination for no taping comparator group, whereas a negative overall effect size increased active movement extent discrimination for elastic taping group.

Figure S24. Forest plot illustrating the within group effect of taping on repositioning error. A positive effect size indicated deteriorated repositioning error, whereas a negative overall effect size indicated improved repositioning error.

Figure S25. Forest plot illustrating the within group effect of taping on repositioning error (randomized design). A positive effect size indicated deteriorated repositioning error, whereas a negative overall effect size indicated improved repositioning error.

Figure S26. Forest plot illustrating the within group effect of taping on repositioning error (non-randomized design). A positive effect size indicated deteriorated repositioning error, whereas a negative overall effect size indicated improved repositioning error.

Figure S27. Forest plot illustrating the within group effect of elastic and rigid taping on repositioning error. A positive effect size indicated deteriorated repositioning error, whereas a negative overall effect size indicated improved repositioning error.

Figure S28. Forest plot illustrating the within group effect of taping on repositioning error in different population groups. A positive effect size indicated deteriorated repositioning error, whereas a negative overall effect size indicated improved repositioning error.

Figure S29. Forest plot illustrating the within group effect of elastic taping on repositioning error in different population groups. A positive effect size indicated deteriorated repositioning error, whereas a negative overall effect size indicated improved repositioning error.

Figure S30. Forest plot illustrating the within group effect of rigid taping on repositioning error in different population groups. A positive effect size indicated deteriorated repositioning error, whereas a negative overall effect size indicated improved repositioning error.

Figure S31. Forest plot illustrating the within group effect of taping on threshold to detection of passive motion. A positive effect size indicated deteriorated threshold to detection of passive motion, whereas a negative overall effect size indicated improved threshold to detection of passive motion.

Figure S32. Forest plot illustrating the within group effect of taping on threshold to detection of passive motion (randomized designs). A positive effect size indicated deteriorated threshold to detection of passive motion, whereas a negative overall effect size indicated improved threshold to detection of passive motion.

Figure S33. Forest plot illustrating the within group effect of taping on threshold to detection of passive motion (non-randomized designs). A positive effect size indicated deteriorated threshold to detection of passive motion, whereas a negative overall effect size indicated improved threshold to detection of passive motion.

Figure S34. Forest plot illustrating the within group effect of elastic taping on threshold to detection of passive motion. A positive effect size indicated deteriorated threshold to detection of passive motion, whereas a negative overall effect size indicated improved threshold to detection of passive motion.

Figure S35. Forest plot illustrating the within group effect of taping on threshold to detection of passive motion on different population groups. A positive effect size indicated deteriorated threshold to detection of passive motion, whereas a negative overall effect size indicated improved threshold to detection of passive motion.
